# Supplementary material for: Study protocol: quantitative fibronectin to help decision-making in women with symptoms of preterm labour (QUIDS) part 2, UK Prospective Cohort Study
Source: BMJ Open. 2018 Apr 19;8(4):e020795. doi: 10.1136/bmjopen-2017-020795 (PMC5914783; doi:10.1136/bmjopen-2017-020795)
Supplement: Supplementary file 1 [file bmjopen-2017-020795supp001.pdf]

QUIDS Qualitative

Quantitative Fibronectin to help Decision-making in women with Symptoms of Preterm Labour:

determining decisional requirements

Protocol

(October 2015)

White H, O’Brien E, Hodgetts-Moreton V, Stock S, Lavender T

Background

Preterm birth, defined as birth prior to 37 weeks gestation, occurs in 6-7% of pregnancies in Europe[1] and was recorded as 5.78% in England in 2013/14, equating to over 37,000 births.[2] Preterm birth is associated with a high risk of mortality, wide-ranging short- and long-term morbidities,[3,4] and significant economic costs to the NHS compared with birth at term.[5] Reducing the detrimental impact of preterm birth relies on the provision of timely and appropriate perinatal interventions. However, accurate prediction of preterm birth is challenging, even when the clinical symptoms are suggestive of preterm labour. In randomised trials approximately 80% of women diagnosed with preterm labour remained pregnant after 7 days.[6,7]

Interventions in preterm labour and preparations for preterm birth may include administration of corticosteroids to accelerate fetal lung maturation[8,9] and magnesium sulphate for fetal neuroprotection,[10] in utero transfer to a facility with appropriate maternity and neonatal services, and tocolysis to optimise time before birth to enable these.[11] Whilst such interventions can improve outcomes for mothers and babies who do experience preterm birth, they are not necessarily benign, especially for those in whom preterm birth does not occur.

The maximal beneficial impact of corticosteroids occurs with administration between 48 hours and seven days before birth, thus timing is especially important in optimising benefit for the neonate. For women who remain at risk of preterm birth after seven days of the initial dose, repeated doses reduce respiratory distress in the neonate[9] but have been found to be associated with a dose-dependent reduction in birthweight.[12,13] A five-year follow-up study of women who received repeated doses of antenatal corticosteroids due to risk of preterm birth found an increased risk of neurodevelopment impairment in infants born at term.[14] Therefore developing a strategy to establish the optimal time to give steroids is a research priority.

Magnesium sulphate administration immediately prior to birth has been shown to reduce cerebral palsy,[10] but there is a risk of magnesium toxicity leading to respiratory depression in the mother and, theoretically, the neonate.[15]

Whilst there is no clear beneficial effect of tocolytics on the incidence or outcome of preterm birth,[16] their use is recommended if the days gained prior to preterm birth can be used appropriately, for example transfer to a suitable maternity unit or the administration of drugs to protect the neonate.[11] Tocolysis is linked with various maternal and neonatal complications,[17] hence the need for therapy targeted only for those at risk of preterm birth and close monitoring of the mother and fetus throughout.

Often, inpatient admission is recommended if preterm labour is suspected. Previous literature has highlighted the social isolation and support needs that women with high-risk pregnancies who are hospitalised experience.[18] In some cases, in-utero transfer is indicated to ensure that birth takes place in a specialist unit with appropriate neonatal care facilities. This policy has been shown to reduce mortality[19,20] and morbidity[21] in preterm neonates, especially those born very premature. Qualitative research has indicated that women generally acknowledge the potential benefit of in utero transfer to their baby and, hence, are willing to endure the inconvenience and upheaval that it entails.[22,23] However, the experience is associated with an emotional, social and financial burden on women and their families, especially for the substantial proportion of women who do not deliver prematurely following in utero transfer. When describing their experiences of in utero transfer, women expressed shock at the prospect of the transfer, feeling socially isolated, and having no control over the situation, in addition to the practical difficulties experienced particularly by women who already had children.[22,24,25] In a large survey of women who had experienced in utero transfer, over a quarter lamented the financial cost[24] particularly with respect to their partner's outlay for travel, food, accommodation, and phone bills, exacerbated with requiring time off work.[22] Furthermore, in utero transfer is costly to maternity services. Securing a maternal and neonatal bed

in another unit is a time-consuming task that often falls to delivery suite midwives to arrange, whilst also continuing to provide care to the woman.[26] In a large observational study of all in utero transfers that took place in Scotland in a six-month period, nearly one third of all transfers were due to threatened preterm labour.[27] Under half of the women transferred from one consultant-led unit to another gave birth within 48 hours.[27] Such unnecessary transfers are costly to women, their families and maternity services. Qualitative research into women's experiences of preterm labour have highlighted the need for caregivers to create an environment where women are enabled to discuss their fears[28] and exert control over how they manage their preterm labour care.[25]

Accurate prediction of preterm birth could reduce the burdens and risks associated with unnecessary interventions, and enable women and their clinicians to make informed decisions regarding their care. Numerous diagnostic tests have been used in preterm labour, including biochemical tests of vaginal secretions and cervical length.[29] One such test is fetal fibronectin, a near-bedside test that provides a positive or negative result and has excellent negative predictive value.[30] Thus fetal fibronectin can identify which women will not benefit and may be put at risk by the interventions described previously, and reduce costs to maternity services.[31] Developments in fetal fibronectin testing have led to a quantitative test that provides a concentration of fetal fibronectin in vaginal secretions, giving women and clinicians more information on which to base their management decisions.[32]

Qualitative evidence has indicated that women feel a sense of increased responsibility to their babies and themselves during a high risk pregnancy, such as threatened preterm labour.[33] Women want to be involved in decision making about their care to different degrees and feel most satisfied when their caregiver supports them to make decisions in the way they felt most comfortable.[33]

Previous literature on decision making and preterm birth has focussed on diagnostic tests[6,28–32,34] and the care of the preterm infant.[35,36] To date, there has been no investigation of what women, their partners and caregivers would like to know in order to make informed decisions about the care that is provided following the signs and symptoms of preterm labour.

Funding has been received from the National Institute for Health Research Health Technology Assessment Programme for a large, multicentre trial to develop a mobile application decision support tool for the management of women with symptoms and signs of preterm labour, based on a validated model using quantitative fetal fibronectin testing. This study is the precursor to that trial, with the aim of determining the decisional needs of pregnant women with the symptoms and signs of preterm labour, their families and caregivers, using a qualitative framework approach. The outcomes of this qualitative study will inform the development of the mobile application decision support tool, using the findings from an individual patient data meta-analysis. The tool will then be externally validated and refined in the multi-centre trial, QUIDS.

Methods

A qualitative framework approach will be used, based on data collected from focus groups and semi-structured telephone interviews.

Setting

Focus groups will take place in three maternity units: Liverpool Women's NHS Foundation Trust, Birmingham Women's NHS Foundation Trust and Royal Edinburgh Hospital, NHS Lothian. There will be focus groups for women and a separate focus group for partners. Clinicians who care for women with threatened preterm birth will be interviewed by telephone.

Sample

A purposive sample of women and partners will be recruited to cover a variety of experiences of preterm labour and birth. Women will be stratified by their prior experience and relevant characteristics, including ethnicity, previous obstetric history, living in an urban or rural setting and proximity to a tertiary neonatal referral centre. Two focus groups of 4–8 women will be conducted at each site; one for pregnant women who are at high risk of preterm birth, and one for postnatal women who have recently experienced preterm birth. One partners' focus group will be conducted at one of the sites. If women or partners are unable to attend a focus group but still wish to participate, a semi-structured telephone interview will be offered.

Up to 10 obstetricians, including trainees, midwives, and neonatologists will be purposefully recruited to cover a range of professional backgrounds and experience. Semi-structured telephone interviews will be used to collect the data.

Eligibility

Principal inclusion criteria for women's antenatal focus groups

Women who are currently pregnant who:

- Have previously experienced preterm birth following preterm labour,
- Have experienced threatened preterm labour in this pregnancy,
- Are at high risk of preterm birth for another clinical reason, such as prior cervical surgery.

Principal inclusion criteria for women's postnatal focus groups

Women who have experienced preterm birth following preterm labour at <34 weeks whose babies **are stable and well** and are receiving care on the special care baby unit or neonatal intensive care unit.

Principal inclusion criteria for partners' focus groups

Partners of women who fit the eligibility criteria for either focus group.

Principal exclusion criteria for the focus groups

Non-English speaking individuals.

Principal inclusion criteria for clinician interviews

Clinicians who care for pregnant women i.e. obstetricians (including trainees), neonatologists and midwives.

Principal exclusion criteria for clinician interviews

Researchers in QUIDS or QUIDS qualitative.

Recruitment

Women and partners

Eligible women will be identified by clinicians in the preterm birth clinic and other antenatal clinics, and antenatal, triage or labour wards (for the antenatal focus groups) and the special care baby unit or postnatal clinics (for the postnatal focus groups) at each site. Eligible partners will be identified by the same method. Clinicians who are aware of and understand the research aims will approach women and partners to request consent for a researcher to contact them. Importantly, only postnatal parents whose babies are being cared for on the SCBU who are considered stable and well by the clinicians will be approached. With consent the researcher will make contact to talk to the women and/or their partners about the research, either face-to-face or over the telephone. Potential participants will be given the participant information sheet (PIS) (appendix _) that is relevant to them and given verbal information about the study. Each participant will be given time to read the information and the opportunity to have any questions answered. Willing participants will be asked to provide their written consent prior to the focus groups.

Clinicians

Eligible clinicians will be approached by the researchers, via email or face-to-face. Clinicians will be given the clinician PIS (appendix _) and the opportunity to read the information and have any questions answered. Willing clinicians will be asked to provide their written consent prior to the interviews.

All participants (women, partners and clinicians) will be reassured that they are not compelled to participate, that they can withdraw from the study at any time, and that non-participation will not affect their care or employment in any way.

Data collection

The primary aim of this research is to determine the decisional requirements of women, their partners and clinicians for the management of preterm labour. Qualitative semi-structured interviews, in a focus-group setting or individual telephone interviews, provide a means of collecting rich, in-depth data with a specific focus.[37] Hence, structured topic guides will be used to initiate and concentrate the discussion (appendices 7–10).

Focus groups are the preferred format for eliciting the view of women and women's partners. Encouraging discussion among a homogenous group with a shared interest is likely to provide rich insight and understanding into the group's experiences, beliefs and norms as a result of their social interaction.[38] Conversely, interviewing clinicians individually avoids the potential pitfall of professional embarrassment stifling ideas in a group setting. Interviewing individual clinicians with a range of professional experience should ensure that the decisional requirements of clinicians at all levels of experience are understood.

Demographic details and baseline characteristics will be collected prior to the interviews, either as a self-completion questionnaire, or questions asked by the researcher over the telephone. All interviews will be audio recorded, with the participants' consent, and field notes taken. The focus groups will be facilitated by at least two researchers. This is to ensure that all pre-specified areas of interest are covered and that non-verbal communication and group interactions are documented within the field-notes, which will provide context for the data analysis. Recapping will be used to

clarify aspects and avoid misinterpretation. To enable all participants to talk freely, the researchers will be unknown to the participants and not working clinically in the unit where the interview is conducted. Clinicians will be interviewed by a researcher who is unknown to them.

	Site	Interviewers
Women and partners' focus groups	Liverpool	HW and EO
	Birmingham	HW and VH-M
	Edinburgh	HW and LM
Clinician interviews	Telephone	HW (and EO?)

Analysis plan

A framework approach to data analysis will be used. This approach was developed to manage and interpret large volumes of data collected to inform health policy, meaning they had focussed aims and objectives.[37] Likewise, this research has clear aims, as described previously, in addition to the methodological aim of collecting rich data about the experiences and beliefs of women, their partners and clinicians in relation to managing preterm labour.

Framework analysis follows specific, clearly documented stages of analysis that are transparent so that others can review the interpretation processes and understand how the findings were reached.[39] Transparency is particularly important in this study as the findings will inform the development of an application to aid management decisions in clinical practice. Following verbatim transcription of the interview recordings, the researchers will become familiar with the data by reading the transcripts and field-notes several times. The next stage is to develop a theoretical framework by re-reading the transcripts and making notes as recurring characteristics are

recognised. The characteristics will then be collated into themes, which are based on the text itself, supported by the field-notes. The resulting thematic framework will be applied back to the transcripts and field-notes to check that it reflects the context of the original data. The transcripts will be coded, so that portions of text are linked to a discrete theme. A sample of transcripts will be independently coded by two people. The data will be charted and indexed to identify the preterm labour or professional experience of the participant, thus enabling the attribution of themes to a particular group. Finally, the content of the charts will be interpreted and mapped against each other to devise themes and sub-themes categories. Once again, this will involve review of the original data. Explanatory accounts will be developed to clarify the data and quotable sections of data will be identified. The final categories will be discussed between the researchers until consensus is met. The researchers will maintain reflexive journals throughout the data collection and analysis stages, recognising and ameliorating, as far as possible, the fact that their presence and assumptions impact on the data and the findings.[40]

This method of data analysis creates a clear audit trail thus ensuring rigour. Each stage of analysis refers back to the original data so that context and meaning is not lost in the final framework of themes and subthemes. The data analysis process will be managed using NVivo software, a qualitative data analysis tool.

Participant withdrawal

Participants may withdraw from the study at any point. However, they will not be able to withdraw use of their data once the prognostic tool is developed.

Safety

The physical safety of participants will be ensured through adhering to the health and safety policies of the host units where the focus groups take place.

The emotional wellbeing of the participants will be safeguarded by following the Distress Policy (see appendix 11). The Supervisors of Midwives (SOM) team in each unit will be informed of the study and women and their partners will be given the SOM team contact details, should they become distressed or upset as a result of talking about their experiences. Participants will also be given the contact details for accessing local counselling services.

Good clinical practice

Informed consent

All participants will be fully informed about the study and the subsequent QUIDS trial via verbal and written communication. All eligible individuals will be given the participant information sheet (appendix __) and provided with an opportunity to have any questions answered. Written consent will then be gained prior to the commencement of the focus groups/interviews.

Confidentiality

Demographic information will be collected from participants to attribute themes from the data to particular groups within the analysis and dissemination of findings. Demographic information, which will contain potentially identifiable information, will be kept in a secure lockable cabinet. Audio recordings will be stored on an encryptable audio device only until they are transcribed. Once transcribed the audio recordings will be deleted. Transcription services are provided by '1st Class Secretarial', who subscribe to the Data Protection Act and have also signed the Code of Practice on Data Handling. Hard copies of audio transcripts and field-notes will be kept in a separate secure

lockable cabinet to the demographic information. The transcripts and field-notes will be coded to identify which participant provided that data; the codes will only be known by the researchers. Participant's data will not be used for any purpose other than this study and the subsequent QUIDS trial.

Data Protection

Participants will be informed that publications from this study will contain direct quotes from the focus groups/interviews and categorisation of their experience of preterm labour (e.g. experienced preterm birth), which could enable personal identification.

All researchers involved in this study must comply with the requirements of the Data Protection Act 1998 with regard to the collection, storage, processing and disclosure of personal information and uphold the Act's core principles. All computers used for processing data are password protected and subject to the strict data protection policies of the researcher's institution.

Good clinical practice training

All researchers involved in this study must hold evidence of recent Good Clinical Practice training.

Additional ethical considerations

Expenses and reimbursement

Participants will be reimbursed for all out of pocket expenses, for example travelling to the interview site. Participants will be informed of this and how to apply for expenses reimbursement, including keeping receipts for travel.

Safety of researchers

An individualised risk assessment will be conducted to identify any risks to researchers or participants involved in this study. The lone working policy of the institution will be adhered to at all times. The only anticipated lone working will be during travel to and from the interview sites.

The lone working policy of the researcher's institutions mandates that researchers wear a GPS tracking and audio transmitting device during all lone-working, off-site research activity with participants. Participants will be informed if this device is being used.

Insurance / Indemnity

The researcher's institution holds public liability insurance and professional indemnity insurance (appendices 12, 13 and 14).

Timeline

The anticipated start date for the focus groups and interviews is 1st January 2016, to be completed within 3 months.

Appendices

Appendix 1: PIS women

Appendix 2: PIS partners

Appendix 3: PIS clinicians

Appendix 4: Consent form women

Appendix 5: consent form partners

Appendix 6: consent form clinicians

Appendix 7: Interview schedule AN women

Appendix 8: Interview schedule PN women

Appendix 9: Interview schedule partners

Appendix 10: Interview schedule clinicians

Appendix 11: Distress policy

Distress

- Participant indicates that they are experiencing high levels of stress, anxiety or emotional distress
- Participant exhibits signs suggestive of excessive stress anxiety or emotional distress e.g. shaking, uncontrolled crying

Response

- Stop interview / discussion
- Researcher (health professional) to offer immediate support
- Assess mental state - ASK
    - Tell me what thought you are having?
    - Tell me how you are feeling right now?
    - Do you feel able to go on with your day?
    - Do you feel safe?

Review

- If participant feels able to continue resume interview / discussion
- If not go to stage 2

Stage 2 Response

- Remove participant from discussion to a quiet area /stop interview
- Encourage participant to contact GP or other health provider, family member or friend OR
- Offer for a member of the research team to do so

Follow up

- Follow up participant with courtesy call (if participant consents) OR
- Encourage participant to call member of the research team if experiences increased distress in the days following an interview / focus group
- Refer to Supervisor of Midwives for further support and guidance if appropriate

Adapted from Haigh and Witham (2010)[41]

Appendix 12: Public Liability insurance

ZURICH
MUNICIPAL

To Whom It May Concern

Our ref: SP/IND 3 June, 2015

Zurich Municipal Customer: The University of Manchester

This is to confirm that The University of Manchester have in force with this Company Public Liability Insurance until the policy expiry on 31 May 2016:

Policy Number: NHE-07CA03-0013

Limit of Indemnity: £ 50,000,000 any one claim

Excess: Nil any one claim

Yours faithfully

Underwriting Services
Zurich Municipal
Farnborough

Zurich Municipal
Zurich House
2 Gladiator Way
Farnborough
Hampshire
GU14 6GB

Telephone 0870 2418050
Direct Phone 01252 387859
Direct Fax 01252 375893
E-mail alison.cliff@uk.zurich.com

Communications will be monitored regularly to improve our service and for security and regulatory purposes

Zurich Municipal is a trading name of Zurich Insurance Group Ltd

A public limited company incorporated in Ireland. Registration No. 13460 Registered Office: Zurich House, Ballsbridge Park, Dublin 4, Ireland.

UK branch registered in England and Wales Registration No. BR7985. UK Branch Head Office: The Zurich Centre, 3000 Parkway, Whiteley, Fareham, Hampshire PO15 7JZ

Authorised by the Central Bank of Ireland and subject to limited regulation by the Financial Conduct Authority. Details about the extent of our regulation by the Financial Conduct Authority are available from us on request.

Appendix 13: Employers' Liability insurance

ZURICH MUNICIPAL

Certificate of Employers' Liability Insurance(a)

(Where required by regulation 5 of the Employers' Liability (Compulsory Insurance) Regulations 2008 (the Regulations), a copy of this certificate must be displayed at all places where you employ persons covered by the policy or an electronic copy of the certificate must be retained and be reasonably accessible to each employee to whom it relates).

Policy No.	NHE-07CA03-0013
1. Name of policyholder	The University of Manchester
2. Date of commencement of insurance policy	01 June 2015
3. Date of expiry of insurance policy	31 May 2016

We hereby certify that subject to paragraph 2:

1. The policy to which this certificate relates satisfies the requirements of the relevant law applicable in Great Britain, Northern Ireland, the Isle of Man, the Island of Jersey, the Island of Guernsey and the Island of Alderney **(b)**

2. **(a)** the minimum amount of cover provided by this policy is no less than £5 million **(c)**

Signed on behalf of Zurich Insurance plc (Authorised Insurer).

Signature

Stephen Lewis

Chief Executive Officer, Zurich Insurance plc (UK Branch)

Zurich Municipal is a trading name of Zurich Insurance plc
A public limited company incorporated in Ireland
Registration No.13460 Registered Office Zurich House, Ballsbridge Park ,Dublin 4 Ireland.
UK branch registered in England and Wales Registration No. . BR 7985
UK Branch Head Office
The Zurich Centre, 3000 Parkway, Whiteley, Fareham, Hampshire PO15 7JZ

Authorised by the Central Bank of Ireland and subject to limited regulation by the Financial Conduct Authority. Details about the extent of our regulation by the Financial Conduct Authority are available from us on request

Notes

(a) Where the employer is a company to which regulation 3(2) of the Regulations applies, the certificate shall state in a prominent place, either that the policy covers the holding company and all its subsidiaries, or that the policy covers the holding company and all its subsidiaries except any specifically excluded by name, or that the policy covers the holding company and only the named subsidiaries.

(b) Specify applicable law as provided for in regulation 4(6) of the Regulations.

(c) See regulation 3(1) of the Regulations and delete whichever of paragraphs 2(a) or 2(b) does not apply. Where 2(b) is applicable, specify the amount of cover provided by the relevant policy

Appendix 14: Professional indemnity insurance

MARSH

Cliffe Crowther
Marsh Ltd
Belvedere
12 Booth Street
Manchester
M2 4AW
+44 (0) 161 954 7317
Fax +44 (0) 161 954 7210
Cliffe.crowther@marsh.com
www.marsh.com

To whom it may concern

29th May 2015

Dear Sirs,

CONFIRMATION OF INSURANCE – The University of Manchester and Subsidiary Companies

As requested by the above client, we are writing to confirm that we act as Insurance Brokers to the client and that we have arranged insurance(s) on its behalf as detailed below:

PROFESSIONAL INDEMNITY INSURANCE

INSURERS	Novae Underwriting Ltd.
POLICY NUMBER	003210MMA15C
PERIOD OF INSURANCE	01 June 2015 to 31st May 2016, both dates inclusive.
LIMIT OF INDEMNITY	GBP10,000,000 any one claim and in the aggregate any one insurance period plus costs and expenses.
DEDUCTIBLE	GBP20,000 each & every claim including costs and expenses

INSURANCE BROKERS CII CHARTERED

Registered in England Number: 1507274, Registered Office:
1 Tower Place West, Tower Place, London EC3R 5BU.
Marsh Ltd is authorised and regulated by the Financial Conduct Authority

MARSH & McLENNAN COMPANIES

MARSH

29th of May 2015

We have placed the insurance which is the subject of this letter after consultation with the client and based upon the client's instructions only. Terms of coverage, including limits and deductibles, are based upon information furnished to us by the client, which information we have not independently verified.

This letter is issued as a matter of information only and confers no right upon you other than those provided by the policy. This letter does not amend, extend or alter the coverage afforded by the policies described herein. Notwithstanding any requirement, term or condition of any contract or other document with respect to which this letter may be issued or pertain, the insurance afforded by the policy (policies) described herein is subject to all terms, conditions, limitations, exclusions and cancellation provisions and may also be subject to warranties. Limits shown may have been reduced by paid claims.

We express no view and assume no liability with respect to the solvency or future ability to pay of any of the insurance companies which have issued the insurance(s).

We assume no obligation to advise yourselves of any developments regarding the insurance(s) subsequent to the date hereof. This letter is given on the condition that you forever waive any liability against us based upon the placement of the insurance(s) and/or the statements made herein with the exception only of wilful default, recklessness or fraud.

This letter may not be reproduced by you or used for any other purpose without our prior written consent.

This letter shall be governed by and shall be construed in accordance with English law.

Yours faithfully

Cliffe Crowther

MARSH & McLENNAN COMPANIES

References

1. Beck S, Wojdyla D, Say L, *et al.* The worldwide incidence of preterm birth: a systematic review of maternal mortality and morbidity. *Bull World Health Organ* 2010;88:31–38. doi:10.2471/BLT.08.062554.
2. Health and Social Care Information Centre. *Hospital Episode Statistics: NHS Maternity statistics — England, 2013–14.* Leeds, HSIC.
3. Saigal S, Doyle LW. An overview of mortality and sequelae of preterm birth from infancy to adulthood. *Lancet* 2008;371:261–9.
4. D'Onofrio BM, Class QA, Rickert ME, *et al*. Preterm birth and mortality and morbidity: a population-based quasi-experimental study. *JAMA Psychiatry* 2013; 70(11):1231-1240. doi:10.1001/jamapsychiatry.2013.2107 .
5. Mangham LJ, Petrou S, Doyle LW, *et al.* The cost of preterm birth throughout childhood in England and Wales. *Pediatrics* 2009;123(2):e312–27. doi: 10.1542/peds.2008-1827.
6. Alfirevic Z, Allen-Coward H, Molina F, *et al.* Targeted therapy for threatened preterm labor based on sonographic measurement of the cervical length: a randomized controlled trial. *Ultrasound Obstet Gynecol* 2007;29:47–50. DOI: 10.1002/uog.3908.
7. Salim R, Garmi G, Nachum Z, *et al.* Nifedipine compared with atosiban for treating preterm labor: a randomized controlled trial. *Obstet Gynecol* 2012;120:1323–31. DOI:10.1097/AOG.0b013e3182755dff
8. Roberts D, Dalziel SR. Antenatal corticosteroids for accelerating fetal lung maturation for women at risk of preterm birth. *Cochrane Database of Systematic Reviews* 2006, Issue 3. Art. No.: CD004454. DOI: 10.1002/14651858.CD004454.pub2.
9. Crowther CA, McKinlay CJD, Middleton P, Harding JE. Repeat doses of prenatal corticosteroids for women at risk of preterm birth for improving neonatal health outcomes.

Cochrane Database of Systematic Reviews 2015, Issue 7. Art. No.: CD003935. DOI: 10.1002/14651858.CD003935.pub4.

10. Doyle LW, Crowther CA, Middleton P, Marret S, Rouse D. Magnesium sulphate for women at risk of preterm birth for neuroprotection of the fetus. *Cochrane Database of Systematic Reviews* 2009, Issue 1. Art. No.: CD004661. DOI:10.1002/14651858.CD004661.pub3.
11. Royal College of Obstetricians and Gynaecologists. *Tocolysis for women in preterm labour: Green-top Guideline No. 1b.* 2011. London: RCOG.
12. Murphy KE, Hannah ME, Willan AR, Hewson SA, Ohlsson A, Kelly EN, Matthews SG, Saigal S, Asztalos E, Ross S, Delisle M-F, Amankwah K, Guselle P, Gafni A, Lee SK, Armson BA, for the MACS Collaborative Group. Multiple courses of antenatal corticosteroids for preterm birth (MACS): a randomised controlled trial. *Lancet* 2008; 372: 2143–51.
13. Norberg H, Stålnacke J, Diaz Heijtz R, Smedler A-C, Nyman M, Forssberg H, Norman M. Antenatal corticosteroids for preterm birth: dose-dependent reduction in birthweight, length and head circumference. *Acta Paediatrica* 2011;100:364–9.
14. Asztalos E, Willan A, Murphy K, Matthews S, Ohlsson A, Saigal S, Armson A, Kelly E, Delisle M-F, Gafni A, Lee S, Sananes R, Rovet J, Guselle P, Amankwah K, for the MACS-5 Collaborative Group. Association between gestational age at birth, antenatal corticosteroids, and outcomes at 5 years: multiple courses of antenatal corticosteroids for preterm birth study at 5 years of age (MACS-5). *BMC Pregnancy and Childbirth* 2014; 14:272.
15. Jacquemyn Y, Zecic A, Van Laere D, Roelens K. The use of intravenous magnesium in non-preeclamptic pregnant women: fetal/neonatal neuroprotection. *Arch Gynecol Obstet* 2015;291:969–975.
16. Gyetvai K, Hannah ME, Ellen D, Hodnett ED, Ohlsson A. Tocolytics for preterm labour: a systematic review. *Obstetrics and Gynecology* 1999;94(5):869–77.
17. Hill WC. Risks and complications of tocolysis. *Clinical Obstetrics and Gynecology* 1995;38(4):725–45.

18. Kent RA, Yazbek M, Heyns T, et al. The support needs of high-risk antenatal patients in prolonged hospitalisation. *Midwifery* 2015;31:164–9.

19. Lasswell SM, Barfield WD, Rochat RW, Blackmon L. Perinatal Regionalization for Very Low-Birth-Weight and Very Preterm Infants: A Meta-analysis. *JAMA.* 2010;304(9):992-1000. doi:10.1001/jama.2010.1226.

20. Marlow N, Bennett C, Drasper ES, Hennessy EM, Morgan AS, Costeloe KL. Perinatal outcomes for extremely preterm babies in relation to place of birth in England: The EPICure 2 study. *Arch Dis Child Fetal Neonatal Ed* 2014;99:F181–88.

21. Towers TV, Bonebrake R, Padilla G, Rumney P. The effect of transport on the rate of severe intraventricular hemorrhage in very low birth weight infants. *Obstetrics and Gynecology* 2000;95(2):291–295.

22. Porcellato L, Masson G, O'Mahony F, Jenkinson S, Vanner T, Cheshire K, Perkins E. 'It's something you have to put up with'— service users' experiences of in utero transfer: a qualitative study. *BJOG* 2015; DOI: 10.1111/1471-0528.13235.

23. Bond PA, Crisp AS, Morgan MEI, Lobb MO, Cooke RWI. Maternal attitudes to transfer before delivery. *Journal of Reproductive and Infant Psychology* 1984;2(1):33–41. DOI: 10.1080/02646838408403447.

24. Wilson AM, MacLean D, Skeoch CH, Jackson L. An evaluation of the financial and emotional impact of *in utero* transfers upon families: a Scotland-wide audit. *Infant* 2010;6(2):38–40.

25. Coster-Schulz MA, Mackey MC. The preterm labour experience: a balancing act. *Clinical Nursing Research* 1998;335–59.

26. Gale C, Hay A, Phillip C, et al. In-utero transfer is too difficult: results from a prospective study. *Early Human Development* 2012;88:147–50.

27. Macintyre-Beon C, Skeoch C, Jackson J, Booth P, Cameron A. *Perinatal Collaborative Transport Study: Final report.* 2008. Glasgow: Scottish Neonatal Transport Service.

28. O'Brien ET, Quenby S, Lavender T. Women's view of high risk pregnancy under threat of preterm birth. *Sexual and Reproductive Health* 2010;1:79–84.

29. Hezelgrave NL, Shennan AH, David AL. Tests to predict imminent delivery in threatened preterm labour. *BMJ* 2015;350:h2183 doi: 10.1136/bmj.h2183.

30. Chandiramani M, Di Renzo GC, Gottschalk E, Helmer H, Henrich W, Hoesli I, Mol B, Norman JE, Robson S, Thornton S, Shennan A. Fetal fibronectin as a predictor of spontaneous preterm birth: a European perspective. *The Journal of Maternal-Fetal & Neonatal Medicine* 2011;24(2):330–336.

31. Deshpande SN, van Asselt ADI, Tomini F, Armstrong N, Allen A, Noake C, et al. Rapid fetal fibronectin testing to predict preterm birth in women with symptoms of premature labour: a systematic review and cost analysis. *Health Technol Assess* 2013;17(40).

32. Radford S, Abbott D, Seed P, Kemp J, Shennan. Quantitative fetal fibronectin for the prediction of preterm birth in symptomatic women. *Arch Dis Child Fetal Neonatal Ed* 2012;97(Supp 1):A1–A125.

33. Harrison MJ, Kushner KE, Benzies K, et al. Women's satisfaction with their involvement in health care decisions during a high-risk pregnancy. *Birth* 2003;30(2):109–15.

34. Hill JL, Campbell MK, Zou GY, et al. Prediction of preterm birth in symptomatic women using decision tree modelling for biomarkers. *Am J Obstet Gynecol* 2008;198:468.e1-468.e9.

35. Gallagher K, Martin J, Keller M, et al. European variation in decision-making and parental involvement during preterm birth. *Arch Dis Child Fetal Neonatal Ed* 2014;99:F245–9.

36. Peerzada JM, Schollin J, Håkansson S. Delivery room decision-making for extremely preterm infants in Sweden. *Pediatrics* 2006;117(6):1988–95.

37. Smith J, Firth J. Qualitative data analysis: the framework approach. *Nurse Researcher* 2011;18(2):52–62.

38. Rabiee F. Focus group interview and data analysis. *Proceedings of the Nutrition Society* 2004;63: 655–660.

39. Furber C. Framework analysis: a method for analysing qualitative data. *African Journal of Midwifery and Women's Health* 2010;4(2):97–100.

40. Sword W. Accounting for presence of self: reflections on doing qualitative research. *Qualitative Health Research* 1999;9(2):270–8.

41. Haigh C, Witham G. *Distress protocol for qualitative data collection.* 2015. [Online] available at: http://www2.mmu.ac.uk/media/mmuacuk/content/documents/rke/Advisory%20Distress%20Protocol.pdf accessed October 2015.
