# Supplementary material for: Study protocol: quantitative fibronectin to help decision-making in women with symptoms of preterm labour (QUIDS) part 2, UK Prospective Cohort Study
Source: BMJ Open. 2018 Apr 19;8(4):e020795. doi: 10.1136/bmjopen-2017-020795 (PMC5914783; doi:10.1136/bmjopen-2017-020795)
Supplement: Author's manuscript [file bmjopen-2017-020795.draft_revisions.pdf]

BMJ Open

BMJ Open is committed to open peer review. As part of this commitment we make the peer review history of every article we publish publicly available.

When an article is published we post the peer reviewers' comments and the authors' responses online. We also post the versions of the paper that were used during peer review. These are the versions that the peer review comments apply to.

The versions of the paper that follow are the versions that were submitted during the peer review process. They are not the versions of record or the final published versions. They should not be cited or distributed as the published version of this manuscript.

BMJ Open is an open access journal and the full, final, typeset and author-corrected version of record of the manuscript is available on our site with no access controls, subscription charges or pay-per-view fees (http://bmjopen.bmj.com).

If you have any questions on BMJ Open's open peer review process please email info.bmjopen@bmj.com

BMJ Open

Study Protocol: Quantitative Fibronectin to help Decision-making in women with Symptoms of Preterm Labour (QUIDS) Part Two- Prospective Cohort Study

Journal:	*BMJ Open*
Manuscript ID	bmjopen-2017-020795
Article Type:	Protocol
Date Submitted by the Author:	24-Nov-2017

Complete List of Authors:	Stock, Sarah; University of Edinburgh MRC Centre for Reproductive Health, Tommy's Centre for Maternal and Fetal Health; University of Western Australia School of Women's and Infant's Health,<br>Wotherspoon, Lisa; University of Edinburgh MRC Centre for Reproductive Health, Tommy's Centre for Maternal and Fetal Health<br>Boyd, Kathleen; University of Glasgow, Health Economics & Health Technology Assessment<br>Morris, R. K.; Univ Birmingham, School of Clinical and Experimental Medicine<br>Dorling, Jon; Queen's Medical Centre, Neonatal Unit<br>Jackson, Lesley; Royal Hospital for Children Glasgow, Neonatal Unit<br>Chandiramani, Manju; Imperial College Healthcare NHS Trust, Queen Charlotte and Chelsea Hospital, Du Cane Road, Shepherds Bush, London, W12 0HS<br>David, Anna; University College London Medical School, Institute for Womens Health<br>Khalil, Asma; St. George's Medical School, University of London<br>Shennan, Andrew; Kings College London, Maternal and Fetal Research Unit<br>Hodgetts Morton, Victoria ; Birmingham Women's Hospital, Metchley Park Road, Edgbaston, ,<br>Lavender, Tina; Manchester University<br>Khan, Khalid; Queen Mary, University of London, Centre for Primary Care and Public Health<br>Harper-Clarke, Susan; PPI Representative<br>Mol, Ben; University of Adelade, The Robinson Institute, School of Paediatrics and Reproductive Health<br>Riley, Richard; Keele University<br>Norrie, John; Edinburgh Clinical Trials Unit, University of Edinburgh No. 9, Bioquarter<br>Norman, Jane; University of Edinburgh

Keywords:	Pregnancy, Preterm Birth, Fetal Fibronectin, Cervix, Diagnostic Test

SCHOLARONE™
Manuscripts

Study Protocol: Quantitative Fibronectin to help Decision-making in women with Symptoms of Preterm Labour (QUIDS) Part Two- Prospective Cohort Study

Sarah J Stock*[1,2] Sarah.Stock@ed.ac.uk
Lisa Wotherspoon[1] Lisa.Wotherspoon@ed.ac.uk
Kathleen Boyd[3] Kathleen.Boyd@glasgow.ac.uk
Rachel K Morris[4] r.k.morris@bham.ac.uk
Jon Dorling[5] Jon.Dorling@nottingham.ac.uk
Lesley Jackson[6] Lesley.Jackson@ggc.scot.nhs.uk
Manju Chandiramani[7] m.chandiramani@imperial.ac.uk
Anna David[8] a.david@ucl.ac.uk
Asma Khalil[9] akhalil@sgul.ac.uk
Andrew Shennan[10] andrew.shennan@kcl.ac.uk
Victoria Hodgetts-Morton[4] v.a.h.morton@bham.ac.uk
Tina Lavender[11] Tina.Lavender@manchester.ac.uk
Khalid Khan[12] k.s.khan@qmul.ac.uk
Susan Harper-Clarke[13] su_clarke@hotmail.com
Ben Mol[14] ben.mol@adelaide.edu.au
Richard D. Riley[15] r.riley@keele.ac.uk
John Norrie[16] J.Norrie@ed.ac.uk
Jane Norman[1] Jane.Norman@ed.ac.uk

1. Tommy's Centre for Maternal and Fetal Health, University of Edinburgh MRC Centre for Reproductive Health, Queen's Medical Research Institute, Edinburgh, UK. 2. School of Women's and Infants' Health, University of Western Australia, Crawley WA 6009, AUS. 3. Institute of Health & Wellbeing Health Economics & Health Technology Assessment, University of Glasgow, 1 Lilybank Gardens, Glasgow, UK. 4. School of Clinical and Experimental Medicine, University of Birmingham, Birmingham, UK. 5. Queen's Medical Centre, Neonatal Unit, Derby Road,

For peer review only

Nottingham, UK. 6. Royal Hospital for Children Glasgow, Neonatal Unit Glasgow, UK. 7. Imperial College Healthcare NHS Trust, Queen Charlotte and Chelsea Hospital, Du Cane Road, Shepherds Bush, London, UK. 8. University College London Medical School, Institute for Women's Health Health, 86-96 Chenies Mews, University College London Medical School London, UK. 9. St. George's Medical School, University of London, London, UK. 10. Kings College London, Maternal and Fetal Research Unit Maternal and Fetal Research Unit 10th Floor, North Wing London, UK. 11. University of Manchester School of Nursing, Midwifery & Social Work, Jean McFarlane Building, University Place, Oxford Road Manchester, UK. 12. Queen Mary, University of London, Centre for Primary Care and Public Health London, UK. 13. Patient and Public Involvement Representative. No affiliation. 14. University of Adelaide, The Robinson Institute, School of Paediatrics and Reproductive Health Adelaide, SA, AUS. 15. Research Institute for Primary Care and Health Sciences, Keele University, Keele, UK. 16. Edinburgh Clinical Trials Unit, University of Edinburgh No. 9, Bioquarter Edinburgh, UK.

*Corresponding Author: Dr Sarah J Stock Senior Clinical Lecturer Maternal and Fetal Medicine, MRC Centre for Reproductive Health, Queen's Medical Research Institute, 47 Little France Crescent, Edinburgh, EH16 4TJ. Tel: 0131 242 6449 Email: sarah.stock@ed.ac.uk

Word Count: 4,928

Key Words: Pregnancy; Preterm Birth; Fetal Fibronectin; Cervix; Diagnostic Test.

For peer review only

ABSTRACT

Introduction: The aim of the QUIDS study is to develop a decision support tool for the management of women with symptoms and signs of preterm labour, based on a validated prognostic model using quantitative fetal Fibronectin (fFN) concentration, in combination with clinical risk factors.

Methods and analysis: The study will evaluate the Rapid fFN 10Q System (Hologic, Malborough, MA) which quantifies fFN in a vaginal swab. In QUIDS Part 2 we will perform a prospective cohort study in at least eight UK consultant-led maternity units, in women with symptoms of preterm labour at 22+0 to 34+6 weeks gestation to externally validate a prognostic model developed in QUIDS Part 1. The effects of quantitative fFN on anxiety will be assessed, and acceptability of the test and prognostic model will be evaluated in a subgroup of women and clinicians (n=30). The sample size is 1600 women (with estimated 96-192 events of preterm delivery within 7 days of testing). Clinicians will be informed of the qualitative fFN result (positive/negative) but be blinded to quantitative fFN result. Research midwives will collect outcome data from the maternal and neonatal clinical records. The final validated prognostic model will be presented as a mobile or web-based application.

Ethics and dissemination: The study is funded by the National Institute of Healthcare Research Health Technology Assessment (HTA 14/32/01). It has been approved by the West of Scotland Research Ethics Committee (16/WS/0068).

Registration details: The study has been registered with ISRCTN Registry (ISRCTN 41598423) and NIHR Portfolio (CPMS: 31277)

Version: Protocol Version 2, Date 1st November 2016

STRENGTHS AND LIMITATIONS OF THIS STUDY

Strengths

- Validation of a prognostic model in a separate prospective cohort study

For peer review only

- Health Economic Analysis to determine cost effectiveness from NHS perspective

Limitations

- Not a randomized control trial to test effectiveness of the model on improved patient outcomes

HOW PATIENTS ARE INVOLVED IN THIS STUDY

Patient representatives were consulted during the protocol development and have been invited to join the Project Management Group and the Trial Steering Committee. Prior to commencing QUIDS, we performed a qualitative study to determine the decisional needs of pregnant women with signs and symptoms of preterm labour, their partners and their caregivers. This is described in the separate protocol “QUIDS Qualitative” (Supplementary Material). The end product of QUIDS will be a decision support aid to help clinicians, women and their partners decide on management of threatened preterm labour, based on the results of the quantitative fFN. In QUIDS Qualitative women and clinicians indicated that they would prefer this to be on web based or mobile app based format, presenting the risk of preterm birth within seven days of testing.

For peer review only

INTRODUCTION

The overall aim of the QUIDS study is to develop a decision support tool for the management of women with symptoms and signs of preterm labour, based on a validated prognostic model using quantitative fFN testing. The study has been conceptually divided into two parts. In this, the protocol for QUIDS Part Two, we detail the protocol for a prospective cohort study. This will externally validate a prognostic model developed in QUIDS Part One.[1] More detailed background about the diagnosis of preterm labour and background to the study is provided in the introduction of QUIDS Protocol Part One.[1]

Fetal Fibronectin (fFN) is a biochemical test of preterm labour which has potential to help improve diagnosis of impending preterm delivery.[2] Much of the evidence about fFN to date relates to the qualitative fFN test, which provides a positive or negative result on the basis of a single threshold of 50ng/ml.[2,3] This test has been largely replaced with the Rapid fFN 10Q System, which provides a concentration of fFN (quantitative fFN) and may be a more useful predictor of preterm delivery. fFN is now only available with a quantitative analyser in the UK, but there is no consensus as to which women to use the test in, or how to interpret the results.

The QUIDS study will address this evidence gap by providing evidence about the potential value of the quantitative fFN test, along with guidance about how to interpret results. Here we detail the protocol for external validation of a prognostic model developed in QUIDS Part One.[1]

METHODS AND ANALYSIS

Aims and Methodologies

For peer review only

The aim of the QUIDS study is to develop a decision support tool for the management of women with symptoms and signs of preterm labour, based on a validated prognostic model using quantitative fFN testing.

The study protocol has been divided into two parts (see flow chart Figure 1). The protocols for Parts One and Two are reported in separate manuscripts.

In QUIDS Protocol Part One we have described how we will perform (i) an Individual Participant Data (IPD) meta-analysis, and (ii) and Economic Analysis. The protocol details how we will develop and internally validate a prognostic model using quantitative fFN and other risk (prognostic) factors and to evaluate the added value of quantitative fFN toward this prognostic model performance. We will also provide an economic rationale for the prognostic model and analyze its cost-effectiveness from the perspective of the NHS.

In this, the QUIDS Protocol Part Two, we will detail the prospective cohort study to externally validate and, if necessary, refine the prognostic model. This will be performed in at least eight UK hospitals with different settings (rural/urban) and different levels of neonatal care facilities. In addition, acceptability of quantitative fFN testing, and effects on maternal anxiety will be performed. We will assess the potential cost-effectiveness of the final prognostic model/decision support tool. This additional analysis will allow us to model the full costs and effect impacts of the different prognostic model and compare these in a cost-effectiveness analysis to provide an evidence-based economic rationale for implementing the diagnostic tool in the NHS.

Endpoints

For peer review only - http://bmjopen.bmj.com/site/about/guidelines.xhtml

The primary endpoint of the prognostic model is spontaneous preterm delivery within seven days of qfFN test, in women less than 36 weeks' gestation. This was influenced by the preceding QUIDS Qualitative Study, which included focus group consultation to determine the decisional needs of women, their partners and clinicians (Supplementary Material). It is also a recognised clinically important endpoint, as antenatal steroids (which significantly reduce morbidity and mortality in preterm babies[4]) are most effective if delivery occurs within seven days of administration.

A secondary endpoint suggested by QUIDS Qualitative Study (Supplementary Material) consultation, was delivery within 48 hours of qfFN test. This analysis will be performed if feasible to do so within the constraints of the data available for model development and validation.[1]

Health technologies being assessed

The trial will evaluate the Rapid fFN 10Q System (Hologic, Malboroughm MA). This provides a concentration of fFN (ng/ml or INVALID) in a vaginal swab sample in 10 minutes. It is now the only commercially available fFN test system, and replaces the TLiQ rapid analyser system, which provided a qualitative fFN result (POSITIVE or NEGATIVE) based on a threshold of 50ng/ml. The Rapid fFN 10Q system is a point of care test, which clinical staff can easily perform. All reagents for fFN testing can be stored at room temperature and specimen collection kits, reagents, cassettes and the 10Q analyzer can be kept in clinical areas where women with symptoms of preterm labour are assessed so they can be conveniently accessed.

Vaginal swab samples are analysed by lateral flow; solid-phase immunochromatographic assay (the Rapid fFN Cassette), and interpreted in the 10Q Rapid analyser. 200 μL of the sample is pipetted into the sample application well of

For peer review only

the Rapid fFN Cassette using a polypropylene or polyethylene pipette. The sample will then flow from an absorbent pad across a nitrocellulose membrane via capillary action through a reaction zone containing murine monoclonal anti-fetal fibronectin antibody conjugated to blue microspheres (conjugate). The conjugate, embedded in the membrane, will be mobilized by the flow of the sample. The sample will then flow through a zone containing goat polyclonal antihuman fibronectin antibody that captures the fibronectin-conjugate complexes. The remaining sample will flow through a zone containing goat polyclonal anti-mouse IgG antibody that captures unbound conjugate, resulting in a control line. After 10 minutes of reaction time, the intensities of the test line and control line are interpreted with the 10Q Rapid analyser and a printed result provided as a concentration in ng/ml (0->500ng/ml) or INVALID. The result is invalid if the test does not meet internal quality controls that are performed automatically with every test. In the event of an invalid result, the test can be repeated with any remaining clinical specimen. A quality control can be performed by a reusable Rapid fFN 10Q QCette® QC Device, which verifies that the analyser performance is within specification.

Target population

The target population is pregnant women attending hospital with signs and symptoms of preterm labour.

Validation And Refinement Of Prognostic Model

Population

The prospective cohort study will include women with signs and symptoms of preterm labour at 22^{+0} to 34^{+6} weeks gestation in whom admission, transfer or treatment is being considered. These will be recruited from at least eight sites with a mix of rural/urban settings, and have different levels of neonatal care facilities, over 12 months.

For peer review only

Eligibility Criteria

The following inclusion criteria will apply at screening assessment (all apply):

- Women who are 22^{+0} to 34^{+6} weeks (or earlier gestation if the fetus is considered potentially viable).
- Women showing signs and symptoms of pre-term labour which may include any or all of back pain, abdominal cramping, abdominal pain, light vaginal bleeding, vaginal pressure, uterine tightenings or contractions.
- Women where hospital admission, interhospital transfer or treatment (antenatal steroids, tocolysis or magnesium sulphate) is being considered due to signs of pre-term labour.
- Women aged 16 years or above.

The broad inclusion criteria reflect current clinical practice and enable the generalisability of the results of the trial for routine clinical care. We will include women who re-attend seven days or more after initial recruitment with signs and symptoms of preterm labour and also women who remain symptomatic but undelivered seven days later in whom repeat testing by the clinician is deemed to be appropriate. This will be in line with manufacturer's recommendation for fFN testing.

The following inclusion criteria will apply on speculum examination:

- Cervical dilation ≤ 3cm
- Intact membranes
- No significant vaginal bleeding, as judged by the clinician.
- Once it has been established that the women meets the above criteria, on speculum examination, the fFN swab can be taken.

Participants that sign the consent but are not eligible upon examination to have an fFN swab taken will still be enrolled and have outcome data collected.

For peer review only

The following exclusion criteria will apply:

- Contraindication to vaginal examination (e.g. placenta praevia).
- Higher order multiple pregnancy (triplets or more).
- Moderate or severe vaginal bleeding.
- Cervical dilatation greater than 3cm.
- Confirmed rupture of membranes.
- Sexual intercourse, vaginal examination or transvaginal ultrasound in the preceding 24 hours factors may invalidate results. These women will be initially excluded from the study, but can be included if still symptomatic after 24 hours, when fFN accuracy will be restored.

Co-Enrolment

This trial involves validating a decision support tool relating to a test that is currently commonly used in clinical practice. As such, there are no additional interventions. Co-enrolment in other non-interventional trials will be allowed. Co-enrolment in trials of tocolytic treatments or other management strategies that may influence timing of delivery as a primary outcome will not be allowed. Participation in QUIDs would not preclude babies being subsequently involved in interventional trials. Co-enrolment will be recorded in the electronic case report form (eCRF).

Setting

The prospective cohort study will take place in at least eight consultant-led obstetric units in the UK. More than 93% of pregnant women in the UK deliver in consultant-led units.[5,6] The vast majority of women with symptoms of preterm labour will present to a consultant-led unit for assessment, either directly or following advice from their community midwife or General Practitioner.

For peer review only

The study will not include any community maternity units (staffed by midwives, with or without involvement of non-obstetric medical staff), which cover a small proportion of women, mainly in remote and rural areas. In the Perinatal Collaborative Transport Study (CoTS study) of perinatal transfers in Scotland,[7] which involved 52,727 births, only 69 (0.13%) women were transferred to a consultant-led obstetric unit from community maternity units, and only a proportion of these were for suspected preterm labour. The small number of women cared for in community maternity units means their inclusion would not be an efficient use of study resources.

Given that management of women with symptoms of preterm labour and inter-hospital transfer patterns are likely to vary depending on level of available neonatal care and distance to transfer, we will include a mixture of hospitals with different levels of neonatal care facilities in both rural and urban settings. We will include units with Special Care Units (providing special care for their own local population), Local Neonatal Units (providing special care and high dependency care and a restricted volume of intensive care) and Neonatal Intensive Care Units (larger intensive care units providing the whole range of medical, and sometimes surgical neonatal care for their local population and for babies and their families referred from the neonatal network in which they are based, and other networks when necessary). The hospitals will be chosen from different geographical settings (rural/urban) and from different regions of the UK.

If additional units wish to participate in the study we will consider including them, to increase recruitment rates. The UK Reproductive Health and Childbirth specialty group (clinical study group) have contributed to the study protocol and support the proposed trial.

For peer review only

Participant Selection And Enrolment

Women with signs and symptoms of preterm labour will be identified on presentation to obstetric services. A member of clinical staff, usually the doctor or midwife assessing the woman, will identify potentially eligible participants, provide a participant information leaflet and invite consent. A suitably trained member of clinical staff (doctor or midwife) or research team will consent participants.

Posters and leaflets will be situated in antenatal areas of participating hospitals to alert women that the study is taking place, and women will be allowed as much time as possible to consider participation without unduly delaying further clinical assessment. Participants will receive adequate oral and written information and appropriate participant information and informed consent forms will be provided.

Screening For Eligibility

The clinical likelihood of preterm delivery is usually evaluated by history and examination, which includes abdominal palpation, to assess strength and frequency of uterine contractions. If preterm labour is suspected, a vaginal speculum examination is performed where the cervix is inspected for dilatation, and evidence of vaginal bleeding and membrane rupture assessed. Swabs for fFN are usually taken at this point. Potential participants in the QUIDS study will be identified after the initial assessment and provided with information about the study. A combined 'Screening and Consent Form' will be used as a self-screening tool for potentially eligible participants. Informed consent will take place before speculum examination and the fFN swab has been taken. This approach means that samples are collected at routine speculum examination, as they would be if fFN is implemented in clinical practice, and participants avoid an additional vaginal examination.

Ineligible And Non-Recruited Participants

For peer review only - http://bmjopen.bmj.com/site/about/guidelines.xhtml

Certain exclusion criteria can only be assessed at speculum examination (for example vaginal bleeding or evidence of ruptured membranes), so a proportion of women will not be eligible for fFN testing after consent is given. These women will still be enrolled and delivery outcomes collected. The decision whether to use this data for analysis will be the decision of the Chief Investigator and Statisticians.

Withdrawal Of Study Participants

Women will be able to withdraw consent for us of their data at any time until the end of the study.

Study Assessments (See Table 1)

Eligibility Assessment (Screening And Recruitment)

Women presenting with signs and symptoms of pre-term labour will be identified on presentation to obstetric services. The doctor or midwife assessing the woman will identify potentially eligible participants and provide an invitation letter and short information leaflet.

After the woman has had the opportunity to consider whether she would like to participate, she will be asked to complete the Screening and Consent Form. The clinician will then decide whether the fFN test can be carried out. If the test can be carried out (according to manufacturer’s guidelines), then the participant will be fully enrolled and that their delivery outcomes will still be collected.

If the woman declines to participate and she is willing to provide a reason for this, the reason given will be entered on to an anonymous log. Baseline demographics will be collected on consenting women, together with height and weight, information on medical history, obstetric history, estimated date of delivery together with the signs and symptoms they are presenting with.

For peer review only - http://bmjopen.bmj.com/site/about/guidelines.xhtml

The original consent form will be stored in the Investigator Site File (ISF) file, a copy is given to the woman, a copy added to the medical notes and a copy sent to the Trial Office.

After providing consent, the participant will be asked to complete a short State Trait Anxiety Inventory (STAI) questionnaire and complete a contact details form. They will also be issued with a letter thanking them for taking part in the trial and giving details of the second questionnaire to be completed.

Sample Collection

Samples for analysis will be taken with a fFN specimen collection kit, which consists of a sterile polyester tipped swab and a specimen transport tube containing 1 ml extraction buffer (an aqueous solution containing protease inhibitors and protein preservatives including aprotinin, bovine serum albumin, and sodium azide). During speculum examination the sterile swab will be lightly rotated across the posterior fornix of the vagina for ten seconds to absorb vaginal secretions. Samples should be taken before any other swabs (e.g. for microbiology) or cervical manipulation and the speculum lubricated with normal saline as other lubricants may interfere with the antibody-antigen reaction of the test. Following specimen collection the swab should be removed, immersed in extraction buffer, the shaft of the swab snapped off, and the transport tube sealed.

Before analysis samples are gently mixed and as much liquid as possible expressed from the swab by rolling the tip against the inside of the tube.

Initial fFN test

For peer review only

The sample taken will be run at a near bedside Hologic Rapid fFN 10Q analyser, specially adapted for the QUIDS study. As fFN (or other similar biochemical tests of preterm labour) are part of standard care, it would be unethical to blind clinicians from the qualitative fFN result. The analyser will thus reveal a qualitative fFN result (positive/negative/invalid) for clinicians to base clinical decision-making on, according to local protocols. The quantitative fFN result however, will be stored as a three-letter code, blinding caregivers from the result. Samples will be run as per manufacturers instructions (described above in the section "Health technologies being assessed").

Repeat fFN Tests

If there is clinical indication for further fFN tests (eg because of ongoing symptoms of preterm labour after seven days), the results will also be recorded.

Labour/Delivery/ Neonatal Assessments

Admission for delivery will not be a formal study visit but data will be collected using information recorded in the participant's notes. Delivery data will be collected on the maternal outcomes of delivery, including method of delivery, indication for delivery method, onset of labour, date and gestation of delivery and blood loss.

Questionnaires

All participants who are eligible to participate will be asked to complete a STAI questionnaire before the speculum examination. The same questionnaire will be repeated 24-48 hours post examination. The second questionnaire will be provided on paper with a pre-paid envelope to be returned by post to the Trial Office. If not returned by post, the Trial Office may try to contact the participant (with the contact details provided), to complete the questionnaire over the phone.

For peer review only

	Attendance with signs and symptoms preterm labour			
**Visit**	**Screening and Recruitment**	**24-48h**	**1-6 months**	**DELIVERY**
Inclusion/Exclusion Criteria	⊙			
Participant Information Sheet	⊙			
Consent Form	⊙			
Demographics	⊙			
Obstetric History	⊙			
Symptoms and Signs	⊙			
Quantitative ffN	⊙			
Cervical length scan (if available)	⊙			
State Trait Anxiety Inventory Questionnaire	⊙	⊙		
Delivery details				⊙
Neonatal outcomes				⊙
Qualitative Acceptability Questionnaires (subgroup n=30)			⊙	

Table 1: QUIDS Study Assessments

For peer review only - http://bmjopen.bmj.com/site/about/guidelines.xhtml

Safety and Quality Assessments

The Hologic Rapid fFN 10Q analyzer has integrated quality control measures, and we will keep records of these as well as any additional staff training that occurs after the study starts. It is recommended that a daily pre-calibrated reusable quality control cassette be inserted and analysed every 24 hours to verify that the analyser performance is within specification. A daily quality control (QC) should be performed if one has not been done in the preceding 24 hours before a patient test is to be done. Logs of results are stored on the machine and can be downloaded, and we will also ask the participating sites to keep a monthly paper log of QC tests done. Each patient test has an internal quality control, with a procedural control line that verifies the threshold level of signal by the instrument. Sample flow detection ensures the sample travels across the cassette properly, and confirms absence of conjugate aggregation. We believe that these measures will help ensure the validity of results. However, to provide further evidence of integrity and comparability of results from each site we will request that all participating sites enrol in the Wales External Quality Assurance Scheme (WEQAS) Point of Care Quality Assurance Scheme. WEQAS will provide a sample for analysis to each site bimonthly, and provide reports on analyser performance and variability.[8]

Data Collection

Data For Prognostic Model Validation and Update of Health Economic Model

We will collect data on all of the candidate predictors considered for inclusion in the prognostic model developed in the IPD meta-analysis. Outcome data will include gestational age at delivery, date and time of delivery, administration of treatments for preterm labour (steroids, antibiotics, tocolysis, magnesium sulphate) duration hospital admission, hospital transfer, onset of labour (preterm prelabour rupture of membranes; idiopathic preterm birth; medically indicated preterm birth [and indication]), place of delivery (base hospital, other hospital, outwith hospital), mode of

For peer review only - http://bmjopen.bmj.com/site/about/guidelines.xhtml

delivery, neonatal admission, neonatal complications, perinatal mortality, congenital anomaly, sex and birthweight.

Screening data and data about quantitative fFN testing will be collected on paper based CRFs and research midwives will input these into the web based electronic database. Clinical outcome data will be collected from the medical records.

Maternal Acceptability and Anxiety

Maternal anxiety will be measured pre and post-test (24-48h) using the validated State Trait Anxiety Inventory (STAI) questionnaire. Acceptability of fFN testing and the decision support will be assessed using follow up interviews (face to face or telephone, according to maternal preference) which will be conducted with a sub-group of participants (n=30) purposively sampled and stratified according to geographical location, outcome (preterm labour or not) and anxiety scores. Acceptability will also be assessed in a cohort of clinicians (n=30).

Statistics and Sample Size Calculation

Guidance for external validation suggests at least ten events (preterm delivery within seven days of test) are required for each covariate included in a prognostic model.[9,10] Data from the cohorts included in our IPD meta-analysis suggests an event rate of between 6 and 12%.[1] Based on these estimates a sample size of 1,600 will provide 96 and 192 events (preterm delivery within 7 days).

A UK study has shown that 8.9% of pregnant women present with symptoms of preterm labour and are eligible for quantitative fFN[11] and we anticipate 50% recruitment rate is achievable, thus overall 4.5% of maternities could be recruited. We will initially include eight units in the cohort study with a combined delivery rate of approximately 36,000 per annum. We anticipate that we will achieve target recruitment within 12 months (1 year * 36,000 * 0.089 * 0.5 = 1,602). If however, the

For peer review only - http://bmjopen.bmj.com/site/about/guidelines.xhtml

recruitment rate or event rate is lower than predicted, we will increase the number of sites included in the study and/or the recruitment period, to ensure that a minimum of 60 events (preterm delivery within 7 days of test) are achieved, allowing for external validation of at least six covariates in our model.

It is possible that the IPD meta-analysis will find there is potential added value of combining quantitative fFN testing with cervical length measurement.[12,13] As cervical length measurement has significant resource requirement (estimated NHS cost £68.16 per test) and lack of out of hours provision further limits availability in many NHS hospitals, we think it is very unlikely that cervical length scanning will improve performance of the prognostic model to such a degree as to make it cost effective. We will assess the incremental costs and effects of cervical length measurement in the proposed health economic model performed in parallel with the IPD meta-analysis, and will feed into design considerations during the first iteration of the prognostic model.

If inclusion of cervical length ultrasound is found to be potentially cost-effective, we will assess the feasibility of including it in the prospective cohort study. We anticipate that including cervical length measurement in the prospective cohort study would be extremely difficult in the current NHS setting as the majority of units do not have 24 hour availability of transvaginal ultrasound and/or trained personnel to perform scans. Inclusion of cervical length would also likely decrease recruitment rate (due to need for additional transvaginal ultrasound examination) and require significant additional resources.

Analysis

Validation Of Prognostic Model

The prognostic model developed in the IPD will be externally validated using data collected in the prospective cohort data, using the measures of discrimination and

For peer review only

calibration described in QUIDS Protocol Part One,[1] including R^2, C statistic, calibration slope, calibration-in-the-large, and calibration plots of observed versus predicted risks across deciles (with Loess smoother). The average performance of the model will be summarised across the centers in the cohort study. Between-center heterogeneity in performance will also be summarised, and reduced (if necessary) by recalibration techniques regarding the strategy for the choice of baseline risk (intercept). That is, the predictor effects will not be modified from the IPD meta-analysis model, but the intercept may need to be tailored to improve validation in UK centers (e.g. for rural settings). Based on the findings, a final model and its implementation strategy will then be recommended for use.

Economic Analysis

The economic model will be refined, integrated and updated with data from the prospective study cohort, so as the most up to date and validated evidence is used to inform a cost-effectiveness decision. Such an iterative approach to economic evaluation is now well established.[14,15] The care pathway following diagnosis will be included in the economic analysis, using data from the cohort study such as the diagnostic test accuracy data, resource use data (i.e. steroid use, other medications, time in hospital, hospital transfer) and secondary outcome data (i.e., treatment of side-effects, morbidity, mortality) so as to capture the full costs and effect impacts (quality of life, morbidity and mortality) for both the mother and baby. Resource use data will be combined with unit cost information from the British National Formulary[16] and NHS reference costs.[17,18] Outcomes will be reported as the incremental cost per correct diagnosis, and incremental cost per Quality Adjusted Life Year (QALY) gained of the qfFN prognostic model compared to current practice (no qualitative fFN model). The analysis will adhere to the NICE reference case and the recommended guidelines for decision modeling and reporting of economic analyses.[18] Probabilistic sensitivity analysis will be undertaken to explore how uncertainty in the model inputs impact on the cost-effectiveness outcome.[19]

For peer review only

Acceptability of fFN Testing and Effects on Anxiety

Maternal anxiety will be measured before and after quantitative fFN testing using the validated STAI. The STAI Form Y is a widely used tool for measuring both temporary "state anxiety" and the more general, long-standing "trait anxiety". The STAI is designed for the self-reported assessment of the intensity of feelings of apprehension, tension, nervousness, and worry. STAI-Anxiety scores increase in response to physical danger and psychological stress, making it highly appropriate for this study. The use of STAI in pregnancy studies is discussed by Hundley, et al and we will interpret the results accordingly.[20]

The questionnaire will be administered prior to fFN testing (baseline) and 24-48 hours after the test, to assess early reactions to the test and any acute anxiety prompted by the result of the test. We will also be able to assess any differences in those presented with a high risk or low risk result. Although it might be interesting to assess anxiety again in the latter stages of pregnancy, it is likely that, in this population, many pregnancies will not reach full term. Thus we believe our strategy of repeat questionnaire administration will allow measurement of longer term anxiety induced or alleviated by the test, whilst minimising bias due to preterm or term delivery itself or loss to follow up.

Follow up interviews will be performed with a sub-group of participants (n=30) to enable deeper exploration of women’s views regarding fFN testing, to gain insight into the rationale for responses given in the questionnaires. Interviews will be conducted following confirmation of pregnancy status. Acceptability of the prognostic model will also be assessed with women and a group of clinicians. All interviews will be audio recorded with consent, and field notes taken to ensure an audit trail.

Decision Support

For peer review only - http://bmjopen.bmj.com/site/about/guidelines.xhtml

We will develop a decision support tool in accordance with the guidelines produced by the International Patient Decision Aid Standards (IPDAS) Collaboration.[21] Scoping of decisional requirements and how data should be presented was performed during focus group consultation as part of QUIDS Qualitative (Supplementary Material). A prototype decision support tool incorporating the initial prognostic model developed as part of the IPD-meta-analysis, will be tested with women and clinicians, as part of the acceptability studies described above. A final version will be updated with the validated (and, if necessary revised) prognostic model generated from the prospective cohort study. The multidisciplinary trial steering committee will oversee the development process, and decide how material is selected for inclusion.

ETHICS AND DISSEMINATION

Trial Management And Oversight Arrangements

Project Management Group

The trial will be coordinated by a Project Management Group (PMG), consisting of the grant holders (Chief Investigator and Co-applicants), the trial manager, representatives from the Study Office and CHaRT (the supporting CTU), plus service user representatives (PAG). The PMG will meet approximately every four months by teleconference or face to face.

The Trial Manager based in Edinburgh will oversee the study and will be accountable to the Chief Investigator. The Trial Manager supported by the trial administrator(s) will take responsibility for the day-to-day transaction of study activities. They will be supported by the CTU at CHaRT to provide expertise and guidance. The Trial Manager will be responsible for checking the CRFs for completeness, plausibility and consistency. Any queries will be resolved by the Investigator or delegated member of the trial team.

For peer review only

A Delegation Log will be prepared for each site, detailing the responsibilities of each member of staff working on the trial.

Trial Steering Committee and Data Monitoring Committee

A combined Trial Steering Committee and Data Monitoring Committee (TSC/DMC) will oversee the conduct and progress of the trial. The terms of reference of the Committee will be developed separately. Members of the TSC/DMC will consist of experts and two patient representatives.

Good Clinical Practice

The study will be conducted in accordance with the principles of Good Clinical Practice (GCP). A favorable ethical opinion has been obtained from the appropriate REC (reference 16/WS/0068) and local R&D approval will be obtained prior to commencement of the study at each site.

Dissemination

On completion of the study, the study data will be analysed and tabulated, and a clinical study report will be prepared in accordance with GCP guidelines. Results will be communicated to the academic community via the scientific literature, attendance at conferences and invited presentations. Summaries of results will also be made available to investigators for dissemination within clinics. Social media will be used to signpost publications and conference presentations and highlight important findings. Twitter and Facebook will be used to disseminate findings to professional organizations, charities, stakeholders and the public. Communication to the general public will further be facilitated by our close links with charities such as Tommy's.[22]

We anticipate that the decision support will be made available as web based application that will be made freely available so clinicians can access it easily and it can be readily translatable into UK practice. If it is found to be effective in ruling out

For peer review only

preterm delivery, it is likely that it will decrease unnecessary costly, and potentially harmful treatments in women who have symptoms suggestive of preterm labour but do not deliver early.

PEER REVIEW

The study was extensively peer reviewed as part of the process of gaining grant funding from the NIHR HTA (14/32/01).

FUNDING

This project was funded by the National Institute of Healthcare Research Health Technology and Assessment (Reference 14/32/01). The views expressed are those of the authors and not necessarily those of the NHS, the NIHR or the Department of Health.

CONTRIBUTIONS TO AUTHORSHIP

SJS, KB, RKM, JD, LJ, MC, AD, AK, AS, VHM, TL, KK, SHC, BM, RDR, JN and JEN developed the protocol. SJS, LW, RDR, KB, TL and JN drafted the protocol. RKM, JD, LJ, MC, AD, AK, AS, VHM, KK, SHC, BM and JEN reviewed and commented on the protocol.

COMPETING INTERESTS

SJS and JEN work at the University of Edinburgh, who received £1000 sponsorship from Hologic to support a meeting (The Society of Reproductive Investigation and MRC Centre for Reproductive Health Scientific Symposium on Targeting Inflammation to Improve Reproductive Health across the Lifecourse – August 2017). AS has in the past (over last five years; not in the last three years) received funding for expenses related to advisory board and internal staff education from Hologic.

For peer review only

MC received sponsorship from Hologic to organise an educational teaching focusing on prediction of Preterm Birth at the 2017 annual meeting of the British Maternal and Fetal Medicine Society.

Hologic, the makers of fFN have provided analysers and technical support for their use to sites participating in the QUIDS prospective cohort study. They have no access to the data, or other involvement in the conduct, data analysis, interpretation of results or decision to publish the results of the study.

REFERENCES

1. Stock SJ, Wotherspoon L, Boyd K et al. Study Protocol: Quantitative Fibronectin to help Decision-making in women with Symptoms of Preterm Labour (QUIDS) Part One- Individual Patient Data Meta-analysis and Health Economic Analysis. *Submitted to BMJ Open alongside this manuscript(see supplementary information)*

2. Honest H, Forbes CA, Duree KH, et al. Screening to prevent spontaneous preterm birth: systematic reviews of accuracy and effectiveness literature with economic modelling. Health Technol Assess 2009;13(43):1-627

3. Deshpande SN, van Asselt AD, Tomini F, et al. Rapid fetal fibronectin testing to predict preterm birth in women with symptoms of premature labour: a systematic review and cost analysis. Health Technol Assess 2013;17(40):1-138

4. Roberts D, Brown J, Medley N, Dalziel SR. Antenatal corticosteroids for accelerating fetal lung maturation for women at risk of preterm birth. Cochrane Database Syst Rev 2017;3:CD004454

5. Office for National Statistics (2013). ONS gestation-Specific Infant Mortality in England and Wales 2011. Cardiff ONS, 2013. Also available at: https://www.ons.gov.uk/peoplepopulationandcommunity/healthandsocialcare/

For peer review only

causesofdeath/bulletins/pregnancyandethnicfactorsinfluencingbirthsandinfant mortality/2013-10-10 (accessed 23rd November 2017).

6. Information Services Division (2013). "Births in Scottish hospitals 2012". Edinburgh National Services Scotland, 2013. Also available at: http://www.isdscotland.org/Health-Topics/Maternity-and-Births/Publications/2013-08-27/2013-08-27-Births-Report.pdf?34511965514 (accessed 23rd November 2017).

7. Macintyre-Beon C, Skeoch C, Jackson L, Booth P, Cameron A. Scottish Perinatal Collaborative Transport Study. NHS Quality Improvment Scotland 2008

8. http://www.weqas.com/services/poct-eqa/ (accessed 23rd November 2017).

9. Vergouwe Y, Steyerberg EW, Eijkemans MJ, Habbema JD. Substantial effective sample sizes were required for external validation studies of predictive logistic regression models. J Clin Epidemiol 2005;58(5):475-83

10. Vittinghoff E, McCulloch CE. Relaxing the rule of ten events per variable in logistic and Cox regression. Am J Epidemiol 2007;165(6):710-8

11. Abbott DH, Seed PT, Bennett PR et al. EQUIPP: Evaluation of Fetal Fibronectin with a novel bedside Quantitative Instrument for the Prediction of Preterm birth. Arch Dis Child Fetal Neonatal Ed 2014;99:A150-A51

12. van Baaren GJ, Vis JY, Wilms FF, et al. Predictive value of cervical length measurement and fibronectin testing in threatened preterm labor. Obstet Gynecol 2014;123(6):1185-92

13. DeFranco EA, Lewis DF, Odibo AO. Improving the screening accuracy for preterm labor: is the combination of fetal fibronectin and cervical length in symptomatic patients a useful predictor of preterm birth? A systematic review. Am J Obstet Gynecol 2013;208(3):233 e1-6

14. Ades AE, Sculpher M, Sutton A, et al. Bayesian methods for evidence synthesis in cost-effectiveness analysis. Pharmacoeconomics 2006;24(1):1-19

For peer review only

15. Sculpher MJ, Claxton K, Drummond M, McCabe C. Whither trial-based economic evaluation for health care decision making? Health Econom 2006;15(7):677-87

16. British National Formulary. BNF 73. 2017. .

17. Department of Health. Information Services Division. Scottish Health Service Costs Book 2012. 2012. Information Services Division.

18. National Institute for Health and Care Excellence. Guide to the Methods of Technology Appraisal: 2013. 2013 National Institute for Health and Clinical Excellence.

19. Briggs AH. Handling uncertainty in cost-effectiveness models. Pharmacoeconomics 2000;17(5):479-500

20. Hundley V, Gurney E, Graham W, Rennie AM. Can anxiety in pregnant women be measured using the State-Trait Anxiety Inventory. Midwifery 1998;14(2):118-21

21. http://ipdas.ohri.ca (accessed 23rd November 2017)

22. https://www.tommys.org (accessed 23rd November 2017)

For peer review only

Figure 1

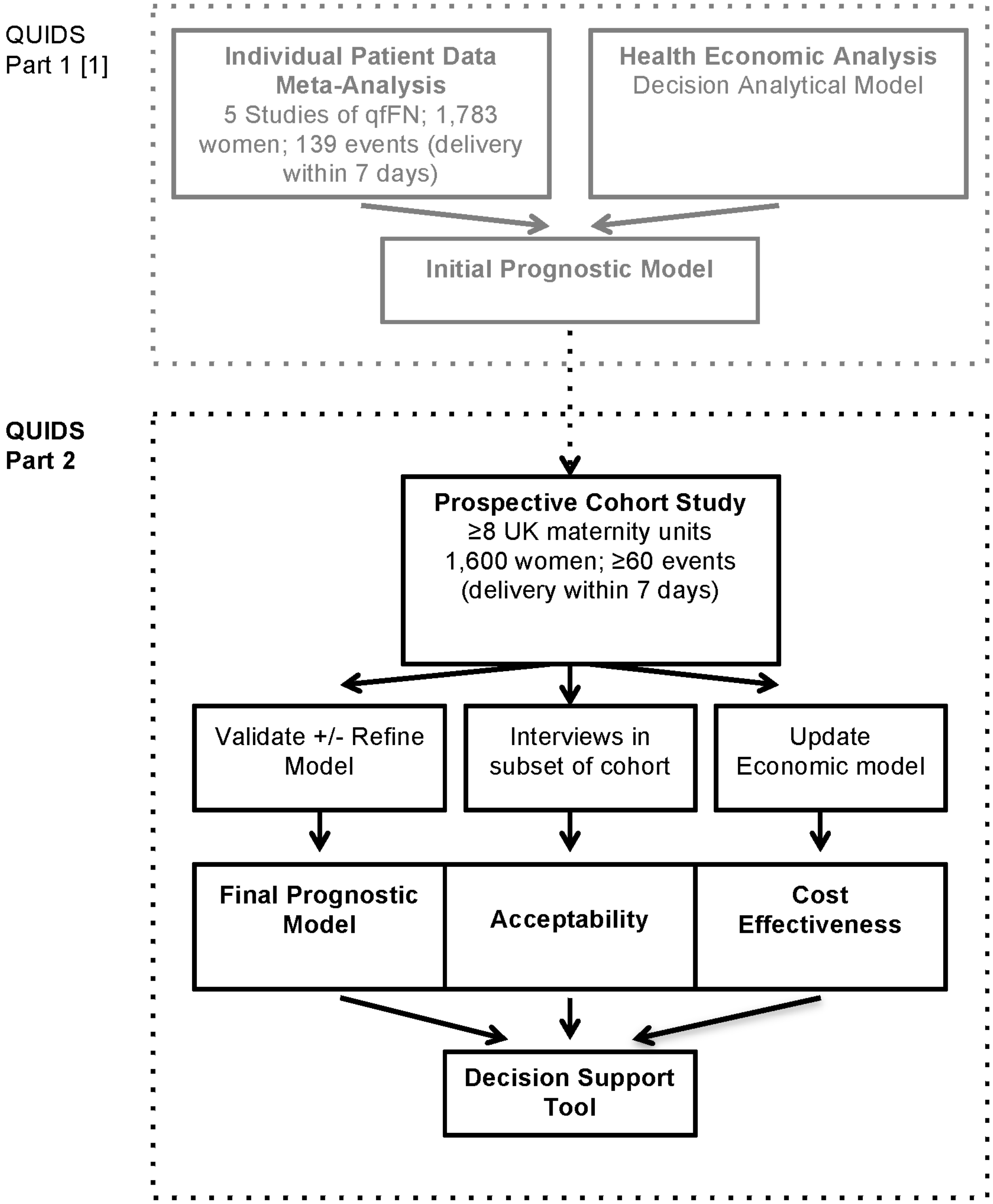
QUIDS
Part 1 [1]
Individual Patient Data Meta-Analysis
5 Studies of qfFN; 1,783 women; 139 events (delivery within 7 days)
Health Economic Analysis
Decision Analytical Model
Initial Prognostic Model
QUIDS
Part 2
Prospective Cohort Study
≥8 UK maternity units
1,600 women; ≥60 events
(delivery within 7 days)
Validate +/- Refine Model
Interviews in subset of cohort
Update Economic model
Final Prognostic Model
Acceptability
Cost Effectiveness
Decision Support Tool

QUIDS Qualitative

Quantitative Fibronectin to help Decision-making in women with Symptoms of Preterm Labour:

determining decisional requirements

Protocol

(October 2015)

White H, O'Brien E, Hodgetts-Moreton V, Stock S, Lavender T

For peer review only

Background

Preterm birth, defined as birth prior to 37 weeks gestation, occurs in 6-7% of pregnancies in Europe[1] and was recorded as 5.78% in England in 2013/14, equating to over 37,000 births.[2] Preterm birth is associated with a high risk of mortality, wide-ranging short- and long-term morbidities,[3,4] and significant economic costs to the NHS compared with birth at term.[5] Reducing the detrimental impact of preterm birth relies on the provision of timely and appropriate perinatal interventions. However, accurate prediction of preterm birth is challenging, even when the clinical symptoms are suggestive of preterm labour. In randomised trials approximately 80% of women diagnosed with preterm labour remained pregnant after 7 days.[6,7]

Interventions in preterm labour and preparations for preterm birth may include administration of corticosteroids to accelerate fetal lung maturation[8,9] and magnesium sulphate for fetal neuroprotection,[10] in utero transfer to a facility with appropriate maternity and neonatal services, and tocolysis to optimise time before birth to enable these.[11] Whilst such interventions can improve outcomes for mothers and babies who do experience preterm birth, they are not necessarily benign, especially for those in whom preterm birth does not occur.

The maximal beneficial impact of corticosteroids occurs with administration between 48 hours and seven days before birth, thus timing is especially important in optimising benefit for the neonate. For women who remain at risk of preterm birth after seven days of the initial dose, repeated doses reduce respiratory distress in the neonate[9] but have been found to be associated with a dose-dependent reduction in birthweight.[12,13] A five-year follow-up study of women who received repeated doses of antenatal corticosteroids due to risk of preterm birth found an increased risk of neurodevelopment impairment in infants born at term.[14] Therefore developing a strategy to establish the optimal time to give steroids is a research priority.

V 1.3
21/10/15

For peer review only - http://bmjopen.bmj.com/site/about/guidelines.xhtml

Magnesium sulphate administration immediately prior to birth has been shown to reduce cerebral palsy,[10] but there is a risk of magnesium toxicity leading to respiratory depression in the mother and, theoretically, the neonate.[15]

Whilst there is no clear beneficial effect of tocolytics on the incidence or outcome of preterm birth,[16] their use is recommended if the days gained prior to preterm birth can be used appropriately, for example transfer to a suitable maternity unit or the administration of drugs to protect the neonate.[11] Tocolysis is linked with various maternal and neonatal complications,[17] hence the need for therapy targeted only for those at risk of preterm birth and close monitoring of the mother and fetus throughout.

Often, inpatient admission is recommended if preterm labour is suspected. Previous literature has highlighted the social isolation and support needs that women with high-risk pregnancies who are hospitalised experience.[18] In some cases, in-utero transfer is indicated to ensure that birth takes place in a specialist unit with appropriate neonatal care facilities. This policy has been shown to reduce mortality[19,20] and morbidity[21] in preterm neonates, especially those born very premature. Qualitative research has indicated that women generally acknowledge the potential benefit of in utero transfer to their baby and, hence, are willing to endure the inconvenience and upheaval that it entails.[22,23] However, the experience is associated with an emotional, social and financial burden on women and their families, especially for the substantial proportion of women who do not deliver prematurely following in utero transfer. When describing their experiences of in utero transfer, women expressed shock at the prospect of the transfer, feeling socially isolated, and having no control over the situation, in addition to the practical difficulties experienced particularly by women who already had children.[22,24,25] In a large survey of women who had experienced in utero transfer, over a quarter lamented the financial cost[24] particularly with respect to their partner's outlay for travel, food, accommodation, and phone bills, exacerbated with requiring time off work.[22] Furthermore, in utero transfer is costly to maternity services. Securing a maternal and neonatal bed

For peer review only - http://bmjopen.bmj.com/site/about/guidelines.xhtml

in another unit is a time-consuming task that often falls to delivery suite midwives to arrange, whilst also continuing to provide care to the woman.[26] In a large observational study of all in utero transfers that took place in Scotland in a six-month period, nearly one third of all transfers were due to threatened preterm labour.[27] Under half of the women transferred from one consultant-led unit to another gave birth within 48 hours.[27] Such unnecessary transfers are costly to women, their families and maternity services. Qualitative research into women's experiences of preterm labour have highlighted the need for caregivers to create an environment where women are enabled to discuss their fears[28] and exert control over how they manage their preterm labour care.[25]

Accurate prediction of preterm birth could reduce the burdens and risks associated with unnecessary interventions, and enable women and their clinicians to make informed decisions regarding their care. Numerous diagnostic tests have been used in preterm labour, including biochemical tests of vaginal secretions and cervical length.[29] One such test is fetal fibronectin, a near-bedside test that provides a positive or negative result and has excellent negative predictive value.[30] Thus fetal fibronectin can identify which women will not benefit and may be put at risk by the interventions described previously, and reduce costs to maternity services.[31] Developments in fetal fibronectin testing have led to a quantitative test that provides a concentration of fetal fibronectin in vaginal secretions, giving women and clinicians more information on which to base their management decisions.[32]

Qualitative evidence has indicated that women feel a sense of increased responsibility to their babies and themselves during a high risk pregnancy, such as threatened preterm labour.[33] Women want to be involved in decision making about their care to different degrees and feel most satisfied when their caregiver supports them to make decisions in the way they felt most comfortable.[33]

V 1.3
21/10/15

For peer review only - http://bmjopen.bmj.com/site/about/guidelines.xhtml

Previous literature on decision making and preterm birth has focussed on diagnostic tests[6,28–32,34] and the care of the preterm infant.[35,36] To date, there has been no investigation of what women, their partners and caregivers would like to know in order to make informed decisions about the care that is provided following the signs and symptoms of preterm labour.

Funding has been received from the National Institute for Health Research Health Technology Assessment Programme for a large, multicentre trial to develop a mobile application decision support tool for the management of women with symptoms and signs of preterm labour, based on a validated model using quantitative fetal fibronectin testing. This study is the precursor to that trial, with the aim of determining the decisional needs of pregnant women with the symptoms and signs of preterm labour, their families and caregivers, using a qualitative framework approach. The outcomes of this qualitative study will inform the development of the mobile application decision support tool, using the findings from an individual patient data meta-analysis. The tool will then be externally validated and refined in the multi-centre trial, QUIDS.

For peer review only - http://bmjopen.bmj.com/site/about/guidelines.xhtml

Methods

A qualitative framework approach will be used, based on data collected from focus groups and semi-structured telephone interviews.

Setting

Focus groups will take place in three maternity units: Liverpool Women's NHS Foundation Trust, Birmingham Women's NHS Foundation Trust and Royal Edinburgh Hospital, NHS Lothian. There will be focus groups for women and a separate focus group for partners. Clinicians who care for women with threatened preterm birth will be interviewed by telephone.

Sample

A purposive sample of women and partners will be recruited to cover a variety of experiences of preterm labour and birth. Women will be stratified by their prior experience and relevant characteristics, including ethnicity, previous obstetric history, living in an urban or rural setting and proximity to a tertiary neonatal referral centre. Two focus groups of 4–8 women will be conducted at each site; one for pregnant women who are at high risk of preterm birth, and one for postnatal women who have recently experienced preterm birth. One partners' focus group will be conducted at one of the sites. If women or partners are unable to attend a focus group but still wish to participate, a semi-structured telephone interview will be offered.

Up to 10 obstetricians, including trainees, midwives, and neonatologists will be purposefully recruited to cover a range of professional backgrounds and experience. Semi-structured telephone interviews will be used to collect the data.

For peer review only - http://bmjopen.bmj.com/site/about/guidelines.xhtml

Eligibility

Principal inclusion criteria for women's antenatal focus groups

Women who are currently pregnant who:

- Have previously experienced preterm birth following preterm labour,
- Have experienced threatened preterm labour in this pregnancy,
- Are at high risk of preterm birth for another clinical reason, such as prior cervical surgery.

Principal inclusion criteria for women's postnatal focus groups

Women who have experienced preterm birth following preterm labour at <34 weeks whose babies **are stable and well** and are receiving care on the special care baby unit or neonatal intensive care unit.

Principal inclusion criteria for partners' focus groups

Partners of women who fit the eligibility criteria for either focus group.

Principal exclusion criteria for the focus groups

Non-English speaking individuals.

Principal inclusion criteria for clinician interviews

Clinicians who care for pregnant women i.e. obstetricians (including trainees), neonatologists and midwives.

Principal exclusion criteria for clinician interviews

V 1.3
21/10/15

For peer review only

Researchers in QUIDS or QUIDS qualitative.

Recruitment

Women and partners

Eligible women will be identified by clinicians in the preterm birth clinic and other antenatal clinics, and antenatal, triage or labour wards (for the antenatal focus groups) and the special care baby unit or postnatal clinics (for the postnatal focus groups) at each site. Eligible partners will be identified by the same method. Clinicians who are aware of and understand the research aims will approach women and partners to request consent for a researcher to contact them. Importantly, only postnatal parents whose babies are being cared for on the SCBU who are considered stable and well by the clinicians will be approached. With consent the researcher will make contact to talk to the women and/or their partners about the research, either face-to-face or over the telephone. Potential participants will be given the participant information sheet (PIS) (appendix _) that is relevant to them and given verbal information about the study. Each participant will be given time to read the information and the opportunity to have any questions answered. Willing participants will be asked to provide their written consent prior to the focus groups.

Clinicians

Eligible clinicians will be approached by the researchers, via email or face-to-face. Clinicians will be given the clinician PIS (appendix _) and the opportunity to read the information and have any questions answered. Willing clinicians will be asked to provide their written consent prior to the interviews.

For peer review only - http://bmjopen.bmj.com/site/about/guidelines.xhtml

All participants (women, partners and clinicians) will be reassured that they are not compelled to participate, that they can withdraw from the study at any time, and that non-participation will not affect their care or employment in any way.

Data collection

The primary aim of this research is to determine the decisional requirements of women, their partners and clinicians for the management of preterm labour. Qualitative semi-structured interviews, in a focus-group setting or individual telephone interviews, provide a means of collecting rich, in-depth data with a specific focus.[37] Hence, structured topic guides will be used to initiate and concentrate the discussion (appendices 7–10).

Focus groups are the preferred format for eliciting the view of women and women's partners. Encouraging discussion among a homogenous group with a shared interest is likely to provide rich insight and understanding into the group's experiences, beliefs and norms as a result of their social interaction.[38] Conversely, interviewing clinicians individually avoids the potential pitfall of professional embarrassment stifling ideas in a group setting. Interviewing individual clinicians with a range of professional experience should ensure that the decisional requirements of clinicians at all levels of experience are understood.

Demographic details and baseline characteristics will be collected prior to the interviews, either as a self-completion questionnaire, or questions asked by the researcher over the telephone. All interviews will be audio recorded, with the participants' consent, and field notes taken. The focus groups will be facilitated by at least two researchers. This is to ensure that all pre-specified areas of interest are covered and that non-verbal communication and group interactions are documented within the field-notes, which will provide context for the data analysis. Recapping will be used to

For peer review only - http://bmjopen.bmj.com/site/about/guidelines.xhtml

clarify aspects and avoid misinterpretation. To enable all participants to talk freely, the researchers will be unknown to the participants and not working clinically in the unit where the interview is conducted. Clinicians will be interviewed by a researcher who is unknown to them.

	Site	Interviewers
Women and partners' focus groups	Liverpool	HW and EO
	Birmingham	HW and VH-M
	Edinburgh	HW and LM
Clinician interviews	Telephone	HW (and EO?)

Analysis plan

A framework approach to data analysis will be used. This approach was developed to manage and interpret large volumes of data collected to inform health policy, meaning they had focussed aims and objectives.[37] Likewise, this research has clear aims, as described previously, in addition to the methodological aim of collecting rich data about the experiences and beliefs of women, their partners and clinicians in relation to managing preterm labour.

Framework analysis follows specific, clearly documented stages of analysis that are transparent so that others can review the interpretation processes and understand how the findings were reached.[39] Transparency is particularly important in this study as the findings will inform the development of an application to aid management decisions in clinical practice. Following verbatim transcription of the interview recordings, the researchers will become familiar with the data by reading the transcripts and field-notes several times. The next stage is to develop a theoretical framework by re-reading the transcripts and making notes as recurring characteristics are

For peer review only - http://bmjopen.bmj.com/site/about/guidelines.xhtml

recognised. The characteristics will then be collated into themes, which are based on the text itself, supported by the field-notes. The resulting thematic framework will be applied back to the transcripts and field-notes to check that it reflects the context of the original data. The transcripts will be coded, so that portions of text are linked to a discrete theme. A sample of transcripts will be independently coded by two people. The data will be charted and indexed to identify the preterm labour or professional experience of the participant, thus enabling the attribution of themes to a particular group. Finally, the content of the charts will be interpreted and mapped against each other to devise themes and sub-themes categories. Once again, this will involve review of the original data. Explanatory accounts will be developed to clarify the data and quotable sections of data will be identified. The final categories will be discussed between the researchers until consensus is met. The researchers will maintain reflexive journals throughout the data collection and analysis stages, recognising and ameliorating, as far as possible, the fact that their presence and assumptions impact on the data and the findings.[40]

This method of data analysis creates a clear audit trail thus ensuring rigour. Each stage of analysis refers back to the original data so that context and meaning is not lost in the final framework of themes and subthemes. The data analysis process will be managed using NVivo software, a qualitative data analysis tool.

Participant withdrawal

Participants may withdraw from the study at any point. However, they will not be able to withdraw use of their data once the prognostic tool is developed.

For peer review only - http://bmjopen.bmj.com/site/about/guidelines.xhtml

Safety

The physical safety of participants will be ensured through adhering to the health and safety policies of the host units where the focus groups take place.

The emotional wellbeing of the participants will be safeguarded by following the Distress Policy (see appendix 11). The Supervisors of Midwives (SOM) team in each unit will be informed of the study and women and their partners will be given the SOM team contact details, should they become distressed or upset as a result of talking about their experiences. Participants will also be given the contact details for accessing local counselling services.

For peer review only

Good clinical practice

Informed consent

All participants will be fully informed about the study and the subsequent QUIDS trial via verbal and written communication. All eligible individuals will be given the participant information sheet (appendix __) and provided with an opportunity to have any questions answered. Written consent will then be gained prior to the commencement of the focus groups/interviews.

Confidentiality

Demographic information will be collected from participants to attribute themes from the data to particular groups within the analysis and dissemination of findings. Demographic information, which will contain potentially identifiable information, will be kept in a secure lockable cabinet. Audio recordings will be stored on an encryptable audio device only until they are transcribed. Once transcribed the audio recordings will be deleted. Transcription services are provided by '1st Class Secretarial', who subscribe to the Data Protection Act and have also signed the Code of Practice on Data Handling. Hard copies of audio transcripts and field-notes will be kept in a separate secure

For peer review only - http://bmjopen.bmj.com/site/about/guidelines.xhtml

lockable cabinet to the demographic information. The transcripts and field-notes will be coded to identify which participant provided that data; the codes will only be known by the researchers. Participant's data will not be used for any purpose other than this study and the subsequent QUIDS trial.

Data Protection

Participants will be informed that publications from this study will contain direct quotes from the focus groups/interviews and categorisation of their experience of preterm labour (e.g. experienced preterm birth), which could enable personal identification.

All researchers involved in this study must comply with the requirements of the Data Protection Act 1998 with regard to the collection, storage, processing and disclosure of personal information and uphold the Act's core principles. All computers used for processing data are password protected and subject to the strict data protection policies of the researcher's institution.

Good clinical practice training

All researchers involved in this study must hold evidence of recent Good Clinical Practice training.

Additional ethical considerations

Expenses and reimbursement

Participants will be reimbursed for all out of pocket expenses, for example travelling to the interview site. Participants will be informed of this and how to apply for expenses reimbursement, including keeping receipts for travel.

For peer review only - http://bmjopen.bmj.com/site/about/guidelines.xhtml

Safety of researchers

An individualised risk assessment will be conducted to identify any risks to researchers or participants involved in this study. The lone working policy of the institution will be adhered to at all times. The only anticipated lone working will be during travel to and from the interview sites.

The lone working policy of the researcher's institutions mandates that researchers wear a GPS tracking and audio transmitting device during all lone-working, off-site research activity with participants. Participants will be informed if this device is being used.

Insurance / Indemnity

The researcher's institution holds public liability insurance and professional indemnity insurance (appendices 12, 13 and 14).

Timeline

The anticipated start date for the focus groups and interviews is 1st January 2016, to be completed within 3 months.

For peer review only
For peer review only - http://bmjopen.bmj.com/site/about/guidelines.xhtml

Appendices

Appendix 1: PIS women

Appendix 2: PIS partners

Appendix 3: PIS clinicians

Appendix 4: Consent form women

Appendix 5: consent form partners

Appendix 6: consent form clinicians

Appendix 7: Interview schedule AN women

Appendix 8: Interview schedule PN women

Appendix 9: Interview schedule partners

Appendix 10: Interview schedule clinicians

For peer review only

V 1.3
21/10/15

Appendix 11: Distress policy

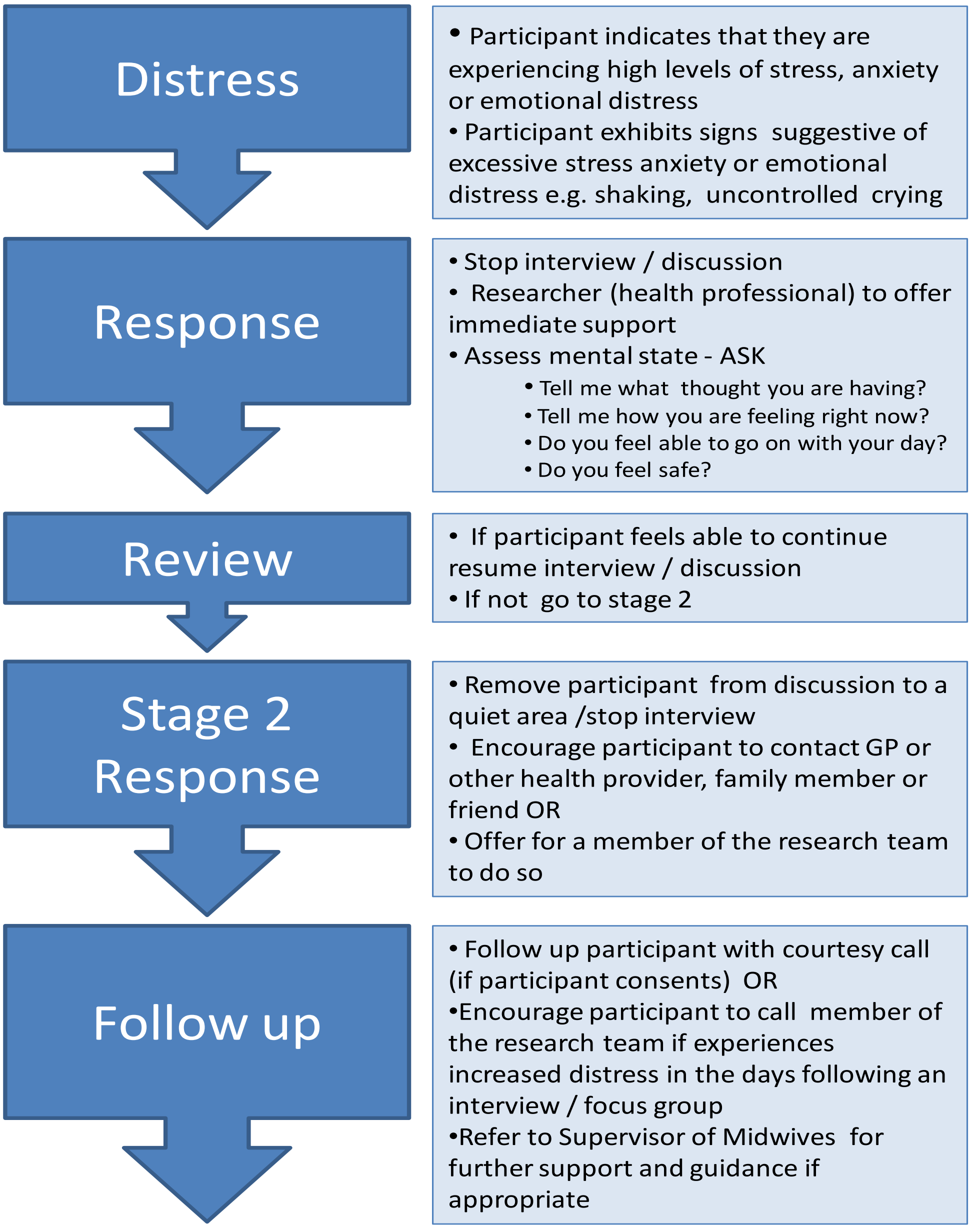

Adapted from Haigh and Witham (2010)[41]

V 1.3
21/10/15

Appendix 12: Public Liability insurance

ZURICH
MUNICIPAL

To Whom It May Concern

Our ref: SP/IND 3 June, 2015

Zurich Municipal Customer: The University of Manchester

This is to confirm that The University of Manchester have in force with this Company Public Liability Insurance until the policy expiry on 31 May 2016:

Policy Number: NHE-07CA03-0013

Limit of Indemnity: £ 50,000,000 any one claim

Excess: Nil any one claim

Yours faithfully

Underwriting Services
Zurich Municipal
Farnborough

Zurich Municipal
Zurich House
2 Gladiator Way
Farnborough
Hampshire
GU14 6GB

Telephone 0870 2418050
Direct Phone 01252 387859
Direct Fax 01252 375893
E-mail alison.cliff@uk.zurich.com

Communications will be monitored regularly to improve our service and for security and regulatory purposes

Zurich Municipal is a trading name of Zurich Insurance Group Ltd

A public limited company incorporated in Ireland. Registration No. 13460 Registered Office: Zurich House, Ballsbridge Park, Dublin 4, Ireland.

UK branch registered in England and Wales Registration No. BR7985. UK Branch Head Office: The Zurich Centre, 3000 Parkway, Whiteley, Fareham, Hampshire PO15 7JZ

Authorised by the Central Bank of Ireland and subject to limited regulation by the Financial Conduct Authority. Details about the extent of our regulation by the Financial Conduct Authority are available from us on request.

V 1.3
21/10/15

Appendix 13: Employers' Liability insurance

ZURICH® MUNICIPAL

Certificate of Employers' Liability Insurance(a)

(Where required by regulation 5 of the Employers' Liability (Compulsory Insurance) Regulations 2008 (the Regulations), a copy of this certificate must be displayed at all places where you employ persons covered by the policy or an electronic copy of the certificate must be retained and be reasonably accessible to each employee to whom it relates).

Policy No.	NHE-07CA03-0013
1. Name of policyholder	The University of Manchester
2. Date of commencement of insurance policy	01 June 2015
3. Date of expiry of insurance policy	31 May 2016

We hereby certify that subject to paragraph 2:

1. The policy to which this certificate relates satisfies the requirements of the relevant law applicable in Great Britain, Northern Ireland, the Isle of Man, the Island of Jersey, the Island of Guernsey and the Island of Alderney **(b)**

2. **(a)** the minimum amount of cover provided by this policy is no less than £5 million **(c)**

Signed on behalf of Zurich Insurance plc (Authorised Insurer).

Signature

S. Lewis

Stephen Lewis

Chief Executive Officer, Zurich Insurance plc (UK Branch)

Zurich Municipal is a trading name of Zurich Insurance plc
A public limited company incorporated in Ireland
Registration No.13460 Registered Office Zurich House, Ballsbridge Park ,Dublin 4 Ireland.
UK branch registered in England and Wales Registration No. BR 7985
UK Branch Head Office
The Zurich Centre, 3000 Parkway, Whiteley, Fareham, Hampshire PO15 7JZ

Authorised by the Central Bank of Ireland and subject to limited regulation by the Financial Conduct Authority. Details about the extent of our regulation by the Financial Conduct Authority are available from us on request

Notes

(a) Where the employer is a company to which regulation 3(2) of the Regulations applies, the certificate shall state in a prominent place, either that the policy covers the holding company and all its subsidiaries, or that the policy covers the holding company and all its subsidiaries except any specifically excluded by name, or that the policy covers the holding company and only the named subsidiaries.

(b) Specify applicable law as provided for in regulation 4(6) of the Regulations.

(c) See regulation 3(1) of the Regulations and delete whichever of paragraphs 2(a) or 2(b) does not apply. Where 2(b) is applicable, specify the amount of cover provided by the relevant policy

V 1.3
21/10/15

Appendix 14: Professional indemnity insurance

MARSH

Cliffe Crowther
Marsh Ltd
Belvedere
12 Booth Street
Manchester
M2 4AW
+44 (0) 161 954 7317
Fax +44 (0) 161 954 7210
Cliffe.crowther@marsh.com
www.marsh.com

To whom it may concern

29th May 2015

Dear Sirs,

CONFIRMATION OF INSURANCE – The University of Manchester and Subsidiary Companies
As requested by the above client, we are writing to confirm that we act as Insurance Brokers to the client and that we have arranged insurance(s) on its behalf as detailed below:

PROFESSIONAL INDEMNITY INSURANCE

INSURERS	Novae Underwriting Ltd.
POLICY NUMBER	003210MMA15C
PERIOD OF INSURANCE	01 June 2015 to 31st May 2016, both dates inclusive.
LIMIT OF INDEMNITY	GBP10,000,000 any one claim and in the aggregate any one insurance period plus costs and expenses.
DEDUCTIBLE	GBP20,000 each & every claim including costs and expenses

INSURANCE BROKERS CII CHARTERED

Registered in England Number: 1507274, Registered Office:
1 Tower Place West, Tower Place, London EC3R 5BU.
Marsh Ltd is authorised and regulated by the Financial Conduct Authority

MARSH & McLENNAN COMPANIES

V 1.3
21/10/15

MARSH

Page 2
29th of May 2015

We have placed the insurance which is the subject of this letter after consultation with the client and based upon the client's instructions only. Terms of coverage, including limits and deductibles, are based upon information furnished to us by the client, which information we have not independently verified.

This letter is issued as a matter of information only and confers no right upon you other than those provided by the policy. This letter does not amend, extend or alter the coverage afforded by the policies described herein. Notwithstanding any requirement, term or condition of any contract or other document with respect to which this letter may be issued or pertain, the insurance afforded by the policy (policies) described herein is subject to all terms, conditions, limitations, exclusions and cancellation provisions and may also be subject to warranties. Limits shown may have been reduced by paid claims.

We express no view and assume no liability with respect to the solvency or future ability to pay of any of the insurance companies which have issued the insurance(s).

We assume no obligation to advise yourselves of any developments regarding the insurance(s) subsequent to the date hereof. This letter is given on the condition that you forever waive any liability against us based upon the placement of the insurance(s) and/or the statements made herein with the exception only of wilful default, recklessness or fraud.

This letter may not be reproduced by you or used for any other purpose without our prior written consent.

This letter shall be governed by and shall be construed in accordance with English law.

Yours faithfully

Cliffe Crowther

MARSH & McLENNAN COMPANIES

V 1.3
21/10/15

References

1. Beck S, Wojdyla D, Say L, *et al.* The worldwide incidence of preterm birth: a systematic review of maternal mortality and morbidity. *Bull World Health Organ* 2010;88:31–38. doi:10.2471/BLT.08.062554.
2. Health and Social Care Information Centre. *Hospital Episode Statistics: NHS Maternity statistics — England, 2013–14.* Leeds, HSIC.
3. Saigal S, Doyle LW. An overview of mortality and sequelae of preterm birth from infancy to adulthood. *Lancet* 2008;371:261–9.
4. D'Onofrio BM, Class QA, Rickert ME, *et al*. Preterm birth and mortality and morbidity: a population-based quasi-experimental study. *JAMA Psychiatry* 2013; 70(11):1231-1240. doi:10.1001/jamapsychiatry.2013.2107 .
5. Mangham LJ, Petrou S, Doyle LW, *et al.* The cost of preterm birth throughout childhood in England and Wales. *Pediatrics* 2009;123(2):e312–27. doi: 10.1542/peds.2008-1827.
6. Alfirevic Z, Allen-Coward H, Molina F, *et al.* Targeted therapy for threatened preterm labor based on sonographic measurement of the cervical length: a randomized controlled trial. *Ultrasound Obstet Gynecol* 2007;29:47–50. DOI: 10.1002/uog.3908.
7. Salim R, Garmi G, Nachum Z, *et al.* Nifedipine compared with atosiban for treating preterm labor: a randomized controlled trial. *Obstet Gynecol* 2012;120:1323–31. DOI:10.1097/AOG.0b013e3182755dff
8. Roberts D, Dalziel SR. Antenatal corticosteroids for accelerating fetal lung maturation for women at risk of preterm birth. *Cochrane Database of Systematic Reviews* 2006, Issue 3. Art. No.: CD004454. DOI: 10.1002/14651858.CD004454.pub2.
9. Crowther CA, McKinlay CJD, Middleton P, Harding JE. Repeat doses of prenatal corticosteroids for women at risk of preterm birth for improving neonatal health outcomes.

For peer review only - http://bmjopen.bmj.com/site/about/guidelines.xhtml

Cochrane Database of Systematic Reviews 2015, Issue 7. Art. No.: CD003935. DOI: 10.1002/14651858.CD003935.pub4.

10. Doyle LW, Crowther CA, Middleton P, Marret S, Rouse D. Magnesium sulphate for women at risk of preterm birth for neuroprotection of the fetus. *Cochrane Database of Systematic Reviews* 2009, Issue 1. Art. No.: CD004661. DOI:10.1002/14651858.CD004661.pub3.
11. Royal College of Obstetricians and Gynaecologists. *Tocolysis for women in preterm labour: Green-top Guideline No. 1b.* 2011. London: RCOG.
12. Murphy KE, Hannah ME, Willan AR, Hewson SA, Ohlsson A, Kelly EN, Matthews SG, Saigal S, Asztalos E, Ross S, Delisle M-F, Amankwah K, Guselle P, Gafni A, Lee SK, Armson BA, for the MACS Collaborative Group. Multiple courses of antenatal corticosteroids for preterm birth (MACS): a randomised controlled trial. *Lancet* 2008; 372: 2143–51.
13. Norberg H, Stålnacke J, Diaz Heijtz R, Smedler A-C, Nyman M, Forssberg H, Norman M. Antenatal corticosteroids for preterm birth: dose-dependent reduction in birthweight, length and head circumference. *Acta Paediatrica* 2011;100:364–9.
14. Asztalos E, Willan A, Murphy K, Matthews S, Ohlsson A, Saigal S, Armson A, Kelly E, Delisle M-F, Gafni A, Lee S, Sananes R, Rovet J, Guselle P, Amankwah K, for the MACS-5 Collaborative Group. Association between gestational age at birth, antenatal corticosteroids, and outcomes at 5 years: multiple courses of antenatal corticosteroids for preterm birth study at 5 years of age (MACS-5). *BMC Pregnancy and Childbirth* 2014; 14:272.
15. Jacquemyn Y, Zecic A, Van Laere D, Roelens K. The use of intravenous magnesium in non-preeclamptic pregnant women: fetal/neonatal neuroprotection. *Arch Gynecol Obstet* 2015;291:969–975.
16. Gyetvai K, Hannah ME, Ellen D, Hodnett ED, Ohlsson A. Tocolytics for preterm labour: a systematic review. *Obstetrics and Gynecology* 1999;94(5):869–77.
17. Hill WC. Risks and complications of tocolysis. *Clinical Obstetrics and Gynecology* 1995;38(4):725–45.

For peer review only

For peer review only - http://bmjopen.bmj.com/site/about/guidelines.xhtml

18. Kent RA, Yazbek M, Heyns T, et al. The support needs of high-risk antenatal patients in prolonged hospitalisation. *Midwifery* 2015;31:164–9.

19. Lasswell SM, Barfield WD, Rochat RW, Blackmon L. Perinatal Regionalization for Very Low-Birth-Weight and Very Preterm Infants: A Meta-analysis. *JAMA.* 2010;304(9):992-1000. doi:10.1001/jama.2010.1226.

20. Marlow N, Bennett C, Drasper ES, Hennessy EM, Morgan AS, Costeloe KL. Perinatal outcomes for extremely preterm babies in relation to place of birth in England: The EPICure 2 study. *Arch Dis Child Fetal Neonatal Ed* 2014;99:F181–88.

21. Towers TV, Bonebrake R, Padilla G, Rumney P. The effect of transport on the rate of severe intraventricular hemorrhage in very low birth weight infants. *Obstetrics and Gynecology* 2000;95(2):291–295.

22. Porcellato L, Masson G, O'Mahony F, Jenkinson S, Vanner T, Cheshire K, Perkins E. 'It's something you have to put up with'— service users' experiences of in utero transfer: a qualitative study. *BJOG* 2015; DOI: 10.1111/1471-0528.13235.

23. Bond PA, Crisp AS, Morgan MEI, Lobb MO, Cooke RWI. Maternal attitudes to transfer before delivery. *Journal of Reproductive and Infant Psychology* 1984;2(1):33–41. DOI: 10.1080/02646838408403447.

24. Wilson AM, MacLean D, Skeoch CH, Jackson L. An evaluation of the financial and emotional impact of *in utero* transfers upon families: a Scotland-wide audit. *Infant* 2010;6(2):38–40.

25. Coster-Schulz MA, Mackey MC. The preterm labour experience: a balancing act. *Clinical Nursing Research* 1998;335–59.

26. Gale C, Hay A, Phillip C, et al. In-utero transfer is too difficult: results from a prospective study. *Early Human Development* 2012;88:147–50.

27. Macintyre-Beon C, Skeoch C, Jackson J, Booth P, Cameron A. *Perinatal Collaborative Transport Study: Final report.* 2008. Glasgow: Scottish Neonatal Transport Service.

V 1.3
21/10/15

For peer review only - http://bmjopen.bmj.com/site/about/guidelines.xhtml

28. O'Brien ET, Quenby S, Lavender T. Women's view of high risk pregnancy under threat of preterm birth. *Sexual and Reproductive Health* 2010;1:79–84.

29. Hezelgrave NL, Shennan AH, David AL. Tests to predict imminent delivery in threatened preterm labour. *BMJ* 2015;350:h2183 doi: 10.1136/bmj.h2183.

30. Chandiramani M, Di Renzo GC, Gottschalk E, Helmer H, Henrich W, Hoesli I, Mol B, Norman JE, Robson S, Thornton S, Shennan A. Fetal fibronectin as a predictor of spontaneous preterm birth: a European perspective. *The Journal of Maternal-Fetal & Neonatal Medicine* 2011;24(2):330–336.

31. Deshpande SN, van Asselt ADI, Tomini F, Armstrong N, Allen A, Noake C, et al. Rapid fetal fibronectin testing to predict preterm birth in women with symptoms of premature labour: a systematic review and cost analysis. *Health Technol Assess* 2013;17(40).

32. Radford S, Abbott D, Seed P, Kemp J, Shennan. Quantitative fetal fibronectin for the prediction of preterm birth in symptomatic women. *Arch Dis Child Fetal Neonatal Ed* 2012;97(Supp 1):A1–A125.

33. Harrison MJ, Kushner KE, Benzies K, et al. Women's satisfaction with their involvement in health care decisions during a high-risk pregnancy. *Birth* 2003;30(2):109–15.

34. Hill JL, Campbell MK, Zou GY, et al. Prediction of preterm birth in symptomatic women using decision tree modelling for biomarkers. *Am J Obstet Gynecol* 2008;198:468.e1-468.e9.

35. Gallagher K, Martin J, Keller M, et al. European variation in decision-making and parental involvement during preterm birth. *Arch Dis Child Fetal Neonatal Ed* 2014;99:F245–9.

36. Peerzada JM, Schollin J, Håkansson S. Delivery room decision-making for extremely preterm infants in Sweden. *Pediatrics* 2006;117(6):1988–95.

37. Smith J, Firth J. Qualitative data analysis: the framework approach. *Nurse Researcher* 2011;18(2):52–62.

38. Rabiee F. Focus group interview and data analysis. *Proceedings of the Nutrition Society* 2004;63: 655–660.

For peer review only - http://bmjopen.bmj.com/site/about/guidelines.xhtml

For peer review only

39. Furber C. Framework analysis: a method for analysing qualitative data. *African Journal of Midwifery and Women's Health* 2010;4(2):97–100.

40. Sword W. Accounting for presence of self: reflections on doing qualitative research. *Qualitative Health Research* 1999;9(2):270–8.

41. Haigh C, Witham G. *Distress protocol for qualitative data collection.* 2015. [Online] available at: http://www2.mmu.ac.uk/media/mmuacuk/content/documents/rke/Advisory%20Distress%20Protocol.pdf accessed October 2015.

For peer review only

For peer review only - http://bmjopen.bmj.com/site/about/guidelines.xhtml

Study Protocol: Quantitative Fibronectin to help Decision-making in women with Symptoms of Preterm Labour (QUIDS) Part One- Individual Patient Data Meta-analysis and Health Economic Analysis

Sarah J Stock*[1,2] Sarah.Stock@ed.ac.uk

Lisa Wotherspoon[1] Lisa.Wotherspoon@ed.ac.uk

Kathleen Boyd[3] Kathleen.Boyd@glasgow.ac.uk

Rachel K Morris[4] r.k.morris@bham.ac.uk

Jon Dorling[5] Jon.Dorling@nottingham.ac.uk

Lesley Jackson[6] Lesley.Jackson@ggc.scot.nhs.uk

Manju Chandiramani[7] m.chandiramani@imperial.ac.uk

Anna David[8] a.david@ucl.ac.uk

Asma Khalil[9] akhalil@sgul.ac.uk

Andrew Shennan[10] andrew.shennan@kcl.ac.uk

Victoria Hodgetts-Morton[4] v.a.h.morton@bham.ac.uk

Tina Lavender[11] Tina.Lavender@manchester.ac.uk

Khalid Khan[12] k.s.khan@qmul.ac.uk

Susan Harper-Clarke[13] su_clarke@hotmail.com

Ben Mol[14] ben.mol@adelaide.edu.au

Richard D. Riley[15] r.riley@keele.ac.uk

John Norrie[16] J.Norrie@ed.ac.uk

Jane Norman[1] Jane.Norman@ed.ac.uk

1. Tommy's Centre for Maternal and Fetal Health, University of Edinburgh MRC Centre for Reproductive Health, Queen's Medical Research Institute, Edinburgh, UK. 2. School of Women's and Infants' Health, University of Western Australia, Crawley WA 6009, AUS. 3. Institute of Health & Wellbeing Health Economics & Health Technology Assessment, University of Glasgow, 1 Lilybank Gardens, Glasgow, UK. 4. School of Clinical and Experimental Medicine, University of Birmingham, Birmingham, UK. 5. Queen's Medical Centre, Neonatal Unit, Derby Road,

For peer review only

Nottingham, UK. 6. Royal Hospital for Children Glasgow, Neonatal Unit Glasgow, UK. 7. Imperial College Healthcare NHS Trust, Queen Charlotte and Chelsea Hospital, Du Cane Road, Shepherds Bush, London, UK. 8. University College London Medical School, Institute for Women's Health, 86-96 Chenies Mews, University College London Medical School London, UK. 9. St. George's Medical School, University of London, London, UK. 10. Kings College London, Maternal and Fetal Research Unit Maternal and Fetal Research Unit 10th Floor, North Wing London, UK. 11. University of Manchester School of Nursing, Midwifery & Social Work, Jean McFarlane Building, University Place, Oxford Road Manchester, UK. 12. Queen Mary, University of London, Centre for Primary Care and Public Health London, UK. 13. Patient and Public Involvement Representative. No affiliation. 14. University of Adelaide, The Robinson Institute, School of Paediatrics and Reproductive Health Adelaide, SA, AUS. 15. Research Institute for Primary Care and Health Sciences, Keele University, Keele, UK. 16. Edinburgh Clinical Trials Unit, University of Edinburgh No. 9, Bioquarter Edinburgh, UK.

*Corresponding Author: Dr Sarah J Stock Senior Clinical Lecturer Maternal and Fetal Medicine, MRC Centre for Reproductive Health, Queen's Medical Research Institute, 47 Little France Crescent, Edinburgh, EH16 4TJ. Tel: 0131 242 6449 Email: sarah.stock@ed.ac.uk

Word Count: 3,582

Key Words: Pregnancy; Preterm Birth; Fetal Fibronectin; Health Economics; Individual Patient Data Meta-analysis.

For peer review only

ABSTRACT

Introduction: The aim of the QUIDS study is to develop a decision support tool for the management of women with symptoms and signs of preterm labour, based on a validated prognostic model using quantitative fetal Fibronectin (fFN) concentration, in combination with clinical risk factors.

Methods and analysis: The study will evaluate the Rapid fFN 10Q System (Hologic, Malborough, MA) which quantifies fFN in a vaginal swab. In part one of the study we will develop and internally validate a prognostic model using an individual participant data (IPD) meta-analysis of existing studies containing women with symptoms of preterm labour alongside fFN measurements and pregnancy outcome. An economic analysis will be undertaken to assess potential cost-effectiveness of the qfFN prognostic model. The primary endpoint will be the ability of the prognostic model to rule out spontaneous preterm birth within seven days. Six eligible studies were identified by systematic review of the literature and five agreed to provide their IPD (n= 5 studies, 1,783 women and 139 events of preterm delivery within 7 days of testing).

Ethics and dissemination: The study is funded by the National Institute of Healthcare Research Health Technology Assessment (HTA 14/32/01). It has been approved by the West of Scotland Research Ethics Committee (16/WS/0068).

Registration details: This IPD Meta-analysis is registered with PROSPERO (PROSPERO 2015:CRD42015027590).

Version: Protocol Version 2, Date 1st November 2016

STRENGTHS AND LIMITATIONS OF THIS STUDY

Strengths

- Development of prognostic model and for validation in a separate prospective cohort study

For peer review only

- Health Economic Analysis to determine cost effectiveness from NHS perspective

Limitations

- Not a randomized control trial to test effectiveness of the model on improved patient outcomes

HOW PATIENTS ARE INVOLVED IN THIS STUDY

Patient representatives were consulted during the protocol development and have been invited to join the Project Management Group and the Trial Steering Committee. Prior to commencing QUIDS, we performed a qualitative study to determine the decisional needs of pregnant women with signs and symptoms of preterm labour, their partners and their caregivers. This is described in the separate protocol “QUIDS Qualitative” (Supplementary Material). The end product of QUIDS will be a decision support aid to help clinicians, women and their partners decide on management of threatened preterm labour, based on the results of the quantitative fFN. In QUIDS Qualitative women and clinicians indicated that they would prefer this to be on web based or mobile app based format, presenting the risk of preterm birth within seven days of testing.

For peer review only

INTRODUCTION

The overall aim of the QUIDS study is to develop a decision support tool for the management of women with symptoms and signs of preterm labour, based on a validated prognostic model using quantitative fFN testing. The study has been conceptually divided into two parts. In this, the protocol for QUIDS Part One, we detail the protocol for development and internal validation of the prognostic model. In the protocol for QUIDS Part Two we detail the protocol for the prospective cohort for external validation of the prognostic model and acceptability testing.[1]

Preterm delivery (before 37 weeks) occurs in 7.1% of pregnancies in the UK (>50,000 deliveries per annum), with the majority the result of preterm labour.[2,3] It remains the leading cause of neonatal morbidity and mortality, but timely interventions, such as antenatal steroids to promote lung maturity, magnesium sulphate for neuroprotection, and delivery in a unit with appropriate neonatal care facilities can improve neonatal outcome. Establishing a diagnosis of preterm labour is, however, difficult. Clinical signs are non-specific and false positive diagnoses are common, with up to 80% of women with signs and symptoms of preterm labour remaining pregnant after seven days. [4, 5] Such diagnostic uncertainty means a large proportion of women with symptoms of preterm labour are treated unnecessarily to ensure benefits to the small proportion of babies that do actually do deliver preterm.

It is understandable that both clinicians and pregnant women may prefer a ‘treat-all’ approach in women with symptoms of preterm labour, particularly in a setting remote from an appropriate neonatal unit; and in order to ensure steroid prophylaxis in case preterm delivery occurs. However, unnecessary interventions result in both a substantial economic burden to health services and in potential adverse maternal and neonatal events. Hospital admission and inter-hospital transfer have

For peer review only

considerable cost implications and can be associated with enormous problems for women and their families due to physical separation and emotional stress.[6,7] Neonatal cots become ‘blocked’ in order to accept a preterm baby just in case delivery occurs; negatively impacting the efficiency of already stretched neonatal units and networks. This frequently has knock-on effects to other women and babies, who may need transfer to another unit due to lack of cot availability despite an empty, but ‘blocked’, cot. It also may increase the number of *ex utero* transfers, which are associated with poorer outcomes than *in utero* transfers.[8] If preterm labour has been wrongly diagnosed, and delivery does not occur, steroids may also have adverse long-term consequences for the baby, especially if multiple courses are given.[9] Tocolytic therapy, even when appropriate can have serious side effects for both mother and baby.[10] Lastly, uncertainty of outcome may contribute to the high anxiety scores seen in women with threatened preterm labour and their partners.[11]

Diagnostic tests for preterm labour are available and used in many units in the UK. Fetal Fibronectin (fFN; Hologic, Marlborough, MA, USA) is a biochemical marker of preterm labour that can be measured in samples of cervicovaginal secretions collected at a speculum examination. It has potential to help improve diagnosis of impending preterm delivery.[12] Other biochemical tests which are available include Actim Partus (Medixbiochemica, Espoo, Finland)) which measures phosphorylated insulin-like growth factor binding protein-1 (phIGFBP-1), and Partosure (Parsagen Diagnostics, Boston, MA, USA) which measures placental alpha microglobulin-1 (PAMG-1). An alternative approach (which can be combined with fFN) is to measure the cervical length using transvaginal ultrasound, as the longer the cervix is, the less likely a preterm delivery.[12]

For peer review only

As part of an Health Technology Assessment (HTA) report Honest et al found that a qualitative fFN test (giving a positive or negative result based on a single threshold of 50ng/ml) was potentially useful in the prediction of preterm delivery <34 weeks gestation, with its main benefit relating to its high negative predictive value i.e. its ability to rule out impending delivery.[12] A more recent HTA-funded review found that qualitative fFN testing has moderate accuracy for predicting preterm birth with overall sensitivity and specificity estimates of 76.7% and 82.7% for delivery within 7-10 days.[13] These estimates suggest that qualitative testing on its own would not have the sensitivity to rule out preterm delivery adequately, although in systematic review of clinical trials, no increase in neonatal morbidity or mortality was seen in association with false negative fFN results.[13] The authors concluded that this observation is likely to relate to the multifactorial nature of assessment of the risk of preterm delivery, where, in practice, fFN is just one component of the clinical assessment on which management decisions are based.[13]

Both HTA reviews described above examined the performance of a qualitative fFN test, which provided a positive or negative result on the basis of a single threshold of 50ng/ml. Recently, this test has been replaced in the UK with the Rapid fFN 10Q System, which provides a concentration of fFN within 10 minutes, and thus may be a more useful predictor of preterm delivery (quantitative fFN). We surveyed current practice in UK maternity units (response rate 66% [137/207]; Mar-July 2014).[14] 135/137 units (98.5%) use some sort of diagnostic test of preterm labour. The most common test is fFN (84/137 units; 61.3%). fFN is now only available with a quantitative analyser in the UK, but there is no consensus as to which women to use the test in, or how to interpret the results. Developing and evaluating a decision support for qfFN is thus likely to improve decision making, even if qfFN is already available in clinical practice. Evidence about the potential value of the new

For peer review only - http://bmjopen.bmj.com/site/about/guidelines.xhtml

quantitative fFN is required, along with guidance about how to interpret results. The QUIDS study will address this evidence gap.

METHODS AND ANALYSIS

Aims and Methodologies

The aim of the QUIDS study is to develop a decision support tool for the management of women with symptoms and signs of preterm labour, based on a validated prognostic model using quantitative fFN testing.

The study protocol has been divided into two parts (see flow chart Figure 1). The protocols for Parts One and Two are reported in separate manuscripts.

Part 1: Development and Internal Validation of Prognostic Model

i) Individual Participant Data (IPD) meta-analysis to develop a prognostic model using quantitative fFN and other risk (prognostic) factors and to evaluate the added value of quantitative fFN toward this prognostic model performance. A prognostic model will be developed and internally validated[15,16] based on a meta-analysis of IPD from existing prospective cohort studies where quantitative fFN results and pregnancy outcome details are available. The primary outcome will be prediction will be delivery within 7 days, although other endpoints will be included if recommended by focus groups.

(ii) Economic Analysis: To provide an economic rationale for the prognostic model and analyze its cost-effectiveness from the perspective of the NHS to provide an economic rationale for the prognostic model and the risk factors included in it.

Part 2: Validation And Refinement Of Prognostic Model Involves a prospective cohort study and acceptability testing, with external validation, (and, if necessary, refinement) of the prognostic model, and update of health economic model.[1]

For peer review only - http://bmjopen.bmj.com/site/about/guidelines.xhtml

Endpoints

The primary endpoint is spontaneous preterm delivery within seven days of qfFN test, in women tested at less than 36 weeks gestation. This is both an important endpoint for women and caregivers (determined in QUIDS Qualitative study – a preceding qualitative study to identify the decisional needs of women, their partners and clinicians; Supplementary Material) as well as a clinically important endpoint. Antenatal steroids (which significantly reduce morbidity and mortality in preterm babies[17]) are most effective if delivery occurs within seven days of administration. As repeated doses of antenatal steroids may be harmful, it is crucial to ensure steroids are timed correctly.

A secondary endpoint suggested by the preceding QUIDS Qualitative Study consultation (Supplementary Material), was delivery within 48 hours of qfFN test. This analysis will be performed if feasible to do so within the constraints of the data available for model development.

Health technologies being assessed

The study will evaluate the Rapid fFN 10Q System (Hologic), which provides a concentration of fFN (ng/ml or INVALID) in a vaginal swab sample. Further details about the system and recommended sampling technique are provided in the QUIDS Protocol Part Two. [1]

Target population

The target population is pregnant women attending hospital with signs and symptoms of preterm labour.

Development Of Prognostic Model

For peer review only

Individual Patient Data Meta-Analysis

The proposed IPD-Meta-analysis was registered on PROSPERO (2015:CRD42015027590). Our IPD meta-analytical approach will follow existing guidelines, and our output will comply with the TRIPOD statement (Transparent reporting of a multivariable prediction model for individual prognosis or diagnosis statement).[18]

Inclusion Criteria

We prespecified inclusion of prospective cohort studies or RCTs of women with signs and symptom of preterm labour (as defined by investigators) that include quantitative fFN results determined by 10Q rapid fFN analyzer system and pregnancy outcome data; and the Principal Investigator of which has agreed to collaborate and provide data.

Exclusion criteria

We will exclude studies where fFN concentration was measured by ELISA and studies where IPD is not available for meta-analysis

Search Strategy

When applying for funding for this study (April 2014) we performed a literature search for completed and ongoing cohort studies of quantitative fFN using search terms for quantitative fetal/foetal fibronectin and preterm birth, including databases (MEDLINE, EMBASE, Cochrane Database of Systematic Reviews (CDSR), Database of Abstracts of Reviews of Effects (DARE) and Health Technology Assessment Database (HTA)) and clinical trial registries (Cochrane Central Register of Controlled Trials (CENTRAL), ClinicalTrials.Gov) general search engines (such as Google: https://www.google.co.uk) and systematic reviews. We also consulted preterm birth

For peer review only - http://bmjopen.bmj.com/site/about/guidelines.xhtml

researchers and networks (RCOG CSG; BMFMS, PREBIC) and the manufacturers of quantitative fFN, (Hologic) to help ensure capture of all relevant studies.

Study manuscripts and/or protocols were screened by two researchers. We identified a total of 10 studies of quantitative fFN that were potentially eligible. Four early datasets (in three manuscripts) used ELISA to determine the concentration of fFN and were excluded as the different method of analysis and earlier period of study would increase heterogeneity.[5,19,20] Therefore, six studies fulfilled the eligibility criteria (see Table 1).

Establishment of the quantitative fFN IPD Collaboration

We contacted the principal investigators (PIs) of the six eligible studies of qfFN invited them to participate (see Table 1). Five of these agreed to provide their IPD as evidenced by their involvement as co-applicants on the funding application and/or co-authorship of this protocol (Mol, van Baaren, Khalil, Shennan, David). The PI of the 6th study (Elovitz) indicated IPD may be available after publication of her study.

The five included studies (Table 1) are European studies of women with symptoms of preterm labour, comprising 1,783 women and 139 events of preterm delivery within 7 days of testing. They are from consultant led maternity units in the UK (three studies) and Europe (two studies). All women in the included trials provided informed consent for participation in clinical trials, and for their IPD to be used in subsequent analyses.

For peer review only - http://bmjopen.bmj.com/site/about/guidelines.xhtml

	PI	Setting	N	Events	Dates	Inclusion	Primary Outcome
**Studies with data available**							
EQUIPP [21,22]	Prof A Shennan	5 UK centres	452	14	2010-2012	22-35 weeks with symptoms of preterm labour	Delivery <34 weeks gestation
EUFIS* [23]	Prof BW Mol	10 European Hospitals	452	48	2012-2014	24-34 weeks with preterm contractions and intact membranes	Delivery within 7 days of test
APOSTEL I* [24]	van Baaren	10 Dutch Hospitals	528	70	2009 -2012	24-34 weeks with preterm contractions and intact membranes	Days to delivery truncated at 7 days
QFCAPS (unpublished)	Dr A Khalil	London teaching hospital	86	2	2012-2014	24-34 weeks with symptoms of preterm labour Singletons only	Delivery within 7 days of test
UCLH/Whit (unpublished)	Dr A David	2 UK centres	262	5	2009-2010	22-35 weeks with symptoms of preterm labour	Delivery within 7 days of test
	TOTALS	***4 studies***	***1,783***	***139***			
**Studies where data may be available in future**							
STOP study (http://clinicaltrials.gov/show/NCT01868308)	Prof M Elovitz	USA teaching hospital	700	NK	2011-2015	22 -34 weeks Symptomatic women with singleton pregnancy	Delivery before 37 weeks

Table 1: Details of studies contributing data to IPD meta-analysis.

*Study unpublished at time of search in April 2014; manuscript now published

For peer review only - http://bmjopen.bmj.com/site/about/guidelines.xhtml

Study Quality Assessment and Data Collection

IPD will be stored in a bespoke database on a secure server at the University of Edinburgh. PIs will be asked to provide de-identified data, and consider all recorded variables (even if not reported publications). We will assess study quality according to QUADAS-2[25] QUIPS[26] and CHARMS[27] guidelines.

Sample Size Considerations

The size of the IPD meta-analysis is limited by the number of studies with data available (Table 1). In model development the number of covariates that can be considered is limited by the number of events, with guidance suggesting at least ten events required for each covariate.[28,29] In our IPD meta-analysis data we have 139 events (preterm labour within 7 days of testing) and therefore deemed that it was sensible to evaluate quantitative fFN and up to 13 other factors (covariates) for potential inclusion in our model.

Data Items

The following factors which are thought to influence risk of spontaneous preterm birth, will be requested and considered for inclusion as covariates in the prognostic model: quantitative fFN concentration, previous spontaneous preterm labour, gestation at fFN test, age, ethnicity, BMI, smoking, deprivation index, number of uterine contractions in set time period, cervical dilatation, vaginal bleeding, previous cervical treatment for cervical intraepithelial neoplasia, cervical length (measured by transvaginal cervical length), singleton/multiple pregnancy, tocolysis and fetal sex. Up to 13 of these will be prespecified for inclusion, based on available data (we will only use variables which are available in each study), and ranking for likely clinical relevance as agreed by consensus of the project management team.

Data Cleaning

Prior to analysis data will be checked for outliers and missing data will be identified. Descriptive statistics will be performed to summarise data. Problems identified will be discussed with the PI of the original study, and amended as indicated by consensus discussion.

Data Analysis and Prognostic Model Development

Multivariable logistic regression modelling will be the primary method of analysis. The primary endpoint for the prognostic model will be delivery within seven days. Another endpoint found to be important in focus group consultations performed in QUIDS Qualitative (Supplementary Material) included delivery within 48 hours, and we will use this as a secondary endpoint if feasible (i.e. if sufficient number of cases with delivery within 48 hours). We will develop an initial model with quantitative fFN concentration, and then consider a model with other predefined clinical predictor variables (see *Data Items*, above).

Tocolysis (which may delay onset of labour, although likely not beyond 48 hours) will be included as a categorical variable (administered/not administered). We will explore treatment effect by sensitivity analysis with and without the assumption that tocolysis could delay delivery within 48 hours by a maximum odds ratio of 5.39, 95% credible interval 2.14 to 12.34, based on data in Haas et al.[30].

As the outcome is binary, a logistic regression modelling framework will be used to develop the model. A multi-level structure will be used to account for clustering of patients within studies, and heterogeneity of the effects of included factors (hereafter called ‘predictors’) will be accounted for using random-effects, with between-study heterogeneity quantified using the estimated variance (‘tau-squared’) and the I^2 statistic. A separate intercept term per study will be included in the model, to account for the clustering and also guage how predictions may require tailoring to different populations. Predictors with large heterogeneity in the prognostic effect across

studies may be removed to ensure summary Beta terms in the model are meaningful (accurate) for individual populations.[16]

In the primary analysis, we will use data from the first recorded attendance with signs and symptoms of preterm labour to determine the relationship between that individual episode and outcome. Data from subsequent attendances will be analysed subsequently, and may be included in an appropriate model. As a parsiminous model is sought, to reduce the factors included in the model that may otherwise delay its use, we will use backward stepwise selection based on an information criterion (e.g. Akaike's information criterion $p<0.15$) to identify a parsimonious set of factors to be included in the model; hereafter these are referred to as included 'predictors'. Further, an approach of adding specialist tests, such as cervical length, only after considering simpler clinical assessment will be used, to maximise the utility of the model by ensuring that extra tests with their additional costs are only be included if they add to the predictive power.

Linearity between continuous variables and outcome will be assessed using cubic spline plots and data will transformed where appropriate before inclusion in multivariable analysis (e.g. using fractional polynomial methods). Missing data will be assessed to determine whether missing at random is appropriate, and if so, multiple imputation of observed participant characteristics will be used, with missing data imputed within each original study separately, before the meta-analysis. The results of these analyses will be compared with a complete case analysis.

Assessing Apparent Model Performance

The apparent performance of the model will be assessed by its overall fit, and the observed discrimination and calibration in the IPD used to develop the modle. Overall fit of the models will be expressed with Nagelkerke R^2. The ability of the models to discriminate between women with and without spontaneous preterm birth will be

For peer review only

determined by the area under the receiver operating characteristics curve (AUC), also known as the *C* statistic. Agreement between predicted and observed proportions of women with spontaneous preterm birth will be visualized using a calibration plot, and measured using calibration slope and calibration-in-the-large.

Internal validation: assessing Optimism In Model Performance

Apparent performance is likely to be optimistic, as it is examined in the same data used for model development. Therefore internal validation will also be undertaken using a non-parametric bootstrap re-sampling technique in which each modelling step is repeated in each bootstrap sample, to obtain a new model in each bootstrap sample, and then its apparent performance (AUC and calibration slope) in the bootstrap sample is compared to its performance in the original dataset. The 'optimism' is the mean difference (across all bootstrap samples) between the apparent value in the bootstrap sample and the observed value in the original dataset. This optimism estimate is then subtracted from the original model's apparent performance, to give an optimism-adjusted estimate of each measure of performance for the original model (e.g. R^2, *C* statistic, Calibration slope).

Production Of Final Model From IPD Meta-Analysis Via Uniform Shrinkage

The optimism-adjusted calibration slope will be used as a uniform shrinkage factor, to adjust the parameter estimates (log odds ratios) of the original model. The beta coefficients in the original model will be multiplied by the shrinkage factor, and the study intercept terms re-estimated to ensure perfect overall calibration is maintained (across all studies and, ideally, in each study separately). This will thereby produce a final model containing the updated intercepts and the shrunken beta coefficients.[31] With multiple intercepts, a strategy (or strategies) will be developed amongst the study investigators for which intercept should be chosen for use when externally validating the model in a new population (e.g. choose intercept from study that most

For peer review only

closely resembles the population of application); each strategy will be evaluated and compared in the cohort study external validation phase.

Added Value Of Quantitative fFN

The added value of quantitative fFN will be examined throughout the whole model process, in particular its improvement on discrimination, calibration and other meaningful factors (such as clinical decisions) using appropriate techniques (such as net reclassification improvement and decision analysis methods).

Subgroup analyses

Subgroup analysis will be performed for multiple pregnancy, women with a previous preterm labour, gestation and those with criteria that are suggested to indicate preterm labour (number of uterine contractions in a set time period and/or cervical change). This will allow us to do a subgroup-analysis in which we assess whether the predictive capacity of quantitative fFN is similar in all subgroups.

Health Economic Analysis

An early stage decision-analytic model will be built using evidence from current literature and from the IPD meta-analysis to explore the potential cost-effectiveness of different prognostic models including quantitative fFN.

A literature review will be undertaken to inform model design and identify additional model parameters with searches of Medline, Embase, Cochrane Library and the Paediatric Economic Database Evaluation for economic analyses including the use of fFN testing in woman with threatened preterm labour. Any evidence on resource use (test administration, treatments for preterm labour, hospital stay, hospital transfers, etc), quality of life and diagnostic outcome data from the IPD meta-analysis will be synthesized with the wider evidence based on current practice for women attending hospital with signs and symptoms of preterm labour. The economic analysis will be undertaken from the perspective of the UK NHS adhering to good practice guidelines

For peer review only

and the NICE reference case.[32] A decision tree will be developed to model the clinical pathway. The model will be used to explore potential cost effectiveness of the prognostic model at different thresholds on the Receiver Operator Curve, providing an economic rationale for the chosen prognostic model.

ETHICS AND DISSEMINATION

Trial Management And Oversight Arrangements

Project Management Group

The trial will be coordinated by a Project Management Group (PMG), consisting of the grant holders (Chief Investigator and Co-applicants), the trial manager, representatives from the Study Office and CHaRT (the supporting CTU), plus service user representatives (PAG). The PMG will meet approximately every four months by teleconference or face to face.

Trial Steering Committee and Data Monitoring Committee

A combined Trial Steering Committee and Data Monitoring Committee (TSC/DMC) will oversee the conduct and progress of the study. The terms of reference of the Committee will be developed separately. Members of the TSC/DMC will consist of experts and two patient representatives.

Good Clinical Practice

The study will be conducted in accordance with the principles of Good Clinical Practice (GCP). A favorable ethical opinion has been obtained from the appropriate REC (reference 16/WS/0068) and local R&D approval will be obtained prior to commencement of the study at each site.

Dissemination

On completion of the study, the study data will be analysed and tabulated, and a clinical study report will be prepared. Results will be communicated to the academic

For peer review only - http://bmjopen.bmj.com/site/about/guidelines.xhtml

community via the scientific literature, attendance at conferences and invited presentations. The TRIPOD reporting guidelines will be adhered to.[18] Summaries of results will also be made available to investigators for dissemination within clinics. Social media will be used to signpost publications and conference presentations and highlight important findings. Twitter and Facebook will be used to disseminate findings to professional organizations, charities, stakeholders and the public. Communication to the general public will further be facilitated by our close links with charities such as Tommy's [33].

PEER REVIEW

The study was extensively peer reviewed as part of the process of gaining grant funding from the NIHR HTA (14/32/01).

FUNDING

This project was funded by the National Institute of Healthcare Research Health Technology and Assessment (Reference 14/32/01). The views expressed are those of the authors and not necessarily those of the NHS, the NIHR or the Department of Health.

CONTRIBUTIONS TO AUTHORSHIP

SJS, KB, RKM, JD, LJ, MC, AD, AK, AS, VHM, TL, KK, SHC, BM, RDR, JN and JEN developed the protocol. SJS, LW, RDR, KB, TL and JN drafted the protocol. RKM, JD, LJ, MC, AD, AK, AS, VHM, KK, SHC, BM and JEN reviewed and commented on the protocol.

COMPETING INTERESTS

SJS and JEN work at the University of Edinburgh, who received £1000 sponsorship from Hologic to support a meeting (The Society of Reproductive Investigation and

For peer review only

MRC Centre for Reproductive Health Scientific Symposium on Targeting Inflammation to Improve Reproductive Health across the Lifecourse – August 2017).

AS has in the past (over last five years; not in the last three years) received funding for expenses related to advisory board and internal staff education from Hologic.

MC received sponsorship from Hologic to organise an educational teaching focusing on prediction of Preterm Birth at the 2017 annual meeting of the British Maternal and Fetal Medicine Society.

Hologic, the makers of fFN have provided analysers and technical support for their use to sites participating in the QUIDS prospective cohort study. They have no access to the data, or other involvement in the conduct, analysis, interpretation or decision to publish the results of the study.

REFERENCES

1. Stock SJ, Wotherspoon L, Boyd K et al. Study Protocol: Quantitative Fibronectin to help Decision-making in women with Symptoms of Preterm Labour (QUIDS) Part Two- Prospective Cohort Study. *Submitted to BMJ Open alongside this manuscript(see supplementary information)*

2. Office for National Statistics (2013). ONS gestation-Specific Infant Mortality in England and Wales 2011. Cardiff ONS, 2013. Also available at: https://www.ons.gov.uk/peoplepopulationandcommunity/healthandsocialcare/causesofdeath/bulletins/pregnancyandethnicfactorsinfluencingbirthsandinfantmortality/2013-10-10 (accessed 23rd November 2017).

3. Information Services Division (2013). “Births in Scottish hospitals 2012”. Edinburgh National Services Scotland, 2013. Also available at: http://www.isdscotland.org/Health-Topics/Maternity-and-Births/Publications/2013-08-27/2013-08-27-Births-Report.pdf?34511965514 (accessed 23rd November 2017).

For peer review only

4. Kenyon SL, Taylor DJ, Tarnow-Mordi W and Oracle Collaborative Group. Broad-spectrum antibiotics for spontaneous preterm labour: the ORACLE II randomised trial. ORACLE Collaborative Group. Lancet 2001;357(9261):989-94

5. Peaceman AM, Andrews WW, Thorp JM, et al. Fetal fibronectin as a predictor of preterm birth in patients with symptoms: a multicenter trial. Am J Obstet Gynecol 1997;177(1):13-8

6. Macintyre-Beon C, Skeoch C, Jackson L, Booth P, Cameron A. Scottish Perinatal Collaborative Transport Study. NHS Quality Improvment Scotland 2008

7. Wilson A, MacLean D, Skeoch C, Jackson L. An evaluation of the financial and emotional impact of in utero transfers upon families: a Scotland-wide audit. Infant 2010;6(2):38-40

8. Marlow N, Bennett C, Draper ES, Hennessy EM, Morgan AS, Costeloe KL. Perinatal outcomes for extremely preterm babies in relation to place of birth in England: the EPICure 2 study. Arch Dis Child Fetal Neonatal Ed 2014;99(3):F181-8

9. Asztalos EV, Murphy KE, Willan AR, et al. Multiple Courses of Antenatal Corticosteroids for Preterm Birth Study: Outcomes in Children at 5 Years of Age (MACS-5). JAMA Pediatr 2013

10. de Heus R, Mol BW, Erwich JJ, et al. Adverse drug reactions to tocolytic treatment for preterm labour: prospective cohort study. BMJ 2009;338:b744

11. Yee WH, Sauve R. What information do parents want from the antenatal consultation? Paediatr Child Health 2007;12(3):191-6

12. Honest H, Forbes CA, Duree KH, et al. Screening to prevent spontaneous preterm birth: systematic reviews of accuracy and effectiveness literature with economic modelling. Health Technol Assess 2009;13(43):1-627

13. Deshpande SN, van Asselt AD, Tomini F, et al. Rapid fetal fibronectin testing to predict preterm birth in women with symptoms of premature labour: a

For peer review only

systematic review and cost analysis. Health Technol Assess 2013;17(40):1-138

14. Stock SJ, Morris RK, Chandiramani M et al. Variation in management of women with threatened preterm labour. Arch Dis Child Fetal Neonatal Ed 2015;100(3):F276

15. Debray TP, Riley RD, Rovers MM, Reitsma JB, Moons KG, Cochrane IPDM-aMg. Individual participant data (IPD) meta-analyses of diagnostic and prognostic modeling studies: guidance on their use. PLoS Med 2015;12(10):e1001886

16. Debray TP, Moons KG, Ahmed I, Koffijberg H, Riley RD. A framework for developing, implementing, and evaluating clinical prediction models in an individual participant data meta-analysis. Statistics in medicine 2013;32(18):3158-80

17. Roberts D, Brown J, Medley N, Dalziel SR. Antenatal corticosteroids for accelerating fetal lung maturation for women at risk of preterm birth. Cochrane Database Syst Rev 2017;3:CD004454

18. Collins GS, Reitsma JB, Altman DG, Moons KG. Transparent reporting of a multivariable prediction model for individual prognosis or diagnosis (TRIPOD): the TRIPOD statement. BMJ 2015;350:g7594

19. Lu GC, Goldenberg RL, Cliver SP, Kreaden US, Andrews WW. Vaginal fetal fibronectin levels and spontaneous preterm birth in symptomatic women. Obstet Gynecol 2001;97(2):225-8

20. Gomez R, Romero R, Medina L, et al. Cervicovaginal fibronectin improves the prediction of preterm delivery based on sonographic cervical length in patients with preterm uterine contractions and intact membranes. Am J Obstet Gynecol 2005;192(2):350-9

21. Abbott DH, NL. Seed, PT. Bennett, PR. Chandiramani, M. David, AL. Girling, J, Norman, JE. Stock, SJ. Tribe, RM. Shennan, AH. EQUIPP: Evaluation of

For peer review only

Fetal Fibronectin with a novel bedside Quantitative Instrument for the Prediction of Preterm birth. Arch Dis Child Fetal Neonatal Ed 2014;99:A150-A51

22. Abbott DS, Radford SK, Seed PT, Tribe RM, Shennan AH. Evaluation of a quantitative fetal fibronectin test for spontaneous preterm birth in symptomatic women. Am J Obstet Gynecol 2013;208(2):122 e1-6

23. Bruijn MM, Kamphuis EI, Hoesli IM, et al. The predictive value of quantitative fibronectin testing in combination with cervical length measurement in symptomatic women. Am J Obstet Gynecol 2016;215(6):793 e1-93 e8

24. Bruijn M, Vis JY, Wilms FF, et al. Quantitative fetal fibronectin testing in combination with cervical length measurement in the prediction of spontaneous preterm delivery in symptomatic women. BJOG 2016;123(12):1965-71

25. Whiting PF, Rutjes AW, Westwood ME, et al. QUADAS-2: a revised tool for the quality assessment of diagnostic accuracy studies. Ann Intern Med 2011;155(8):529-36

26. Hayden JA, van der Windt DA, Cartwright JL, Cote P, Bombardier C. Assessing bias in studies of prognostic factors. Ann Intern Med 2013;158(4):280-6

27. Moons KG, de Groot JA, Bouwmeester W, et al. Critical appraisal and data extraction for systematic reviews of prediction modelling studies: the CHARMS checklist. PLoS Med 2014;11(10):e1001744

28. Vergouwe Y, Steyerberg EW, Eijkemans MJ, Habbema JD. Substantial effective sample sizes were required for external validation studies of predictive logistic regression models. J Clin Epidemiol 2005;58(5):475-83

29. Vittinghoff E, McCulloch CE. Relaxing the rule of ten events per variable in logistic and Cox regression. Am J Epidemiol 2007;165(6):710-8

For peer review only

30. Haas DM, Imperiale TF, Kirkpatrick PR, Klein RW, Zollinger TW, Golichowski AM. Tocolytic therapy: a meta-analysis and decision analysis. Obstet Gynecol 2009;113(3):585-94

31. Steyerberg EW, Harrell FE, Jr., Borsboom GJ, Eijkemans MJ, Vergouwe Y, Habbema JD. Internal validation of predictive models: efficiency of some procedures for logistic regression analysis. J Clin Epidemiol 2001;54(8):774-81

32. National Institute for Clinical Excellence (NICE) Guide to the methods of technology appraisal 2013. https://www.nice.org.uk/process/pmg9/chapter/the-reference-case (accessed 23rd November 2017).

33. https://www.tommys.org (accessed 23rd November 2017)

For peer review only

BMJ Open

Study Protocol: Quantitative Fibronectin to help Decision-making in women with Symptoms of Preterm Labour (QUIDS) Part Two- Prospective Cohort Study

Journal:	*BMJ Open*
Manuscript ID	bmjopen-2017-020795.R1
Article Type:	Protocol
Date Submitted by the Author:	07-Feb-2018
Complete List of Authors:	Stock, Sarah; University of Edinburgh MRC Centre for Reproductive Health, Tommy's Centre for Maternal and Fetal Health; University of Western Australia School of Women's and Infant's Health,<br>Wotherspoon, Lisa; University of Edinburgh MRC Centre for Reproductive Health, Tommy's Centre for Maternal and Fetal Health<br>Boyd, Kathleen; University of Glasgow, Health Economics & Health Technology Assessment<br>Morris, R. K.; Univ Birmingham, School of Clinical and Experimental Medicine<br>Dorling, Jon; Queen's Medical Centre, Neonatal Unit<br>Jackson, Lesley; Royal Hospital for Children Glasgow, Neonatal Unit<br>Chandiramani, Manju; Imperial College Healthcare NHS Trust, Queen Charlotte and Chelsea Hospital, Du Cane Road, Shepherds Bush, London, W12 0HS<br>David, Anna; University College London Medical School, Institute for Womens Health<br>Khalil, Asma; St. George's Medical School, University of London<br>Shennan, Andrew; Kings College London, Maternal and Fetal Research Unit<br>Hodgetts Morton, Victoria ; Birmingham Women's Hospital, Metchley Park Road, Edgbaston, ,<br>Lavender, Tina; Manchester University<br>Khan, Khalid; Queen Mary, University of London, Centre for Primary Care and Public Health<br>Harper-Clarke, Susan; PPI Representative<br>Mol, Ben; University of Adelade, The Robinson Institute, School of Paediatrics and Reproductive Health<br>Riley, Richard; Keele University<br>Norrie, John; Edinburgh Clinical Trials Unit, University of Edinburgh No. 9, Bioquarter<br>Norman, Jane; University of Edinburgh
**Primary Subject Heading**:	Obstetrics and gynaecology
Secondary Subject Heading:	Health economics, Diagnostics
Keywords:	Pregnancy, Preterm Birth, Fetal Fibronectin, Cervix, Diagnostic Test

For peer review only

Study Protocol: Quantitative Fibronectin to help Decision-making in women with Symptoms of Preterm Labour (QUIDS) Part Two- Prospective Cohort Study

Sarah J Stock*[1,2] Sarah.Stock@ed.ac.uk
Lisa Wotherspoon[1] Lisa.Wotherspoon@ed.ac.uk
Kathleen Boyd[3] Kathleen.Boyd@glasgow.ac.uk
Rachel K Morris[4] r.k.morris@bham.ac.uk
Jon Dorling[5] Jon.Dorling@nottingham.ac.uk
Lesley Jackson[6] Lesley.Jackson@ggc.scot.nhs.uk
Manju Chandiramani[7] m.chandiramani@imperial.ac.uk
Anna David[8] a.david@ucl.ac.uk
Asma Khalil[9] akhalil@sgul.ac.uk
Andrew Shennan[10] andrew.shennan@kcl.ac.uk
Victoria Hodgetts-Morton[4] v.a.h.morton@bham.ac.uk
Tina Lavender[11] Tina.Lavender@manchester.ac.uk
Khalid Khan[12] k.s.khan@qmul.ac.uk
Susan Harper-Clarke[13] su_clarke@hotmail.com
Ben Mol[14] ben.mol@adelaide.edu.au
Richard D. Riley[15] r.riley@keele.ac.uk
John Norrie[16] J.Norrie@ed.ac.uk
Jane Norman[1] Jane.Norman@ed.ac.uk

1. Tommy's Centre for Maternal and Fetal Health, University of Edinburgh MRC Centre for Reproductive Health, Queen's Medical Research Institute, Edinburgh, UK. 2. School of Women's and Infants' Health, University of Western Australia, Crawley WA 6009, AUS. 3. Institute of Health & Wellbeing Health Economics & Health Technology Assessment, University of Glasgow, 1 Lilybank Gardens, Glasgow, UK. 4. School of Clinical and Experimental Medicine, University of Birmingham, Birmingham, UK. 5. Queen's Medical Centre, Neonatal Unit, Derby Road,

For peer review only

Nottingham, UK. 6. Royal Hospital for Children Glasgow, Neonatal Unit Glasgow, UK. 7. Imperial College Healthcare NHS Trust, Queen Charlotte and Chelsea Hospital, Du Cane Road, Shepherds Bush, London, UK. 8. University College London Medical School, Institute for Women's Health Health, 86-96 Chenies Mews, University College London Medical School London, UK. 9. St. George's Medical School, University of London, London, UK. 10. Kings College London, Maternal and Fetal Research Unit Maternal and Fetal Research Unit 10th Floor, North Wing London, UK. 11. University of Manchester School of Nursing, Midwifery & Social Work, Jean McFarlane Building, University Place, Oxford Road Manchester, UK. 12. Queen Mary, University of London, Centre for Primary Care and Public Health London, UK. 13. Patient and Public Involvement Representative. No affiliation. 14. University of Adelaide, The Robinson Institute, School of Paediatrics and Reproductive Health Adelaide, SA, AUS. 15. Research Institute for Primary Care and Health Sciences, Keele University, Keele, UK. 16. Edinburgh Clinical Trials Unit, University of Edinburgh No. 9, Bioquarter Edinburgh, UK.

*Corresponding Author: Dr Sarah J Stock Senior Clinical Lecturer Maternal and Fetal Medicine, MRC Centre for Reproductive Health, Queen's Medical Research Institute, 47 Little France Crescent, Edinburgh, EH16 4TJ. Tel: 0131 242 6449 Email: sarah.stock@ed.ac.uk

Word Count: 4,928

Key Words: Pregnancy; Preterm Birth; Fetal Fibronectin; Cervix; Diagnostic Test.

For peer review only

ABSTRACT

Introduction: The aim of the QUIDS study is to develop a decision support tool for the management of women with symptoms and signs of preterm labour, based on a validated prognostic model using quantitative fetal Fibronectin (fFN) concentration, in combination with clinical risk factors.

Methods and analysis: The study will evaluate the Rapid fFN 10Q System (Hologic, Malborough, MA) which quantifies fFN in a vaginal swab. In QUIDS Part 2 we will perform a prospective cohort study in at least eight UK consultant-led maternity units, in women with symptoms of preterm labour at 22+0 to 34+6 weeks gestation to externally validate a prognostic model developed in QUIDS Part 1. The effects of quantitative fFN on anxiety will be assessed, and acceptability of the test and prognostic model will be evaluated in a subgroup of women and clinicians (n=30). The sample size is 1600 women (with estimated 96-192 events of preterm delivery within 7 days of testing). Clinicians will be informed of the qualitative fFN result (positive/negative) but be blinded to quantitative fFN result. Research midwives will collect outcome data from the maternal and neonatal clinical records. The final validated prognostic model will be presented as a mobile or web-based application.

Ethics and dissemination: The study is funded by the National Institute of Healthcare Research Health Technology Assessment (HTA 14/32/01). It has been approved by the West of Scotland Research Ethics Committee (16/WS/0068).

Registration details: The study has been registered with ISRCTN Registry (ISRCTN 41598423) and NIHR Portfolio (CPMS: 31277)

Version: Protocol Version 2, Date 1st November 2016

STRENGTHS AND LIMITATIONS OF THIS STUDY

Strengths

- Validation of a prognostic model in a separate prospective cohort study

For peer review only

• Health Economic Analysis to determine cost effectiveness from NHS perspective

Limitations

• Not a randomized control trial to test effectiveness of the model on improved patient outcomes

HOW PATIENTS ARE INVOLVED IN THIS STUDY

Patient representatives were consulted during the protocol development and have been invited to join the Project Management Group and the Trial Steering Committee. Prior to commencing QUIDS, we performed a qualitative study to determine the decisional needs of pregnant women with signs and symptoms of preterm labour, their partners and their caregivers. This is described in the separate protocol “QUIDS Qualitative” (Supplementary Material). The end product of QUIDS will be a decision support aid to help clinicians, women and their partners decide on management of threatened preterm labour, based on the results of the quantitative fFN. In QUIDS Qualitative women and clinicians indicated that they would prefer this to be on web based or mobile app based format, presenting the risk of preterm birth within seven days of testing.

For peer review only

INTRODUCTION

The overall aim of the QUIDS study is to develop a decision support tool for the management of women with symptoms and signs of preterm labour, based on a validated prognostic model using quantitative fFN testing. The study has been conceptually divided into two parts. In this, the protocol for QUIDS Part Two, we detail the protocol for a prospective cohort study. This will externally validate a prognostic model developed in QUIDS Part One.[1] More detailed background about the diagnosis of preterm labour and background to the study is provided in the introduction of QUIDS Protocol Part One.[1]

Fetal Fibronectin (fFN) is a biochemical test of preterm labour which has potential to help improve diagnosis of impending preterm delivery.[2] Much of the evidence about fFN to date relates to the qualitative fFN test, which provides a positive or negative result on the basis of a single threshold of 50ng/ml.[2,3] This test has been largely replaced with the Rapid fFN 10Q System, which provides a concentration of fFN (quantitative fFN), and as a continuous variable, may be a more useful predictor of preterm delivery. fFN is now only available with a quantitative analyser in the UK, but there is no consensus as to which women to use the test in, or how to interpret the results.

The QUIDS study will address this evidence gap by providing evidence about the potential value of the quantitative fFN test, along with guidance about how to interpret results. Here we detail the protocol for external validation of a prognostic model developed in QUIDS Part One.[1]

METHODS AND ANALYSIS

Aims and Methodologies

For peer review only - http://bmjopen.bmj.com/site/about/guidelines.xhtml

The aim of the QUIDS study is to develop a decision support tool for the management of women with symptoms and signs of preterm labour, based on a validated prognostic model using quantitative fFN testing.

The study protocol has been divided into two parts (see flow chart Figure 1). The protocols for Parts One and Two are reported in separate manuscripts.

In QUIDS Protocol Part One we have described how we will perform (i) an Individual Participant Data (IPD) meta-analysis, and (ii) and Economic Analysis. The protocol details how we will develop and internally validate a prognostic model using quantitative fFN (as a continuous variable) and other risk (prognostic) factors and to evaluate the added value of quantitative fFN toward this prognostic model performance. We will also provide an economic rationale for the prognostic model and analyze its cost-effectiveness from the perspective of the NHS.

In this, the QUIDS Protocol Part Two, we will detail the prospective cohort study to externally validate and, if necessary, refine the prognostic model. This will be performed in at least eight UK hospitals with different settings (rural/urban) and different levels of neonatal care facilities. In addition, acceptability of quantitative fFN testing, and effects on maternal anxiety will be performed. We will assess the potential cost-effectiveness of the final prognostic model/decision support tool. This additional analysis will allow us to model the full costs and effect impacts of the different prognostic model and compare these in a cost-effectiveness analysis to provide an evidence-based economic rationale for implementing the diagnostic tool in the NHS.

Endpoints

For peer review only - http://bmjopen.bmj.com/site/about/guidelines.xhtml

The primary endpoint of the prognostic model is spontaneous preterm delivery within seven days of qfFN test, in women less than 36 weeks' gestation. This was influenced by the preceding QUIDS Qualitative Study, which included focus group consultation to determine the decisional needs of women, their partners and clinicians (Supplementary Material). It is also a recognised clinically important endpoint, as antenatal steroids (which significantly reduce morbidity and mortality in preterm babies[4]) are most effective if delivery occurs within seven days of administration.

A secondary endpoint suggested by QUIDS Qualitative Study (Supplementary Material) consultation, was delivery within 48 hours of qfFN test. This analysis will be performed if feasible to do so within the constraints of the data available for model development and validation.[1]

Health technologies being assessed

The trial will evaluate the Rapid fFN 10Q System (Hologic, Malboroughm MA). This provides a concentration of fFN (ng/ml or INVALID) in a vaginal swab sample in 10 minutes. It is now the only commercially available fFN test system, and replaces the TLiQ rapid analyser system, which provided a qualitative fFN result (POSITIVE or NEGATIVE) based on a threshold of 50ng/ml. The Rapid fFN 10Q system is a point of care test, which clinical staff can easily perform. All reagents for fFN testing can be stored at room temperature and specimen collection kits, reagents, cassettes and the 10Q analyzer can be kept in clinical areas where women with symptoms of preterm labour are assessed so they can be conveniently accessed.

Vaginal swab samples are analysed by lateral flow; solid-phase immunochromatographic assay (the Rapid fFN Cassette), and interpreted in the 10Q Rapid analyser. 200 μL of the sample is pipetted into the sample application well of

For peer review only - http://bmjopen.bmj.com/site/about/guidelines.xhtml

the Rapid fFN Cassette using a polypropylene or polyethylene pipette. The sample will then flow from an absorbent pad across a nitrocellulose membrane via capillary action through a reaction zone containing murine monoclonal anti-fetal fibronectin antibody conjugated to blue microspheres (conjugate). The conjugate, embedded in the membrane, will be mobilized by the flow of the sample. The sample will then flow through a zone containing goat polyclonal antihuman fibronectin antibody that captures the fibronectin-conjugate complexes. The remaining sample will flow through a zone containing goat polyclonal anti-mouse IgG antibody that captures unbound conjugate, resulting in a control line. After 10 minutes of reaction time, the intensities of the test line and control line are interpreted with the 10Q Rapid analyser and a printed result provided as a concentration in ng/ml (0->500ng/ml) or INVALID. The result is invalid if the test does not meet internal quality controls that are performed automatically with every test. In the event of an invalid result, the test can be repeated with any remaining clinical specimen. A quality control can be performed by a reusable Rapid fFN 10Q QCette® QC Device, which verifies that the analyser performance is within specification.

Target population

The target population is pregnant women attending hospital with signs and symptoms of preterm labour.

Validation And Refinement Of Prognostic Model

Population

The prospective cohort study will include women with signs and symptoms of preterm labour at 22^{+0} to 34^{+6} weeks gestation in whom admission, transfer or treatment is being considered. These will be recruited from at least eight sites with a mix of rural/urban settings, and have different levels of neonatal care facilities, over 12 months.

For peer review only - http://bmjopen.bmj.com/site/about/guidelines.xhtml

Eligibility Criteria

The following inclusion criteria will apply at screening assessment (all apply):

- Women who are 22^{+0} to 34^{+6} weeks (or earlier gestation if the fetus is considered potentially viable).
- Women showing signs and symptoms of pre-term labour which may include any or all of back pain, abdominal cramping, abdominal pain, light vaginal bleeding, vaginal pressure, uterine tightenings or contractions.
- Women where hospital admission, interhospital transfer or treatment (antenatal steroids, tocolysis or magnesium sulphate) is being considered due to signs of pre-term labour.
- Women aged 16 years or above.

The broad inclusion criteria reflect current clinical practice and enable the generalisability of the results of the trial for routine clinical care. We will include women who re-attend seven days or more after initial recruitment with signs and symptoms of preterm labour and also women who remain symptomatic but undelivered seven days later in whom repeat testing by the clinician is deemed to be appropriate. This will be in line with manufacturer's recommendation for fFN testing.

The following inclusion criteria will apply on speculum examination:

- Cervical dilation ≤ 3cm
- Intact membranes
- No significant vaginal bleeding, as judged by the clinician.
- Once it has been established that the women meets the above criteria, on speculum examination, the fFN swab can be taken.

Participants that sign the consent but are not eligible upon examination to have an fFN swab taken will still be enrolled and have outcome data collected.

For peer review only

The following exclusion criteria will apply:

- Contraindication to vaginal examination (e.g. placenta praevia).
- Higher order multiple pregnancy (triplets or more).
- Moderate or severe vaginal bleeding.
- Cervical dilatation greater than 3cm.
- Confirmed rupture of membranes.
- Sexual intercourse, vaginal examination or transvaginal ultrasound in the preceding 24 hours factors may invalidate results. These women will be initially excluded from the study, but can be included if still symptomatic after 24 hours, when fFN accuracy will be restored.

Co-Enrolment

This trial involves validating a decision support tool relating to a test that is currently commonly used in clinical practice. As such, there are no additional interventions. Co-enrolment in other non-interventional trials will be allowed. Co-enrolment in trials of tocolytic treatments or other management strategies that may influence timing of delivery as a primary outcome will not be allowed. Participation in QUIDs would not preclude babies being subsequently involved in interventional trials. Co-enrolment will be recorded in the electronic case report form (eCRF).

Setting

The prospective cohort study will take place in at least eight consultant-led obstetric units in the UK. More than 93% of pregnant women in the UK deliver in consultant-led units.[5,6] The vast majority of women with symptoms of preterm labour will present to a consultant-led unit for assessment, either directly or following advice from their community midwife or General Practitioner.

For peer review only

The study will not include any community maternity units (staffed by midwives, with or without involvement of non-obstetric medical staff), which cover a small proportion of women, mainly in remote and rural areas. In the Perinatal Collaborative Transport Study (CoTS study) of perinatal transfers in Scotland,[7] which involved 52,727 births, only 69 (0.13%) women were transferred to a consultant-led obstetric unit from community maternity units, and only a proportion of these were for suspected preterm labour. The small number of women cared for in community maternity units means their inclusion would not be an efficient use of study resources.

Given that management of women with symptoms of preterm labour and inter-hospital transfer patterns are likely to vary depending on level of available neonatal care and distance to transfer, we will include a mixture of hospitals with different levels of neonatal care facilities in both rural and urban settings. We will include units with Special Care Units (providing special care for their own local population), Local Neonatal Units (providing special care and high dependency care and a restricted volume of intensive care) and Neonatal Intensive Care Units (larger intensive care units providing the whole range of medical, and sometimes surgical neonatal care for their local population and for babies and their families referred from the neonatal network in which they are based, and other networks when necessary). The hospitals will be chosen from different geographical settings (rural/urban) and from different regions of the UK.

If additional units wish to participate in the study we will consider including them, to increase recruitment rates. The UK Reproductive Health and Childbirth specialty group (clinical study group) have contributed to the study protocol and support the proposed trial.

For peer review only

Participant Selection And Enrolment

Women with signs and symptoms of preterm labour will be identified on presentation to obstetric services. A member of clinical staff, usually the doctor or midwife assessing the woman, will identify potentially eligible participants, provide a participant information leaflet and invite consent. A suitably trained member of clinical staff (doctor or midwife) or research team will consent participants.

Posters and leaflets will be situated in antenatal areas of participating hospitals to alert women that the study is taking place, and women will be allowed as much time as possible to consider participation without unduly delaying further clinical assessment. Participants will receive adequate oral and written information and appropriate participant information and informed consent forms will be provided.

Screening For Eligibility

The clinical likelihood of preterm delivery is usually evaluated by history and examination, which includes abdominal palpation, to assess strength and frequency of uterine contractions. If preterm labour is suspected, a vaginal speculum examination is performed where the cervix is inspected for dilatation, and evidence of vaginal bleeding and membrane rupture assessed. Swabs for fFN are usually taken at this point. Potential participants in the QUIDS study will be identified after the initial assessment and provided with information about the study. A combined 'Screening and Consent Form' will be used as a self-screening tool for potentially eligible participants. Informed consent will take place before speculum examination and the fFN swab has been taken. This approach means that samples are collected at routine speculum examination, as they would be if fFN is implemented in clinical practice, and participants avoid an additional vaginal examination.

Ineligible And Non-Recruited Participants

For peer review only - http://bmjopen.bmj.com/site/about/guidelines.xhtml

Certain exclusion criteria can only be assessed at speculum examination (for example vaginal bleeding or evidence of ruptured membranes), so a proportion of women will not be eligible for fFN testing after consent is given. These women will still be enrolled and delivery outcomes collected. The decision whether to use this data for analysis will be the decision of the Chief Investigator and Statisticians.

Withdrawal Of Study Participants

Women will be able to withdraw consent for us of their data at any time until the end of the study.

Study Assessments (See Table 1)

Eligibility Assessment (Screening And Recruitment)

Women presenting with signs and symptoms of pre-term labour will be identified on presentation to obstetric services. The doctor or midwife assessing the woman will identify potentially eligible participants and provide an invitation letter and short information leaflet.

After the woman has had the opportunity to consider whether she would like to participate, she will be asked to complete the Screening and Consent Form. The clinician will then decide whether the fFN test can be carried out. If the test can be carried out (according to manufacturer’s guidelines), then the participant will be fully enrolled and that their delivery outcomes will still be collected.

If the woman declines to participate and she is willing to provide a reason for this, the reason given will be entered on to an anonymous log. Baseline demographics will be collected on consenting women, together with height and weight, information on medical history, obstetric history, estimated date of delivery and presenting signs and symptoms.

For peer review only - http://bmjopen.bmj.com/site/about/guidelines.xhtml

The original consent form will be stored in the Investigator Site File (ISF) file, a copy is given to the woman, a copy added to the medical notes and a copy sent to the Trial Office.

After providing consent, the participant will be asked to complete a short State Trait Anxiety Inventory (STAI) questionnaire and complete a contact details form. They will also be issued with a letter thanking them for taking part in the trial and giving details of the second questionnaire to be completed.

Sample Collection

Samples for analysis will be taken with a fFN specimen collection kit, which consists of a sterile polyester tipped swab and a specimen transport tube containing 1 ml extraction buffer (an aqueous solution containing protease inhibitors and protein preservatives including aprotinin, bovine serum albumin, and sodium azide). During speculum examination the sterile swab will be lightly rotated across the posterior fornix of the vagina for ten seconds to absorb vaginal secretions. Samples should be taken before any other swabs (e.g. for microbiology) or cervical manipulation and the speculum lubricated with normal saline as other lubricants may interfere with the antibody-antigen reaction of the test. Following specimen collection the swab should be removed, immersed in extraction buffer, the shaft of the swab snapped off, and the transport tube sealed.

Before analysis samples are gently mixed and as much liquid as possible expressed from the swab by rolling the tip against the inside of the tube.

Initial fFN test

For peer review only

The sample taken will be run at a near bedside Hologic Rapid fFN 10Q analyser, specially adapted for the QUIDS study. As fFN (or other similar biochemical tests of preterm labour) are part of standard care, it would be unethical to blind clinicians from the qualitative fFN result. The analyser will thus reveal a qualitative fFN result (positive/negative/invalid) for clinicians to base clinical decision-making on, according to local protocols. The quantitative fFN result however, will be stored as a three-letter code, blinding caregivers from the result. Samples will be run as per manufacturers instructions (described above in the section "Health technologies being assessed").

Repeat fFN Tests

If there is clinical indication for further fFN tests (eg because of ongoing symptoms of preterm labour after seven days), the results will also be recorded.

Labour/Delivery/ Neonatal Assessments

Admission for delivery will not be a formal study visit but data will be collected using information recorded in the participant's notes. Delivery data will be collected on the maternal outcomes of delivery, including method of delivery, indication for delivery method, onset of labour, date and gestation of delivery and blood loss.

Questionnaires

All participants who are eligible to participate will be asked to complete a STAI questionnaire before the speculum examination. The same questionnaire will be repeated 24-48 hours post examination. The second questionnaire will be provided on paper with a pre-paid envelope to be returned by post to the Trial Office. If not returned by post, the Trial Office may try to contact the participant (with the contact details provided), to complete the questionnaire over the phone.

For peer review only

	Attendance with signs and symptoms preterm labour			
**Visit**	**Screening and Recruitment**	**24-48h**	**1-6 months**	**DELIVERY**
Inclusion/Exclusion Criteria	⊙			
Participant Information Sheet	⊙			
Consent Form	⊙			
Demographics	⊙			
Obstetric History	⊙			
Symptoms and Signs	⊙			
Quantitative fFN (concentration ng/ml)	⊙			
Cervical length scan (if available)	⊙			
State Trait Anxiety Inventory Questionnaire	⊙	⊙		
Delivery details				⊙
Neonatal outcomes				⊙
Qualitative Acceptability Questionnaires (subgroup n=30)			⊙	

Table 1: QUIDS Study Assessments

For peer review only

Safety and Quality Assessments

The Hologic Rapid fFN 10Q analyzer has integrated quality control measures, and we will keep records of these as well as any additional staff training that occurs after the study starts. It is recommended that a daily pre-calibrated reusable quality control cassette be inserted and analysed every 24 hours to verify that the analyser performance is within specification. A daily quality control (QC) should be performed if one has not been done in the preceding 24 hours before a patient test is to be done. Logs of results are stored on the machine and can be downloaded, and we will also ask the participating sites to keep a monthly paper log of QC tests done. Each patient test has an internal quality control, with a procedural control line that verifies the threshold level of signal by the instrument. Sample flow detection ensures the sample travels across the cassette properly, and confirms absence of conjugate aggregation. We believe that these measures will help ensure the validity of results. However, to provide further evidence of integrity and comparability of results from each site we will request that all participating sites enrol in the Wales External Quality Assurance Scheme (WEQAS) Point of Care Quality Assurance Scheme. WEQAS will provide a sample for analysis to each site bimonthly, and provide reports on analyser performance and variability.[8]

Data Collection

Data For Prognostic Model Validation and Update of Health Economic Model

We will collect data on all of the candidate predictors considered for inclusion in the prognostic model developed in the IPD meta-analysis (quantitative fFN concentration, previous spontaneous preterm labour, gestation at fFN test, age, ethnicity, BMI, smoking, deprivation index, number of uterine contractions in set time period, cervical dilatation, vaginal bleeding, previous cervical treatment for cervical intraepithelial neoplasia, cervical length [measured by transvaginal cervical length; when available], singleton/multiple pregnancy, tocolysis and fetal sex). Outcome data will include gestational age at delivery, date and time of delivery, administration of

For peer review only - http://bmjopen.bmj.com/site/about/guidelines.xhtml

treatments for preterm labour (steroids, antibiotics, tocolysis, magnesium sulphate) duration hospital admission, hospital transfer, onset of labour (preterm prelabour rupture of membranes; idiopathic preterm birth; medically indicated preterm birth [and indication]), place of delivery (base hospital, other hospital, outwith hospital), mode of delivery, neonatal admission, neonatal complications, perinatal mortality, congenital anomaly, sex and birthweight.

Screening data and data about quantitative fFN testing will be collected on paper based CRFs and research midwives will input these into the web based electronic database. Clinical outcome data will be collected from the medical records.

Maternal Acceptability and Anxiety

Maternal anxiety will be measured pre and post-test (24-48h) using the validated State Trait Anxiety Inventory (STAI) questionnaire. Acceptability of fFN testing and the decision support will be assessed using follow up interviews (face to face or telephone, according to maternal preference) which will be conducted with a sub-group of participants (n=30) purposively sampled and stratified according to geographical location, outcome (preterm labour or not) and anxiety scores. Acceptability will also be assessed in a cohort of clinicians (n=30).

Statistics and Sample Size Calculation

Guidance for external validation suggests at least ten events (preterm delivery within seven days of test) are required for each covariate included in a prognostic model.[9,10] Data from the cohorts included in our IPD meta-analysis suggests an event rate of between 6 and 12%.[1] Based on these estimates a sample size of 1,600 will provide 96 and 192 events (preterm delivery within 7 days).

A UK study has shown that 8.9% of pregnant women present with symptoms of preterm labour and are eligible for quantitative fFN[11] and we anticipate 50%

For peer review only - http://bmjopen.bmj.com/site/about/guidelines.xhtml

recruitment rate is achievable, thus overall 4.5% of maternities could be recruited. We will initially include eight units in the cohort study with a combined delivery rate of approximately 36,000 per annum. We anticipate that we will achieve target recruitment within 12 months (1 year * 36,000 * 0.089 * 0.5 = 1,602). If however, the recruitment rate or event rate is lower than predicted, we will increase the number of sites included in the study and/or the recruitment period, to ensure that a minimum of 60 events (preterm delivery within 7 days of test) are achieved, allowing for external validation of at least six covariates in our model.

It is possible that the IPD meta-analysis will find there is potential added value of combining quantitative fFN testing with cervical length measurement.[12,13] As cervical length measurement has significant resource requirement (estimated NHS cost £68.16 per test) and lack of out of hours provision further limits availability in many NHS hospitals, we think it is very unlikely that cervical length scanning will improve performance of the prognostic model to such a degree as to make it cost effective. We will assess the incremental costs and effects of cervical length measurement in the proposed health economic model performed in parallel with the IPD meta-analysis, and will feed into design considerations during the first iteration of the prognostic model.

If inclusion of cervical length ultrasound is found to be potentially cost-effective, we will assess the feasibility of including it in the prospective cohort study. We anticipate that including cervical length measurement in the prospective cohort study would be extremely difficult in the current NHS setting as the majority of units do not have 24 hour availability of transvaginal ultrasound and/or trained personnel to perform scans. Inclusion of cervical length would also likely decrease recruitment rate (due to need for additional transvaginal ultrasound examination) and require significant additional resources.

For peer review only - http://bmjopen.bmj.com/site/about/guidelines.xhtml

Analysis

Validation Of Prognostic Model

The prognostic model developed in the IPD will be externally validated using data collected in the prospective cohort data, using the measures of discrimination and calibration described in QUIDS Protocol Part One,[1] including R^2, C statistic, calibration slope, calibration-in-the-large, and calibration plots of observed versus predicted risks across deciles (with Loess smoother). The average performance of the model will be summarised across the centers in the cohort study. Between-center heterogeneity in performance will also be summarised, and reduced (if necessary) by recalibration techniques regarding the strategy for the choice of baseline risk (intercept). That is, the predictor effects will not be modified from the IPD meta-analysis model, but the intercept may need to be tailored to improve validation in UK centers (e.g. for rural settings). Based on the findings, a final model and its implementation strategy will then be recommended for use.

Economic Analysis

The economic model will be refined, integrated and updated with data from the prospective study cohort, so as the most up to date and validated evidence is used to inform a cost-effectiveness decision. Such an iterative approach to economic evaluation is now well established.[14,15] The care pathway following diagnosis will be included in the economic analysis, using data from the cohort study such as the diagnostic test accuracy data, resource use data (i.e. steroid use, other medications, time in hospital, hospital transfer) and secondary outcome data (i.e., treatment of side-effects, morbidity, mortality) so as to capture the full costs and effect impacts (quality of life, morbidity and mortality) for both the mother and baby. Resource use data will be combined with unit cost information from the British National Formulary[16] and NHS reference costs.[17,18] Outcomes will be reported as the incremental cost per correct diagnosis, and incremental cost per Quality Adjusted Life Year (QALY) gained of the qfFN prognostic model compared to current practice

For peer review only

(no qualitative fFN model). The analysis will adhere to the NICE reference case and the recommended guidelines for decision modeling and reporting of economic analyses.[18] Probabilistic sensitivity analysis will be undertaken to explore how uncertainty in the model inputs impact on the cost-effectiveness outcome.[19]

Acceptability of fFN Testing and Effects on Anxiety

Maternal anxiety will be measured before and after quantitative fFN testing using the validated STAI. The STAI Form Y is a widely used tool for measuring both temporary "state anxiety" and the more general, long-standing "trait anxiety". The STAI is designed for the self-reported assessment of the intensity of feelings of apprehension, tension, nervousness, and worry. STAI-Anxiety scores increase in response to physical danger and psychological stress, making it highly appropriate for this study. The use of STAI in pregnancy studies is discussed by Hundley, et al and we will interpret the results accordingly.[20]

The questionnaire will be administered prior to fFN testing (baseline) and 24-48 hours after the test, to assess early reactions to the test and any acute anxiety prompted by the result of the test. We will also be able to assess any differences in those presented with a high risk or low risk result. Although it might be interesting to assess anxiety again in the latter stages of pregnancy, it is likely that, in this population, many pregnancies will not reach full term. Thus we believe our strategy of repeat questionnaire administration will allow measurement of longer term anxiety induced or alleviated by the test, whilst minimising bias due to preterm or term delivery itself or loss to follow up.

Follow up interviews will be performed with a sub-group of participants (n=30) to enable deeper exploration of women's views regarding fFN testing, to gain insight into the rationale for responses given in the questionnaires. Interviews will be conducted following confirmation of pregnancy status. Acceptability of the prognostic

For peer review only - http://bmjopen.bmj.com/site/about/guidelines.xhtml

model will also be assessed with women and a group of clinicians. All interviews will be audio recorded with consent, and field notes taken to ensure an audit trail.

Decision Support

We will develop a decision support tool in accordance with the guidelines produced by the International Patient Decision Aid Standards (IPDAS) Collaboration.[21] Scoping of decisional requirements and how data should be presented was performed during focus group consultation as part of QUIDS Qualitative (Supplementary Material). A prototype decision support tool incorporating the initial prognostic model developed as part of the IPD-meta-analysis, will be tested with women and clinicians, as part of the acceptability studies described above. A final version will be updated with the validated (and, if necessary revised) prognostic model generated from the prospective cohort study. The multidisciplinary trial steering committee will oversee the development process, and decide how material is selected for inclusion.

ETHICS AND DISSEMINATION

Trial Management And Oversight Arrangements

Project Management Group

The trial will be coordinated by a Project Management Group (PMG), consisting of the grant holders (Chief Investigator and Co-applicants), the trial manager, representatives from the Study Office and CHaRT (the supporting CTU), plus service user representatives (PAG). The PMG will meet approximately every four months by teleconference or face to face.

The Trial Manager based in Edinburgh will oversee the study and will be accountable to the Chief Investigator. The Trial Manager supported by the trial administrator(s) will take responsibility for the day-to-day transaction of study activities. They will be supported by the CTU at CHaRT to provide expertise and guidance. The Trial

For peer review only - http://bmjopen.bmj.com/site/about/guidelines.xhtml

Manager will be responsible for checking the CRFs for completeness, plausibility and consistency. Any queries will be resolved by the Investigator or delegated member of the trial team.

A Delegation Log will be prepared for each site, detailing the responsibilities of each member of staff working on the trial.

Trial Steering Committee and Data Monitoring Committee

A combined Trial Steering Committee and Data Monitoring Committee (TSC/DMC) will oversee the conduct and progress of the trial. The terms of reference of the Committee will be developed separately. Members of the TSC/DMC will consist of experts and two patient representatives.

Good Clinical Practice

The study will be conducted in accordance with the principles of Good Clinical Practice (GCP). A favorable ethical opinion has been obtained from the appropriate REC (reference 16/WS/0068) and local R&D approval will be obtained prior to commencement of the study at each site.

Dissemination

On completion of the study, the study data will be analysed and tabulated, and a clinical study report will be prepared in accordance with GCP guidelines. Results will be communicated to the academic community via the scientific literature, attendance at conferences and invited presentations. Summaries of results will also be made available to investigators for dissemination within clinics. Social media will be used to signpost publications and conference presentations and highlight important findings. Twitter and Facebook will be used to disseminate findings to professional organizations, charities, stakeholders and the public. Communication to the general public will further be facilitated by our close links with charities such as Tommy's.[22]

For peer review only - http://bmjopen.bmj.com/site/about/guidelines.xhtml

For peer review only

We anticipate that the decision support will be made available as web based application that will be made freely available so clinicians can access it easily and it can be readily translatable into UK practice. If it is found to be effective in ruling out preterm delivery, it is likely that it will decrease unnecessary costly, and potentially harmful treatments in women who have symptoms suggestive of preterm labour but do not deliver early.

PEER REVIEW

The study was extensively peer reviewed as part of the process of gaining grant funding from the NIHR HTA (14/32/01).

FUNDING

This project was funded by the National Institute of Healthcare Research Health Technology and Assessment (Reference 14/32/01). The views expressed are those of the authors and not necessarily those of the NHS, the NIHR or the Department of Health.

CONTRIBUTIONS TO AUTHORSHIP

SJS, KB, RKM, JD, LJ, MC, AD, AK, AS, VHM, TL, KK, SHC, BM, RDR, JN and JEN developed the protocol. SJS, LW, RDR, KB, TL and JN drafted the protocol. RKM, JD, LJ, MC, AD, AK, AS, VHM, KK, SHC, BM and JEN reviewed and commented on the protocol.

COMPETING INTERESTS

SJS and JEN work at the University of Edinburgh, who received £1000 sponsorship from Hologic to support a meeting (The Society of Reproductive Investigation and

For peer review only - http://bmjopen.bmj.com/site/about/guidelines.xhtml

MRC Centre for Reproductive Health Scientific Symposium on Targeting Inflammation to Improve Reproductive Health across the Lifecourse – August 2017).

AS has in the past (over last five years; not in the last three years) received funding for expenses related to advisory board and internal staff education from Hologic.

MC received sponsorship from Hologic to organise an educational teaching focusing on prediction of Preterm Birth at the 2017 annual meeting of the British Maternal and Fetal Medicine Society.

Hologic, the makers of fFN have provided analysers and technical support for their use to sites participating in the QUIDS prospective cohort study. They have no access to the data, or other involvement in the conduct, data analysis, interpretation of results or decision to publish the results of the study.

REFERENCES

1. Stock SJ, Wotherspoon L, Boyd K et al. Study Protocol: Quantitative Fibronectin to help Decision-making in women with Symptoms of Preterm Labour (QUIDS) Part One- Individual Patient Data Meta-analysis and Health Economic Analysis. *Submitted to BMJ Open alongside this manuscript(see supplementary information)*

2. Honest H, Forbes CA, Duree KH, et al. Screening to prevent spontaneous preterm birth: systematic reviews of accuracy and effectiveness literature with economic modelling. Health Technol Assess 2009;13(43):1-627

3. Deshpande SN, van Asselt AD, Tomini F, et al. Rapid fetal fibronectin testing to predict preterm birth in women with symptoms of premature labour: a systematic review and cost analysis. Health Technol Assess 2013;17(40):1-138

4. Roberts D, Brown J, Medley N, Dalziel SR. Antenatal corticosteroids for accelerating fetal lung maturation for women at risk of preterm birth. Cochrane Database Syst Rev 2017;3:CD004454

For peer review only

5. Office for National Statistics (2013). ONS gestation-Specific Infant Mortality in England and Wales 2011. Cardiff ONS, 2013. Also available at: https://www.ons.gov.uk/peoplepopulationandcommunity/healthandsocialcare/causesofdeath/bulletins/pregnancyandethnicfactorsinfluencingbirthsandinfantmortality/2013-10-10 (accessed 23rd November 2017).

6. Information Services Division (2013). "Births in Scottish hospitals 2012". Edinburgh National Services Scotland, 2013. Also available at: http://www.isdscotland.org/Health-Topics/Maternity-and-Births/Publications/2013-08-27/2013-08-27-Births-Report.pdf?34511965514 (accessed 23rd November 2017).

7. Macintyre-Beon C, Skeoch C, Jackson L, Booth P, Cameron A. Scottish Perinatal Collaborative Transport Study. NHS Quality Improvment Scotland 2008

8. http://www.weqas.com/services/poct-eqa/ (accessed 23rd November 2017).

9. Vergouwe Y, Steyerberg EW, Eijkemans MJ, Habbema JD. Substantial effective sample sizes were required for external validation studies of predictive logistic regression models. J Clin Epidemiol 2005;58(5):475-83

10. Vittinghoff E, McCulloch CE. Relaxing the rule of ten events per variable in logistic and Cox regression. Am J Epidemiol 2007;165(6):710-8

11. Abbott DH, Seed PT, Bennett PR et al. EQUIPP: Evaluation of Fetal Fibronectin with a novel bedside Quantitative Instrument for the Prediction of Preterm birth. Arch Dis Child Fetal Neonatal Ed 2014;99:A150-A51

12. van Baaren GJ, Vis JY, Wilms FF, et al. Predictive value of cervical length measurement and fibronectin testing in threatened preterm labor. Obstet Gynecol 2014;123(6):1185-92

13. DeFranco EA, Lewis DF, Odibo AO. Improving the screening accuracy for preterm labor: is the combination of fetal fibronectin and cervical length in symptomatic patients a useful predictor of preterm birth? A systematic review. Am J Obstet Gynecol 2013;208(3):233 e1-6

For peer review only

14. Ades AE, Sculpher M, Sutton A, et al. Bayesian methods for evidence synthesis in cost-effectiveness analysis. Pharmacoeconomics 2006;24(1):1-19

15. Sculpher MJ, Claxton K, Drummond M, McCabe C. Whither trial-based economic evaluation for health care decision making? Health Econom 2006;15(7):677-87

16. British National Formulary. BNF 73. 2017. .

17. Department of Health. Information Services Division. Scottish Health Service Costs Book 2012. 2012. Information Services Division.

18. National Institute for Health and Care Excellence. Guide to the Methods of Technology Appraisal: 2013. 2013 National Institute for Health and Clinical Excellence.

19. Briggs AH. Handling uncertainty in cost-effectiveness models. Pharmacoeconomics 2000;17(5):479-500

20. Hundley V, Gurney E, Graham W, Rennie AM. Can anxiety in pregnant women be measured using the State-Trait Anxiety Inventory. Midwifery 1998;14(2):118-21

21. http://ipdas.ohri.ca (accessed 23rd November 2017)

22. https://www.tommys.org (accessed 23rd November 2017)

Figure Legends

Figure 1

Flow chart illustrating the design of QUIDS study and conceptual division into Part 1 and Part 2

For peer review only

Figure 1

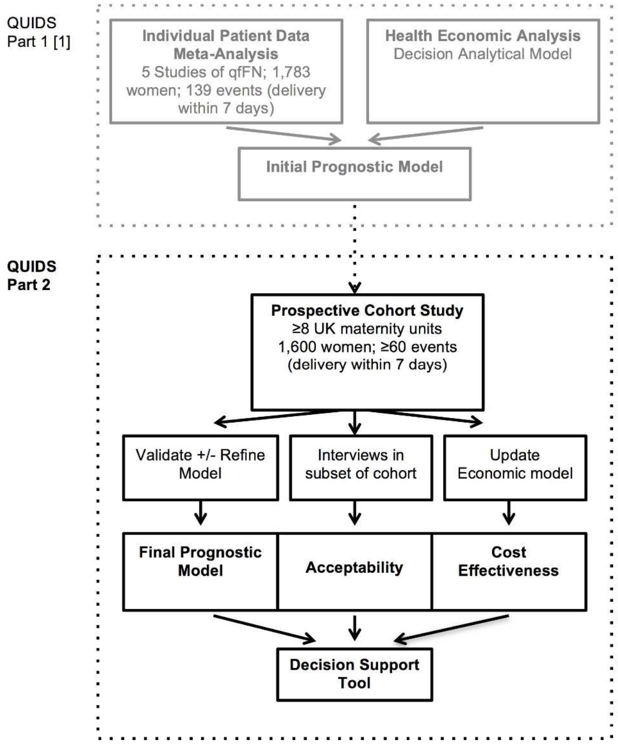

209x297mm (300 x 300 DPI)

QUIDS Qualitative

Quantitative Fibronectin to help Decision-making in women with Symptoms of Preterm Labour:

determining decisional requirements

Protocol

(October 2015)

White H, O'Brien E, Hodgetts-Moreton V, Stock S, Lavender T

For peer review only

Background

Preterm birth, defined as birth prior to 37 weeks gestation, occurs in 6-7% of pregnancies in Europe[1] and was recorded as 5.78% in England in 2013/14, equating to over 37,000 births.[2] Preterm birth is associated with a high risk of mortality, wide-ranging short- and long-term morbidities,[3,4] and significant economic costs to the NHS compared with birth at term.[5] Reducing the detrimental impact of preterm birth relies on the provision of timely and appropriate perinatal interventions. However, accurate prediction of preterm birth is challenging, even when the clinical symptoms are suggestive of preterm labour. In randomised trials approximately 80% of women diagnosed with preterm labour remained pregnant after 7 days.[6,7]

Interventions in preterm labour and preparations for preterm birth may include administration of corticosteroids to accelerate fetal lung maturation[8,9] and magnesium sulphate for fetal neuroprotection,[10] in utero transfer to a facility with appropriate maternity and neonatal services, and tocolysis to optimise time before birth to enable these.[11] Whilst such interventions can improve outcomes for mothers and babies who do experience preterm birth, they are not necessarily benign, especially for those in whom preterm birth does not occur.

The maximal beneficial impact of corticosteroids occurs with administration between 48 hours and seven days before birth, thus timing is especially important in optimising benefit for the neonate. For women who remain at risk of preterm birth after seven days of the initial dose, repeated doses reduce respiratory distress in the neonate[9] but have been found to be associated with a dose-dependent reduction in birthweight.[12,13] A five-year follow-up study of women who received repeated doses of antenatal corticosteroids due to risk of preterm birth found an increased risk of neurodevelopment impairment in infants born at term.[14] Therefore developing a strategy to establish the optimal time to give steroids is a research priority.

For peer review only

For peer review only - http://bmjopen.bmj.com/site/about/guidelines.xhtml

Magnesium sulphate administration immediately prior to birth has been shown to reduce cerebral palsy,[10] but there is a risk of magnesium toxicity leading to respiratory depression in the mother and, theoretically, the neonate.[15]

Whilst there is no clear beneficial effect of tocolytics on the incidence or outcome of preterm birth,[16] their use is recommended if the days gained prior to preterm birth can be used appropriately, for example transfer to a suitable maternity unit or the administration of drugs to protect the neonate.[11] Tocolysis is linked with various maternal and neonatal complications,[17] hence the need for therapy targeted only for those at risk of preterm birth and close monitoring of the mother and fetus throughout.

Often, inpatient admission is recommended if preterm labour is suspected. Previous literature has highlighted the social isolation and support needs that women with high-risk pregnancies who are hospitalised experience.[18] In some cases, in-utero transfer is indicated to ensure that birth takes place in a specialist unit with appropriate neonatal care facilities. This policy has been shown to reduce mortality[19,20] and morbidity[21] in preterm neonates, especially those born very premature. Qualitative research has indicated that women generally acknowledge the potential benefit of in utero transfer to their baby and, hence, are willing to endure the inconvenience and upheaval that it entails.[22,23] However, the experience is associated with an emotional, social and financial burden on women and their families, especially for the substantial proportion of women who do not deliver prematurely following in utero transfer. When describing their experiences of in utero transfer, women expressed shock at the prospect of the transfer, feeling socially isolated, and having no control over the situation, in addition to the practical difficulties experienced particularly by women who already had children.[22,24,25] In a large survey of women who had experienced in utero transfer, over a quarter lamented the financial cost[24] particularly with respect to their partner's outlay for travel, food, accommodation, and phone bills, exacerbated with requiring time off work.[22] Furthermore, in utero transfer is costly to maternity services. Securing a maternal and neonatal bed

V 1.3
21/10/15

For peer review only - http://bmjopen.bmj.com/site/about/guidelines.xhtml

in another unit is a time-consuming task that often falls to delivery suite midwives to arrange, whilst also continuing to provide care to the woman.[26] In a large observational study of all in utero transfers that took place in Scotland in a six-month period, nearly one third of all transfers were due to threatened preterm labour.[27] Under half of the women transferred from one consultant-led unit to another gave birth within 48 hours.[27] Such unnecessary transfers are costly to women, their families and maternity services. Qualitative research into women's experiences of preterm labour have highlighted the need for caregivers to create an environment where women are enabled to discuss their fears[28] and exert control over how they manage their preterm labour care.[25]

Accurate prediction of preterm birth could reduce the burdens and risks associated with unnecessary interventions, and enable women and their clinicians to make informed decisions regarding their care. Numerous diagnostic tests have been used in preterm labour, including biochemical tests of vaginal secretions and cervical length.[29] One such test is fetal fibronectin, a near-bedside test that provides a positive or negative result and has excellent negative predictive value.[30] Thus fetal fibronectin can identify which women will not benefit and may be put at risk by the interventions described previously, and reduce costs to maternity services.[31] Developments in fetal fibronectin testing have led to a quantitative test that provides a concentration of fetal fibronectin in vaginal secretions, giving women and clinicians more information on which to base their management decisions.[32]

Qualitative evidence has indicated that women feel a sense of increased responsibility to their babies and themselves during a high risk pregnancy, such as threatened preterm labour.[33] Women want to be involved in decision making about their care to different degrees and feel most satisfied when their caregiver supports them to make decisions in the way they felt most comfortable.[33]

For peer review only - http://bmjopen.bmj.com/site/about/guidelines.xhtml

Previous literature on decision making and preterm birth has focussed on diagnostic tests[6,28–32,34] and the care of the preterm infant.[35,36] To date, there has been no investigation of what women, their partners and caregivers would like to know in order to make informed decisions about the care that is provided following the signs and symptoms of preterm labour.

Funding has been received from the National Institute for Health Research Health Technology Assessment Programme for a large, multicentre trial to develop a mobile application decision support tool for the management of women with symptoms and signs of preterm labour, based on a validated model using quantitative fetal fibronectin testing. This study is the precursor to that trial, with the aim of determining the decisional needs of pregnant women with the symptoms and signs of preterm labour, their families and caregivers, using a qualitative framework approach. The outcomes of this qualitative study will inform the development of the mobile application decision support tool, using the findings from an individual patient data meta-analysis. The tool will then be externally validated and refined in the multi-centre trial, QUIDS.

For peer review only

For peer review only - http://bmjopen.bmj.com/site/about/guidelines.xhtml

Methods

A qualitative framework approach will be used, based on data collected from focus groups and semi-structured telephone interviews.

Setting

Focus groups will take place in three maternity units: Liverpool Women's NHS Foundation Trust, Birmingham Women's NHS Foundation Trust and Royal Edinburgh Hospital, NHS Lothian. There will be focus groups for women and a separate focus group for partners. Clinicians who care for women with threatened preterm birth will be interviewed by telephone.

Sample

A purposive sample of women and partners will be recruited to cover a variety of experiences of preterm labour and birth. Women will be stratified by their prior experience and relevant characteristics, including ethnicity, previous obstetric history, living in an urban or rural setting and proximity to a tertiary neonatal referral centre. Two focus groups of 4–8 women will be conducted at each site; one for pregnant women who are at high risk of preterm birth, and one for postnatal women who have recently experienced preterm birth. One partners' focus group will be conducted at one of the sites. If women or partners are unable to attend a focus group but still wish to participate, a semi-structured telephone interview will be offered.

Up to 10 obstetricians, including trainees, midwives, and neonatologists will be purposefully recruited to cover a range of professional backgrounds and experience. Semi-structured telephone interviews will be used to collect the data.

For peer review only

For peer review only - http://bmjopen.bmj.com/site/about/guidelines.xhtml

Eligibility

Principal inclusion criteria for women's antenatal focus groups

Women who are currently pregnant who:

- Have previously experienced preterm birth following preterm labour,
- Have experienced threatened preterm labour in this pregnancy,
- Are at high risk of preterm birth for another clinical reason, such as prior cervical surgery.

Principal inclusion criteria for women's postnatal focus groups

Women who have experienced preterm birth following preterm labour at <34 weeks whose babies **are stable and well** and are receiving care on the special care baby unit or neonatal intensive care unit.

Principal inclusion criteria for partners' focus groups

Partners of women who fit the eligibility criteria for either focus group.

Principal exclusion criteria for the focus groups

Non-English speaking individuals.

Principal inclusion criteria for clinician interviews

Clinicians who care for pregnant women i.e. obstetricians (including trainees), neonatologists and midwives.

Principal exclusion criteria for clinician interviews

For peer review only - http://bmjopen.bmj.com/site/about/guidelines.xhtml

Researchers in QUIDS or QUIDS qualitative.

Recruitment

Women and partners

Eligible women will be identified by clinicians in the preterm birth clinic and other antenatal clinics, and antenatal, triage or labour wards (for the antenatal focus groups) and the special care baby unit or postnatal clinics (for the postnatal focus groups) at each site. Eligible partners will be identified by the same method. Clinicians who are aware of and understand the research aims will approach women and partners to request consent for a researcher to contact them. Importantly, only postnatal parents whose babies are being cared for on the SCBU who are considered stable and well by the clinicians will be approached. With consent the researcher will make contact to talk to the women and/or their partners about the research, either face-to-face or over the telephone. Potential participants will be given the participant information sheet (PIS) (appendix _) that is relevant to them and given verbal information about the study. Each participant will be given time to read the information and the opportunity to have any questions answered. Willing participants will be asked to provide their written consent prior to the focus groups.

Clinicians

Eligible clinicians will be approached by the researchers, via email or face-to-face. Clinicians will be given the clinician PIS (appendix _) and the opportunity to read the information and have any questions answered. Willing clinicians will be asked to provide their written consent prior to the interviews.

For peer review only - http://bmjopen.bmj.com/site/about/guidelines.xhtml

All participants (women, partners and clinicians) will be reassured that they are not compelled to participate, that they can withdraw from the study at any time, and that non-participation will not affect their care or employment in any way.

Data collection

The primary aim of this research is to determine the decisional requirements of women, their partners and clinicians for the management of preterm labour. Qualitative semi-structured interviews, in a focus-group setting or individual telephone interviews, provide a means of collecting rich, in-depth data with a specific focus.[37] Hence, structured topic guides will be used to initiate and concentrate the discussion (appendices 7–10).

Focus groups are the preferred format for eliciting the view of women and women's partners. Encouraging discussion among a homogenous group with a shared interest is likely to provide rich insight and understanding into the group's experiences, beliefs and norms as a result of their social interaction.[38] Conversely, interviewing clinicians individually avoids the potential pitfall of professional embarrassment stifling ideas in a group setting. Interviewing individual clinicians with a range of professional experience should ensure that the decisional requirements of clinicians at all levels of experience are understood.

Demographic details and baseline characteristics will be collected prior to the interviews, either as a self-completion questionnaire, or questions asked by the researcher over the telephone. All interviews will be audio recorded, with the participants' consent, and field notes taken. The focus groups will be facilitated by at least two researchers. This is to ensure that all pre-specified areas of interest are covered and that non-verbal communication and group interactions are documented within the field-notes, which will provide context for the data analysis. Recapping will be used to

For peer review only - http://bmjopen.bmj.com/site/about/guidelines.xhtml

clarify aspects and avoid misinterpretation. To enable all participants to talk freely, the researchers will be unknown to the participants and not working clinically in the unit where the interview is conducted. Clinicians will be interviewed by a researcher who is unknown to them.

	Site	Interviewers
Women and partners' focus groups	Liverpool	HW and EO
	Birmingham	HW and VH-M
	Edinburgh	HW and LM
Clinician interviews	Telephone	HW (and EO?)

Analysis plan

A framework approach to data analysis will be used. This approach was developed to manage and interpret large volumes of data collected to inform health policy, meaning they had focussed aims and objectives.[37] Likewise, this research has clear aims, as described previously, in addition to the methodological aim of collecting rich data about the experiences and beliefs of women, their partners and clinicians in relation to managing preterm labour.

Framework analysis follows specific, clearly documented stages of analysis that are transparent so that others can review the interpretation processes and understand how the findings were reached.[39] Transparency is particularly important in this study as the findings will inform the development of an application to aid management decisions in clinical practice. Following verbatim transcription of the interview recordings, the researchers will become familiar with the data by reading the transcripts and field-notes several times. The next stage is to develop a theoretical framework by re-reading the transcripts and making notes as recurring characteristics are

For peer review only - http://bmjopen.bmj.com/site/about/guidelines.xhtml

recognised. The characteristics will then be collated into themes, which are based on the text itself, supported by the field-notes. The resulting thematic framework will be applied back to the transcripts and field-notes to check that it reflects the context of the original data. The transcripts will be coded, so that portions of text are linked to a discrete theme. A sample of transcripts will be independently coded by two people. The data will be charted and indexed to identify the preterm labour or professional experience of the participant, thus enabling the attribution of themes to a particular group. Finally, the content of the charts will be interpreted and mapped against each other to devise themes and sub-themes categories. Once again, this will involve review of the original data. Explanatory accounts will be developed to clarify the data and quotable sections of data will be identified. The final categories will be discussed between the researchers until consensus is met. The researchers will maintain reflexive journals throughout the data collection and analysis stages, recognising and ameliorating, as far as possible, the fact that their presence and assumptions impact on the data and the findings.[40]

This method of data analysis creates a clear audit trail thus ensuring rigour. Each stage of analysis refers back to the original data so that context and meaning is not lost in the final framework of themes and subthemes. The data analysis process will be managed using NVivo software, a qualitative data analysis tool.

Participant withdrawal

Participants may withdraw from the study at any point. However, they will not be able to withdraw use of their data once the prognostic tool is developed.

V 1.3
21/10/15

For peer review only - http://bmjopen.bmj.com/site/about/guidelines.xhtml

Safety

The physical safety of participants will be ensured through adhering to the health and safety policies of the host units where the focus groups take place.

The emotional wellbeing of the participants will be safeguarded by following the Distress Policy (see appendix 11). The Supervisors of Midwives (SOM) team in each unit will be informed of the study and women and their partners will be given the SOM team contact details, should they become distressed or upset as a result of talking about their experiences. Participants will also be given the contact details for accessing local counselling services.

Good clinical practice

Informed consent

All participants will be fully informed about the study and the subsequent QUIDS trial via verbal and written communication. All eligible individuals will be given the participant information sheet (appendix __) and provided with an opportunity to have any questions answered. Written consent will then be gained prior to the commencement of the focus groups/interviews.

Confidentiality

Demographic information will be collected from participants to attribute themes from the data to particular groups within the analysis and dissemination of findings. Demographic information, which will contain potentially identifiable information, will be kept in a secure lockable cabinet. Audio recordings will be stored on an encryptable audio device only until they are transcribed. Once transcribed the audio recordings will be deleted. Transcription services are provided by '1st Class Secretarial', who subscribe to the Data Protection Act and have also signed the Code of Practice on Data Handling. Hard copies of audio transcripts and field-notes will be kept in a separate secure

V 1.3
21/10/15

For peer review only

lockable cabinet to the demographic information. The transcripts and field-notes will be coded to identify which participant provided that data; the codes will only be known by the researchers. Participant's data will not be used for any purpose other than this study and the subsequent QUIDS trial.

Data Protection

Participants will be informed that publications from this study will contain direct quotes from the focus groups/interviews and categorisation of their experience of preterm labour (e.g. experienced preterm birth), which could enable personal identification.

All researchers involved in this study must comply with the requirements of the Data Protection Act 1998 with regard to the collection, storage, processing and disclosure of personal information and uphold the Act's core principles. All computers used for processing data are password protected and subject to the strict data protection policies of the researcher's institution.

Good clinical practice training

All researchers involved in this study must hold evidence of recent Good Clinical Practice training.

Additional ethical considerations

Expenses and reimbursement

Participants will be reimbursed for all out of pocket expenses, for example travelling to the interview site. Participants will be informed of this and how to apply for expenses reimbursement, including keeping receipts for travel.

For peer review only - http://bmjopen.bmj.com/site/about/guidelines.xhtml

For peer review only

Safety of researchers

An individualised risk assessment will be conducted to identify any risks to researchers or participants involved in this study. The lone working policy of the institution will be adhered to at all times. The only anticipated lone working will be during travel to and from the interview sites.

The lone working policy of the researcher's institutions mandates that researchers wear a GPS tracking and audio transmitting device during all lone-working, off-site research activity with participants. Participants will be informed if this device is being used.

Insurance / Indemnity

The researcher's institution holds public liability insurance and professional indemnity insurance (appendices 12, 13 and 14).

Timeline

The anticipated start date for the focus groups and interviews is 1st January 2016, to be completed within 3 months.

For peer review only - http://bmjopen.bmj.com/site/about/guidelines.xhtml

Appendices

Appendix 1: PIS women

Appendix 2: PIS partners

Appendix 3: PIS clinicians

Appendix 4: Consent form women

Appendix 5: consent form partners

Appendix 6: consent form clinicians

Appendix 7: Interview schedule AN women

Appendix 8: Interview schedule PN women

Appendix 9: Interview schedule partners

Appendix 10: Interview schedule clinicians

V 1.3
21/10/15

For peer review only

Appendix 11: Distress policy

Distress

- Participant indicates that they are experiencing high levels of stress, anxiety or emotional distress
- Participant exhibits signs suggestive of excessive stress anxiety or emotional distress e.g. shaking, uncontrolled crying

Response

- Stop interview / discussion
- Researcher (health professional) to offer immediate support
- Assess mental state - ASK
  - Tell me what thought you are having?
  - Tell me how you are feeling right now?
  - Do you feel able to go on with your day?
  - Do you feel safe?

Review

- If participant feels able to continue resume interview / discussion
- If not go to stage 2

Stage 2 Response

- Remove participant from discussion to a quiet area /stop interview
- Encourage participant to contact GP or other health provider, family member or friend OR
- Offer for a member of the research team to do so

Follow up

- Follow up participant with courtesy call (if participant consents) OR
- Encourage participant to call member of the research team if experiences increased distress in the days following an interview / focus group
- Refer to Supervisor of Midwives for further support and guidance if appropriate

Adapted from Haigh and Witham (2010)[41]

V 1.3
21/10/15

Appendix 12: Public Liability insurance

ZURICH®
MUNICIPAL

To Whom It May Concern

Our ref: SP/IND 3 June, 2015

Zurich Municipal Customer: The University of Manchester

This is to confirm that The University of Manchester have in force with this Company Public Liability Insurance until the policy expiry on 31 May 2016:

Policy Number: NHE-07CA03-0013

Limit of Indemnity: £ 50,000,000 any one claim

Excess: Nil any one claim

Yours faithfully

Underwriting Services
Zurich Municipal
Farnborough

Zurich Municipal
Zurich House
2 Gladiator Way
Farnborough
Hampshire
GU14 6GB

Telephone 0870 2418050
Direct Phone 01252 387859
Direct Fax 01252 375893
E-mail alison.cliff@uk.zurich.com

Communications will be monitored regularly to improve our service and for security and regulatory purposes

Zurich Municipal is a trading name of Zurich Insurance Group Ltd

A public limited company incorporated in Ireland. Registration No. 13460 Registered Office: Zurich House, Ballsbridge Park, Dublin 4, Ireland.

UK branch registered in England and Wales Registration No. BR7985. UK Branch Head Office: The Zurich Centre, 3000 Parkway, Whiteley, Fareham, Hampshire PO15 7JZ

Authorised by the Central Bank of Ireland and subject to limited regulation by the Financial Conduct Authority. Details about the extent of our regulation by the Financial Conduct Authority are available from us on request.

V 1.3
21/10/15

Appendix 13: Employers' Liability insurance

ZURICH MUNICIPAL

Certificate of Employers' Liability Insurance(a)

(Where required by regulation 5 of the Employers' Liability (Compulsory Insurance) Regulations 2008 (the Regulations), a copy of this certificate must be displayed at all places where you employ persons covered by the policy or an electronic copy of the certificate must be retained and be reasonably accessible to each employee to whom it relates).

Policy No.	NHE-07CA03-0013
1. Name of policyholder	The University of Manchester
2. Date of commencement of insurance policy	01 June 2015
3. Date of expiry of insurance policy	31 May 2016

We hereby certify that subject to paragraph 2:

1. The policy to which this certificate relates satisfies the requirements of the relevant law applicable in Great Britain, Northern Ireland, the Isle of Man, the Island of Jersey, the Island of Guernsey and the Island of Alderney **(b)**

2. **(a)** the minimum amount of cover provided by this policy is no less than £5 million **(c)**

Signed on behalf of Zurich Insurance plc (Authorised Insurer).

Signature

Stephen Lewis

Chief Executive Officer, Zurich Insurance plc (UK Branch)

Zurich Municipal is a trading name of Zurich Insurance plc A public limited company incorporated in Ireland Registration No.13460 Registered Office Zurich House, Ballsbridge Park ,Dublin 4 Ireland. UK branch registered in England and Wales Registration No. . BR 7985 UK Branch Head Office The Zurich Centre, 3000 Parkway, Whiteley, Fareham, Hampshire PO15 7JZ

Authorised by the Central Bank of Ireland and subject to limited regulation by the Financial Conduct Authority. Details about the extent of our regulation by the Financial Conduct Authority are available from us on request

Notes

(a) Where the employer is a company to which regulation 3(2) of the Regulations applies, the certificate shall state in a prominent place, either that the policy covers the holding company and all its subsidiaries, or that the policy covers the holding company and all its subsidiaries except any specifically excluded by name, or that the policy covers the holding company and only the named subsidiaries.

(b) Specify applicable law as provided for in regulation 4(6) of the Regulations.

(c) See regulation 3(1) of the Regulations and delete whichever of paragraphs 2(a) or 2(b) does not apply. Where 2(b) is applicable, specify the amount of cover provided by the relevant policy

V 1.3
21/10/15

Appendix 14: Professional indemnity insurance

MARSH

Cliffe Crowther
Marsh Ltd
Belvedere
12 Booth Street
Manchester
M2 4AW
+44 (0) 161 954 7317
Fax +44 (0) 161 954 7210
Cliffe.crowther@marsh.com
www.marsh.com

To whom it may concern

29th May 2015

Dear Sirs,

CONFIRMATION OF INSURANCE – The University of Manchester and Subsidiary Companies
As requested by the above client, we are writing to confirm that we act as Insurance Brokers to the client and that we have arranged insurance(s) on its behalf as detailed below:

PROFESSIONAL INDEMNITY INSURANCE

INSURERS	Novae Underwriting Ltd.
POLICY NUMBER	003210MMA15C
PERIOD OF INSURANCE	01 June 2015 to 31st May 2016, both dates inclusive.
LIMIT OF INDEMNITY	GBP10,000,000 any one claim and in the aggregate any one insurance period plus costs and expenses.
DEDUCTIBLE	GBP20,000 each & every claim including costs and expenses

Registered in England Number: 1507274, Registered Office:
1 Tower Place West, Tower Place, London EC3R 5BU.
Marsh Ltd is authorised and regulated by the Financial Conduct Authority

MARSH & McLENNAN COMPANIES

For peer review only - http://bmjopen.bmj.com/site/about/guidelines.xhtml

MARSH

Page 2
29th of May 2015

We have placed the insurance which is the subject of this letter after consultation with the client and based upon the client's instructions only. Terms of coverage, including limits and deductibles, are based upon information furnished to us by the client, which information we have not independently verified.

This letter is issued as a matter of information only and confers no right upon you other than those provided by the policy. This letter does not amend, extend or alter the coverage afforded by the policies described herein. Notwithstanding any requirement, term or condition of any contract or other document with respect to which this letter may be issued or pertain, the insurance afforded by the policy (policies) described herein is subject to all terms, conditions, limitations, exclusions and cancellation provisions and may also be subject to warranties. Limits shown may have been reduced by paid claims.

We express no view and assume no liability with respect to the solvency or future ability to pay of any of the insurance companies which have issued the insurance(s).

We assume no obligation to advise yourselves of any developments regarding the insurance(s) subsequent to the date hereof. This letter is given on the condition that you forever waive any liability against us based upon the placement of the insurance(s) and/or the statements made herein with the exception only of wilful default, recklessness or fraud.

This letter may not be reproduced by you or used for any other purpose without our prior written consent.

This letter shall be governed by and shall be construed in accordance with English law.

Yours faithfully

Cliffe Crowther

MARSH & McLENNAN COMPANIES

V 1.3
21/10/15

References

1. Beck S, Wojdyla D, Say L, *et al.* The worldwide incidence of preterm birth: a systematic review of maternal mortality and morbidity. *Bull World Health Organ* 2010;88:31–38. doi:10.2471/BLT.08.062554.
2. Health and Social Care Information Centre. *Hospital Episode Statistics: NHS Maternity statistics — England, 2013–14.* Leeds, HSIC.
3. Saigal S, Doyle LW. An overview of mortality and sequelae of preterm birth from infancy to adulthood. *Lancet* 2008;371:261–9.
4. D'Onofrio BM, Class QA, Rickert ME, *et al*. Preterm birth and mortality and morbidity: a population-based quasi-experimental study. *JAMA Psychiatry* 2013; 70(11):1231-1240. doi:10.1001/jamapsychiatry.2013.2107 .
5. Mangham LJ, Petrou S, Doyle LW, *et al.* The cost of preterm birth throughout childhood in England and Wales. *Pediatrics* 2009;123(2):e312–27. doi: 10.1542/peds.2008-1827.
6. Alfirevic Z, Allen-Coward H, Molina F, *et al.* Targeted therapy for threatened preterm labor based on sonographic measurement of the cervical length: a randomized controlled trial. *Ultrasound Obstet Gynecol* 2007;29:47–50. DOI: 10.1002/uog.3908.
7. Salim R, Garmi G, Nachum Z, *et al.* Nifedipine compared with atosiban for treating preterm labor: a randomized controlled trial. *Obstet Gynecol* 2012;120:1323–31. DOI:10.1097/AOG.0b013e3182755dff
8. Roberts D, Dalziel SR. Antenatal corticosteroids for accelerating fetal lung maturation for women at risk of preterm birth. *Cochrane Database of Systematic Reviews* 2006, Issue 3. Art. No.: CD004454. DOI: 10.1002/14651858.CD004454.pub2.
9. Crowther CA, McKinlay CJD, Middleton P, Harding JE. Repeat doses of prenatal corticosteroids for women at risk of preterm birth for improving neonatal health outcomes.

For peer review only - http://bmjopen.bmj.com/site/about/guidelines.xhtml

Cochrane Database of Systematic Reviews 2015, Issue 7. Art. No.: CD003935. DOI: 10.1002/14651858.CD003935.pub4.

10. Doyle LW, Crowther CA, Middleton P, Marret S, Rouse D. Magnesium sulphate for women at risk of preterm birth for neuroprotection of the fetus. *Cochrane Database of Systematic Reviews* 2009, Issue 1. Art. No.: CD004661. DOI:10.1002/14651858.CD004661.pub3.
11. Royal College of Obstetricians and Gynaecologists. *Tocolysis for women in preterm labour: Green-top Guideline No. 1b.* 2011. London: RCOG.
12. Murphy KE, Hannah ME, Willan AR, Hewson SA, Ohlsson A, Kelly EN, Matthews SG, Saigal S, Asztalos E, Ross S, Delisle M-F, Amankwah K, Guselle P, Gafni A, Lee SK, Armson BA, for the MACS Collaborative Group. Multiple courses of antenatal corticosteroids for preterm birth (MACS): a randomised controlled trial. *Lancet* 2008; 372: 2143–51.
13. Norberg H, Stålnacke J, Diaz Heijtz R, Smedler A-C, Nyman M, Forssberg H, Norman M. Antenatal corticosteroids for preterm birth: dose-dependent reduction in birthweight, length and head circumference. *Acta Paediatrica* 2011;100:364–9.
14. Asztalos E, Willan A, Murphy K, Matthews S, Ohlsson A, Saigal S, Armson A, Kelly E, Delisle M-F, Gafni A, Lee S, Sananes R, Rovet J, Guselle P, Amankwah K, for the MACS-5 Collaborative Group. Association between gestational age at birth, antenatal corticosteroids, and outcomes at 5 years: multiple courses of antenatal corticosteroids for preterm birth study at 5 years of age (MACS-5). *BMC Pregnancy and Childbirth* 2014; 14:272.
15. Jacquemyn Y, Zecic A, Van Laere D, Roelens K. The use of intravenous magnesium in non-preeclamptic pregnant women: fetal/neonatal neuroprotection. *Arch Gynecol Obstet* 2015;291:969–975.
16. Gyetvai K, Hannah ME, Ellen D, Hodnett ED, Ohlsson A. Tocolytics for preterm labour: a systematic review. *Obstetrics and Gynecology* 1999;94(5):869–77.
17. Hill WC. Risks and complications of tocolysis. *Clinical Obstetrics and Gynecology* 1995;38(4):725–45.

V 1.3
21/10/15

For peer review only

18. Kent RA, Yazbek M, Heyns T, et al. The support needs of high-risk antenatal patients in prolonged hospitalisation. *Midwifery* 2015;31:164–9.

19. Lasswell SM, Barfield WD, Rochat RW, Blackmon L. Perinatal Regionalization for Very Low-Birth-Weight and Very Preterm Infants: A Meta-analysis. *JAMA.* 2010;304(9):992-1000. doi:10.1001/jama.2010.1226.

20. Marlow N, Bennett C, Drasper ES, Hennessy EM, Morgan AS, Costeloe KL. Perinatal outcomes for extremely preterm babies in relation to place of birth in England: The EPICure 2 study. *Arch Dis Child Fetal Neonatal Ed* 2014;99:F181–88.

21. Towers TV, Bonebrake R, Padilla G, Rumney P. The effect of transport on the rate of severe intraventricular hemorrhage in very low birth weight infants. *Obstetrics and Gynecology* 2000;95(2):291–295.

22. Porcellato L, Masson G, O'Mahony F, Jenkinson S, Vanner T, Cheshire K, Perkins E. 'It's something you have to put up with'— service users' experiences of in utero transfer: a qualitative study. *BJOG* 2015; DOI: 10.1111/1471-0528.13235.

23. Bond PA, Crisp AS, Morgan MEI, Lobb MO, Cooke RWI. Maternal attitudes to transfer before delivery. *Journal of Reproductive and Infant Psychology* 1984;2(1):33–41. DOI: 10.1080/02646838408403447.

24. Wilson AM, MacLean D, Skeoch CH, Jackson L. An evaluation of the financial and emotional impact of *in utero* transfers upon families: a Scotland-wide audit. *Infant* 2010;6(2):38–40.

25. Coster-Schulz MA, Mackey MC. The preterm labour experience: a balancing act. *Clinical Nursing Research* 1998;335–59.

26. Gale C, Hay A, Phillip C, et al. In-utero transfer is too difficult: results from a prospective study. *Early Human Development* 2012;88:147–50.

27. Macintyre-Beon C, Skeoch C, Jackson J, Booth P, Cameron A. *Perinatal Collaborative Transport Study: Final report.* 2008. Glasgow: Scottish Neonatal Transport Service.

V 1.3
21/10/15

For peer review only

28. O'Brien ET, Quenby S, Lavender T. Women's view of high risk pregnancy under threat of preterm birth. *Sexual and Reproductive Health* 2010;1:79–84.

29. Hezelgrave NL, Shennan AH, David AL. Tests to predict imminent delivery in threatened preterm labour. *BMJ* 2015;350:h2183 doi: 10.1136/bmj.h2183.

30. Chandiramani M, Di Renzo GC, Gottschalk E, Helmer H, Henrich W, Hoesli I, Mol B, Norman JE, Robson S, Thornton S, Shennan A. Fetal fibronectin as a predictor of spontaneous preterm birth: a European perspective. *The Journal of Maternal-Fetal & Neonatal Medicine* 2011;24(2):330–336.

31. Deshpande SN, van Asselt ADI, Tomini F, Armstrong N, Allen A, Noake C, et al. Rapid fetal fibronectin testing to predict preterm birth in women with symptoms of premature labour: a systematic review and cost analysis. *Health Technol Assess* 2013;17(40).

32. Radford S, Abbott D, Seed P, Kemp J, Shennan. Quantitative fetal fibronectin for the prediction of preterm birth in symptomatic women. *Arch Dis Child Fetal Neonatal Ed* 2012;97(Supp 1):A1–A125.

33. Harrison MJ, Kushner KE, Benzies K, et al. Women's satisfaction with their involvement in health care decisions during a high-risk pregnancy. *Birth* 2003;30(2):109–15.

34. Hill JL, Campbell MK, Zou GY, et al. Prediction of preterm birth in symptomatic women using decision tree modelling for biomarkers. *Am J Obstet Gynecol* 2008;198:468.e1-468.e9.

35. Gallagher K, Martin J, Keller M, et al. European variation in decision-making and parental involvement during preterm birth. *Arch Dis Child Fetal Neonatal Ed* 2014;99:F245–9.

36. Peerzada JM, Schollin J, Håkansson S. Delivery room decision-making for extremely preterm infants in Sweden. *Pediatrics* 2006;117(6):1988–95.

37. Smith J, Firth J. Qualitative data analysis: the framework approach. *Nurse Researcher* 2011;18(2):52–62.

38. Rabiee F. Focus group interview and data analysis. *Proceedings of the Nutrition Society* 2004;63: 655–660.

V 1.3
21/10/15

For peer review only

39. Furber C. Framework analysis: a method for analysing qualitative data. *African Journal of Midwifery and Women's Health* 2010;4(2):97–100.

40. Sword W. Accounting for presence of self: reflections on doing qualitative research. *Qualitative Health Research* 1999;9(2):270–8.

41. Haigh C, Witham G. *Distress protocol for qualitative data collection.* 2015. [Online] available at: http://www2.mmu.ac.uk/media/mmuacuk/content/documents/rke/Advisory%20Distress%20Protocol.pdf accessed October 2015.

For peer review only

V 1.3
21/10/15

BMJ Open

Study Protocol: Quantitative Fibronectin to help Decision-making in women with Symptoms of Preterm Labour (QUIDS) Part Two- UK Prospective Cohort Study

Journal:	*BMJ Open*
Manuscript ID	bmjopen-2017-020795.R2
Article Type:	Protocol
Date Submitted by the Author:	27-Feb-2018
Complete List of Authors:	Stock, Sarah; University of Edinburgh MRC Centre for Reproductive Health, Tommy's Centre for Maternal and Fetal Health; University of Western Australia School of Women's and Infant's Health,<br>Wotherspoon, Lisa; University of Edinburgh MRC Centre for Reproductive Health, Tommy's Centre for Maternal and Fetal Health<br>Boyd, Kathleen; University of Glasgow, Health Economics & Health Technology Assessment<br>Morris, R. K.; Univ Birmingham, School of Clinical and Experimental Medicine<br>Dorling, Jon; Queen's Medical Centre, Neonatal Unit<br>Jackson, Lesley; Royal Hospital for Children Glasgow, Neonatal Unit<br>Chandiramani, Manju; Imperial College Healthcare NHS Trust, Queen Charlotte and Chelsea Hospital, Du Cane Road, Shepherds Bush, London, W12 0HS<br>David, Anna; University College London Medical School, Institute for Womens Health<br>Khalil, Asma; St. George's Medical School, University of London<br>Shennan, Andrew; Kings College London, Maternal and Fetal Research Unit<br>Hodgetts Morton, Victoria ; Birmingham Women's Hospital, Metchley Park Road, Edgbaston, ,<br>Lavender, Tina; Manchester University<br>Khan, Khalid; Queen Mary, University of London, Centre for Primary Care and Public Health<br>Harper-Clarke, Susan; PPI Representative<br>Mol, Ben; University of Adelade, The Robinson Institute, School of Paediatrics and Reproductive Health<br>Riley, Richard; Keele University<br>Norrie, John; Edinburgh Clinical Trials Unit, University of Edinburgh No. 9, Bioquarter<br>Norman, Jane; University of Edinburgh
**Primary Subject Heading**:	Obstetrics and gynaecology
Secondary Subject Heading:	Health economics, Diagnostics
Keywords:	Pregnancy, Preterm Birth, Fetal Fibronectin, Cervix, Diagnostic Test

For peer review only

Study Protocol: Quantitative Fibronectin to help Decision-making in women with Symptoms of Preterm Labour (QUIDS) Part Two- UK Prospective Cohort Study

Sarah J Stock*[1,2] Sarah.Stock@ed.ac.uk
Lisa Wotherspoon[1] Lisa.Wotherspoon@ed.ac.uk
Kathleen Boyd[3] Kathleen.Boyd@glasgow.ac.uk
Rachel K Morris[4] r.k.morris@bham.ac.uk
Jon Dorling[5] Jon.Dorling@nottingham.ac.uk
Lesley Jackson[6] Lesley.Jackson@ggc.scot.nhs.uk
Manju Chandiramani[7] m.chandiramani@imperial.ac.uk
Anna David[8] a.david@ucl.ac.uk
Asma Khalil[9] akhalil@sgul.ac.uk
Andrew Shennan[10] andrew.shennan@kcl.ac.uk
Victoria Hodgetts-Morton[4] v.a.h.morton@bham.ac.uk
Tina Lavender[11] Tina.Lavender@manchester.ac.uk
Khalid Khan[12] k.s.khan@qmul.ac.uk
Susan Harper-Clarke[13] su_clarke@hotmail.com
Ben Mol[14] ben.mol@adelaide.edu.au
Richard D. Riley[15] r.riley@keele.ac.uk
John Norrie[16] J.Norrie@ed.ac.uk
Jane Norman[1] Jane.Norman@ed.ac.uk

1. Tommy's Centre for Maternal and Fetal Health, University of Edinburgh MRC Centre for Reproductive Health, Queen's Medical Research Institute, Edinburgh, UK. 2. School of Women's and Infants' Health, University of Western Australia, Crawley WA 6009, AUS. 3. Institute of Health & Wellbeing Health Economics & Health Technology Assessment, University of Glasgow, 1 Lilybank Gardens, Glasgow, UK. 4. School of Clinical and Experimental Medicine, University of Birmingham, Birmingham, UK. 5. Queen's Medical Centre, Neonatal Unit, Derby Road,

For peer review only

Nottingham, UK. 6. Royal Hospital for Children Glasgow, Neonatal Unit Glasgow, UK. 7. Imperial College Healthcare NHS Trust, Queen Charlotte and Chelsea Hospital, Du Cane Road, Shepherds Bush, London, UK. 8. University College London Medical School, Institute for Women's Health Health, 86-96 Chenies Mews, University College London Medical School London, UK. 9. St. George's Medical School, University of London, London, UK. 10. Kings College London, Maternal and Fetal Research Unit Maternal and Fetal Research Unit 10th Floor, North Wing London, UK. 11. University of Manchester School of Nursing, Midwifery & Social Work, Jean McFarlane Building, University Place, Oxford Road Manchester, UK. 12. Queen Mary, University of London, Centre for Primary Care and Public Health London, UK. 13. Patient and Public Involvement Representative. No affiliation. 14. University of Adelaide, The Robinson Institute, School of Paediatrics and Reproductive Health Adelaide, SA, AUS. 15. Research Institute for Primary Care and Health Sciences, Keele University, Keele, UK. 16. Edinburgh Clinical Trials Unit, University of Edinburgh No. 9, Bioquarter Edinburgh, UK.

*Corresponding Author: Dr Sarah J Stock Senior Clinical Lecturer Maternal and Fetal Medicine, MRC Centre for Reproductive Health, Queen's Medical Research Institute, 47 Little France Crescent, Edinburgh, EH16 4TJ. Tel: 0131 242 6449 Email: sarah.stock@ed.ac.uk

Word Count: 5,475

Key Words: Pregnancy; Preterm Birth; Fetal Fibronectin; Cervix; Diagnostic Test.

For peer review only

ABSTRACT

Introduction: The aim of the QUIDS study is to develop a decision support tool for the management of women with symptoms and signs of preterm labour, based on a validated prognostic model using quantitative fetal Fibronectin (fFN) concentration, in combination with clinical risk factors.

Methods and analysis: The study will evaluate the Rapid fFN 10Q System (Hologic, Malborough, MA) which quantifies fFN in a vaginal swab. In QUIDS Part 2 we will perform a prospective cohort study in at least eight UK consultant-led maternity units, in women with symptoms of preterm labour at 22+0 to 34+6 weeks gestation to externally validate a prognostic model developed in QUIDS Part 1. The effects of quantitative fFN on anxiety will be assessed, and acceptability of the test and prognostic model will be evaluated in a subgroup of women and clinicians (n=30). The sample size is 1600 women (with estimated 96-192 events of preterm delivery within 7 days of testing). Clinicians will be informed of the qualitative fFN result (positive/negative) but be blinded to quantitative fFN result. Research midwives will collect outcome data from the maternal and neonatal clinical records. The final validated prognostic model will be presented as a mobile or web-based application.

Ethics and dissemination: The study is funded by the National Institute of Healthcare Research Health Technology Assessment (HTA 14/32/01). It has been approved by the West of Scotland Research Ethics Committee (16/WS/0068).

Registration details: The study has been registered with ISRCTN Registry (ISRCTN 41598423) and NIHR Portfolio (CPMS: 31277)

Version: Protocol Version 2, Date 1st November 2016

STRENGTHS AND LIMITATIONS OF THIS STUDY

Strengths

- Validation of a prognostic model in a separate prospective cohort study

For peer review only

• Health Economic Analysis to determine cost effectiveness from NHS perspective

Limitations

• Not a randomized control trial to test effectiveness of the model on improved patient outcomes

HOW PATIENTS ARE INVOLVED IN THIS STUDY

Patient representatives were consulted during the protocol development and have been invited to join the Project Management Group and the Trial Steering Committee. Prior to commencing QUIDS, we performed a qualitative study to determine the decisional needs of pregnant women with signs and symptoms of preterm labour, their partners and their caregivers. This is described in the separate protocol "QUIDS Qualitative" (Supplementary Material). The end product of QUIDS will be a decision support aid to help clinicians, women and their partners decide on management of threatened preterm labour, based on the results of the quantitative fFN. In QUIDS Qualitative women and clinicians indicated that they would prefer this to be on web based or mobile app based format, presenting the risk of preterm birth within seven days of testing.

For peer review only

INTRODUCTION

The overall aim of the QUIDS study is to develop a decision support tool for the management of women with symptoms and signs of preterm labour, based on a validated prognostic model using quantitative fFN testing. The study has been conceptually divided into two parts. In this, the protocol for QUIDS Part Two, we detail the protocol for a prospective cohort study. This will externally validate a prognostic model developed in QUIDS Part One.[1] More detailed background about the diagnosis of preterm labour and background to the study is provided in the introduction of QUIDS Protocol Part One.[1]

Fetal Fibronectin (fFN) is a biochemical test of preterm labour which has potential to help improve diagnosis of impending preterm delivery.[2] Much of the evidence about fFN to date relates to the qualitative fFN test, which provides a positive or negative result on the basis of a single threshold of 50ng/ml.[2,3] This test has been largely replaced with the Rapid fFN 10Q System, which provides a concentration of fFN (quantitative fFN), and as a continuous variable, may be a more useful predictor of preterm delivery. fFN is now only available with a quantitative analyser in the UK, but there is no consensus as to which women to use the test in, or how to interpret the results.

The QUIDS study will address this evidence gap by providing evidence about the potential value of the quantitative fFN test, along with guidance about how to interpret results. Here we detail the protocol for external validation of a prognostic model developed in QUIDS Part One.[1]

METHODS AND ANALYSIS

Aims and Methodologies

For peer review only - http://bmjopen.bmj.com/site/about/guidelines.xhtml

The aim of the QUIDS study is to develop a decision support tool for the management of women with symptoms and signs of preterm labour, based on a validated prognostic model using quantitative fFN testing.

The study protocol has been divided into two parts (see flow chart Figure 1). The protocols for Parts One and Two are reported in separate manuscripts.

In QUIDS Protocol Part One we have described how we will perform (i) an Individual Participant Data (IPD) meta-analysis, and (ii) and Economic Analysis. The protocol details how we will develop and internally validate a prognostic model using quantitative fFN (as a continuous variable) and other risk (prognostic) factors and to evaluate the added value of quantitative fFN toward this prognostic model performance. We will also provide an economic rationale for the prognostic model and analyze its cost-effectiveness from the perspective of the NHS.

In this, the QUIDS Protocol Part Two, we will detail the prospective cohort study to externally validate and, if necessary, refine the prognostic model. This will be performed in at least eight UK hospitals with different settings (rural/urban) and different levels of neonatal care facilities. In addition, acceptability of quantitative fFN testing, and effects on maternal anxiety will be performed. We will assess the potential cost-effectiveness of the final prognostic model/decision support tool. This additional analysis will allow us to model the full costs and effect impacts of the different prognostic model and compare these in a cost-effectiveness analysis to provide an evidence-based economic rationale for implementing the diagnostic tool in the NHS.

Endpoints

For peer review only

The primary endpoint of the prognostic model is spontaneous preterm delivery within seven days of qfFN test, in women less than 36 weeks' gestation. This was influenced by the preceding QUIDS Qualitative Study, which included focus group consultation to determine the decisional needs of women, their partners and clinicians (Supplementary Material). It is also a recognised clinically important endpoint, as antenatal steroids (which significantly reduce morbidity and mortality in preterm babies[4]) are most effective if delivery occurs within seven days of administration.

A secondary endpoint suggested by QUIDS Qualitative Study (Supplementary Material) consultation, was delivery within 48 hours of qfFN test. This analysis will be performed if feasible to do so within the constraints of the data available for model development and validation.[1]

Health technologies being assessed

The trial will evaluate the Rapid fFN 10Q System (Hologic, Malboroughm MA). This provides a concentration of fFN (ng/ml or INVALID) in a vaginal swab sample in 10 minutes. It is now the only commercially available fFN test system, and replaces the TLiQ rapid analyser system, which provided a qualitative fFN result (POSITIVE or NEGATIVE) based on a threshold of 50ng/ml. The Rapid fFN 10Q system is a point of care test, which clinical staff can easily perform. All reagents for fFN testing can be stored at room temperature and specimen collection kits, reagents, cassettes and the 10Q analyzer can be kept in clinical areas where women with symptoms of preterm labour are assessed so they can be conveniently accessed.

Vaginal swab samples are analysed by lateral flow; solid-phase immunochromatographic assay (the Rapid fFN Cassette), and interpreted in the 10Q Rapid analyser. 200 μL of the sample is pipetted into the sample application well of

For peer review only

the Rapid fFN Cassette using a polypropylene or polyethylene pipette. The sample will then flow from an absorbent pad across a nitrocellulose membrane via capillary action through a reaction zone containing murine monoclonal anti-fetal fibronectin antibody conjugated to blue microspheres (conjugate). The conjugate, embedded in the membrane, will be mobilized by the flow of the sample. The sample will then flow through a zone containing goat polyclonal antihuman fibronectin antibody that captures the fibronectin-conjugate complexes. The remaining sample will flow through a zone containing goat polyclonal anti-mouse IgG antibody that captures unbound conjugate, resulting in a control line. After 10 minutes of reaction time, the intensities of the test line and control line are interpreted with the 10Q Rapid analyser and a printed result provided as a concentration in ng/ml (0->500ng/ml) or INVALID. The result is invalid if the test does not meet internal quality controls that are performed automatically with every test. In the event of an invalid result, the test can be repeated with any remaining clinical specimen. A quality control can be performed by a reusable Rapid fFN 10Q QCette® QC Device, which verifies that the analyser performance is within specification.

Target population

The target population is pregnant women attending hospital with signs and symptoms of preterm labour.

Validation And Refinement Of Prognostic Model

Population

The prospective cohort study will include women with signs and symptoms of preterm labour at 22^{+0} to 34^{+6} weeks gestation in whom admission, transfer or treatment is being considered. These will be recruited from at least eight sites with a mix of rural/urban settings, and have different levels of neonatal care facilities, over 12 months.

For peer review only - http://bmjopen.bmj.com/site/about/guidelines.xhtml

Eligibility Criteria

The following inclusion criteria will apply at screening assessment (all apply):

- Women who are 22^{+0} to 34^{+6} weeks (or earlier gestation if the fetus is considered potentially viable).
- Women showing signs and symptoms of pre-term labour which may include any or all of back pain, abdominal cramping, abdominal pain, light vaginal bleeding, vaginal pressure, uterine tightenings or contractions.
- Women where hospital admission, interhospital transfer or treatment (antenatal steroids, tocolysis or magnesium sulphate) is being considered due to signs of pre-term labour.
- Women aged 16 years or above.

The broad inclusion criteria reflect current clinical practice and enable the generalisability of the results of the trial for routine clinical care. We will include women who re-attend seven days or more after initial recruitment with signs and symptoms of preterm labour and also women who remain symptomatic but undelivered seven days later in whom repeat testing by the clinician is deemed to be appropriate. This will be in line with manufacturer's recommendation for fFN testing.

The following inclusion criteria will apply on speculum examination:

- Cervical dilation ≤ 3cm
- Intact membranes
- No significant vaginal bleeding, as judged by the clinician.
- Once it has been established that the women meets the above criteria, on speculum examination, the fFN swab can be taken.

Participants that sign the consent but are not eligible upon examination to have an fFN swab taken will still be enrolled and have outcome data collected.

For peer review only

The following exclusion criteria will apply:

- Contraindication to vaginal examination (e.g. placenta praevia).
- Higher order multiple pregnancy (triplets or more).
- Moderate or severe vaginal bleeding.
- Cervical dilatation greater than 3cm.
- Confirmed rupture of membranes.
- Sexual intercourse, vaginal examination or transvaginal ultrasound in the preceding 24 hours factors may invalidate results. These women will be initially excluded from the study, but can be included if still symptomatic after 24 hours, when fFN accuracy will be restored.

For peer review only

Co-Enrolment

This trial involves validating a decision support tool relating to a test that is currently commonly used in clinical practice. As such, there are no additional interventions. Co-enrolment in other non-interventional trials will be allowed. Co-enrolment in trials of tocolytic treatments or other management strategies that may influence timing of delivery as a primary outcome will not be allowed. Participation in QUIDs would not preclude babies being subsequently involved in interventional trials. Co-enrolment will be recorded in the electronic case report form (eCRF).

Setting

The prospective cohort study will take place in at least eight consultant-led obstetric units in the UK. More than 93% of pregnant women in the UK deliver in consultant-led units.[5,6] The vast majority of women with symptoms of preterm labour will present to a consultant-led unit for assessment, either directly or following advice from their community midwife or General Practitioner.

For peer review only - http://bmjopen.bmj.com/site/about/guidelines.xhtml

The study will not include any community maternity units (staffed by midwives, with or without involvement of non-obstetric medical staff), which cover a small proportion of women, mainly in remote and rural areas. In the Perinatal Collaborative Transport Study (CoTS study) of perinatal transfers in Scotland,[7] which involved 52,727 births, only 69 (0.13%) women were transferred to a consultant-led obstetric unit from community maternity units, and only a proportion of these were for suspected preterm labour. The small number of women cared for in community maternity units means their inclusion would not be an efficient use of study resources.

Given that management of women with symptoms of preterm labour and inter-hospital transfer patterns are likely to vary depending on level of available neonatal care and distance to transfer, we will include a mixture of hospitals with different levels of neonatal care facilities in both rural and urban settings. We will include units with Special Care Units (providing special care for their own local population), Local Neonatal Units (providing special care and high dependency care and a restricted volume of intensive care) and Neonatal Intensive Care Units (larger intensive care units providing the whole range of medical, and sometimes surgical neonatal care for their local population and for babies and their families referred from the neonatal network in which they are based, and other networks when necessary). The hospitals will be chosen from different geographical settings (rural/urban) and from different regions of the UK.

If additional units wish to participate in the study we will consider including them, to increase recruitment rates. The UK Reproductive Health and Childbirth specialty group (clinical study group) have contributed to the study protocol and support the proposed trial.

For peer review only - http://bmjopen.bmj.com/site/about/guidelines.xhtml

Participant Selection And Enrolment

Women with signs and symptoms of preterm labour will be identified on presentation to obstetric services. A member of clinical staff, usually the doctor or midwife assessing the woman, will identify potentially eligible participants, provide a participant information leaflet and invite consent. A suitably trained member of clinical staff (doctor or midwife) or research team will consent participants.

Posters and leaflets will be situated in antenatal areas of participating hospitals to alert women that the study is taking place, and women will be allowed as much time as possible to consider participation without unduly delaying further clinical assessment. Participants will receive adequate oral and written information and appropriate participant information and informed consent forms will be provided.

Screening For Eligibility

The clinical likelihood of preterm delivery is usually evaluated by history and examination, which includes abdominal palpation, to assess strength and frequency of uterine contractions. If preterm labour is suspected, a vaginal speculum examination is performed where the cervix is inspected for dilatation, and evidence of vaginal bleeding and membrane rupture assessed. Swabs for fFN are usually taken at this point. Potential participants in the QUIDS study will be identified after the initial assessment and provided with information about the study. A combined 'Screening and Consent Form' will be used as a self-screening tool for potentially eligible participants. Informed consent will take place before speculum examination and the fFN swab has been taken. This approach means that samples are collected at routine speculum examination, as they would be if fFN is implemented in clinical practice, and participants avoid an additional vaginal examination.

Ineligible And Non-Recruited Participants

For peer review only

Certain exclusion criteria can only be assessed at speculum examination (for example vaginal bleeding or evidence of ruptured membranes), so a proportion of women will not be eligible for fFN testing after consent is given. These women will still be enrolled and delivery outcomes collected. The decision whether to use this data for analysis will be the decision of the Chief Investigator and Statisticians.

Withdrawal Of Study Participants

Women will be able to withdraw consent for us of their data at any time until the end of the study.

Study Assessments (See Table 1)

Eligibility Assessment (Screening And Recruitment)

Women presenting with signs and symptoms of pre-term labour will be identified on presentation to obstetric services. The doctor or midwife assessing the woman will identify potentially eligible participants and provide an invitation letter and short information leaflet.

After the woman has had the opportunity to consider whether she would like to participate, she will be asked to complete the Screening and Consent Form. The clinician will then decide whether the fFN test can be carried out. If the test can be carried out (according to manufacturer’s guidelines), then the participant will be fully enrolled and that their delivery outcomes will still be collected.

If the woman declines to participate and she is willing to provide a reason for this, the reason given will be entered on to an anonymous log. Baseline demographics will be collected on consenting women, together with height and weight, information on medical history, obstetric history, estimated date of delivery and presenting signs and symptoms.

The original consent form will be stored in the Investigator Site File (ISF) file, a copy is given to the woman, a copy added to the medical notes and a copy sent to the Trial Office.

After providing consent, the participant will be asked to complete a short State Trait Anxiety Inventory (STAI) questionnaire and complete a contact details form. They will also be issued with a letter thanking them for taking part in the trial and giving details of the second questionnaire to be completed.

Sample Collection

Samples for analysis will be taken with a fFN specimen collection kit, which consists of a sterile polyester tipped swab and a specimen transport tube containing 1 ml extraction buffer (an aqueous solution containing protease inhibitors and protein preservatives including aprotinin, bovine serum albumin, and sodium azide). During speculum examination the sterile swab will be lightly rotated across the posterior fornix of the vagina for ten seconds to absorb vaginal secretions. Samples should be taken before any other swabs (e.g. for microbiology) or cervical manipulation and the speculum lubricated with normal saline as other lubricants may interfere with the antibody-antigen reaction of the test. Following specimen collection the swab should be removed, immersed in extraction buffer, the shaft of the swab snapped off, and the transport tube sealed.

Before analysis samples are gently mixed and as much liquid as possible expressed from the swab by rolling the tip against the inside of the tube.

Initial fFN test

For peer review only

The sample taken will be run at a near bedside Hologic Rapid fFN 10Q analyser, specially adapted for the QUIDS study. As fFN (or other similar biochemical tests of preterm labour) are part of standard care, it would be unethical to blind clinicians from the qualitative fFN result. The analyser will thus reveal a qualitative fFN result (positive/negative/invalid based on a 50ng/ml threshold) for clinicians to base clinical decision-making on, according to local protocols. The quantitative fFN result however, will be stored as a three-letter code, blinding caregivers from the result. Samples will be run as per manufacturers instructions (described above in the section "Health technologies being assessed").

Repeat fFN Tests

If there is clinical indication for further fFN tests (eg because of ongoing symptoms of preterm labour after seven days), the results will also be recorded.

Labour/Delivery/ Neonatal Assessments

Admission for delivery will not be a formal study visit but data will be collected using information recorded in the participant's notes. Delivery data will be collected on the maternal outcomes of delivery, including method of delivery, indication for delivery method, onset of labour, date and gestation of delivery and blood loss.

Questionnaires

All participants who are eligible to participate will be asked to complete a STAI questionnaire before the speculum examination. The same questionnaire will be repeated 24-48 hours post examination. The second questionnaire will be provided on paper with a pre-paid envelope to be returned by post to the Trial Office. If not returned by post, the Trial Office may try to contact the participant (with the contact details provided), to complete the questionnaire over the phone.

For peer review only - http://bmjopen.bmj.com/site/about/guidelines.xhtml

	Attendance with signs and symptoms preterm labour			
**Visit**	**Screening and Recruitment**	**24-48h**	**1-6 months**	**DELIVERY**
Inclusion/Exclusion Criteria	⊙			
Participant Information Sheet	⊙			
Consent Form	⊙			
Demographics	⊙			
Obstetric History	⊙			
Symptoms and Signs	⊙			
Quantitative fFN (concentration ng/ml)	⊙			
Cervical length scan (if available)	⊙			
State Trait Anxiety Inventory Questionnaire	⊙	⊙		
Delivery details				⊙
Neonatal outcomes				⊙
Qualitative Acceptability Questionnaires (subgroup n=30)			⊙	

Table 1: QUIDS Study Assessments

For peer review only

For peer review only - http://bmjopen.bmj.com/site/about/guidelines.xhtml

Safety and Quality Assessments

The Hologic Rapid fFN 10Q analyzer has integrated quality control measures, and we will keep records of these as well as any additional staff training that occurs after the study starts. It is recommended that a daily pre-calibrated reusable quality control cassette be inserted and analysed every 24 hours to verify that the analyser performance is within specification. A daily quality control (QC) should be performed if one has not been done in the preceding 24 hours before a patient test is to be done. Logs of results are stored on the machine and can be downloaded, and we will also ask the participating sites to keep a monthly paper log of QC tests done. Each patient test has an internal quality control, with a procedural control line that verifies the threshold level of signal by the instrument. Sample flow detection ensures the sample travels across the cassette properly, and confirms absence of conjugate aggregation. We believe that these measures will help ensure the validity of results. However, to provide further evidence of integrity and comparability of results from each site we will request that all participating sites enrol in the Wales External Quality Assurance Scheme (WEQAS) Point of Care Quality Assurance Scheme. WEQAS will provide a sample for analysis to each site bimonthly, and provide reports on analyser performance and variability.[8]

Data Collection

Data For Prognostic Model Validation and Update of Health Economic Model

We will collect data on all of the candidate predictors considered for inclusion in the prognostic model developed in the IPD meta-analysis (quantitative fFN concentration, previous spontaneous preterm labour, gestation at fFN test, age, ethnicity, BMI, smoking, deprivation index, number of uterine contractions in set time period, cervical dilatation, vaginal bleeding, previous cervical treatment for cervical intraepithelial neoplasia, cervical length [measured by transvaginal cervical length; when available], singleton/multiple pregnancy, tocolysis and fetal sex). Outcome data will include gestational age at delivery, date and time of delivery, administration of

For peer review only - http://bmjopen.bmj.com/site/about/guidelines.xhtml

treatments for preterm labour (steroids, antibiotics, tocolysis, magnesium sulphate) duration hospital admission, hospital transfer, onset of labour (preterm prelabour rupture of membranes; idiopathic preterm birth; medically indicated preterm birth [and indication]), place of delivery (base hospital, other hospital, outwith hospital), mode of delivery, neonatal admission, neonatal complications, perinatal mortality, congenital anomaly, sex and birthweight.

Screening data and data about quantitative fFN testing will be collected on paper based CRFs and research midwives will input these into the web based electronic database. Clinical outcome data will be collected from the medical records.

Maternal Acceptability and Anxiety

Maternal anxiety will be measured pre and post-test (24-48h) using the validated State Trait Anxiety Inventory (STAI) questionnaire. Acceptability of fFN testing and the decision support will be assessed using follow up interviews (face to face or telephone, according to maternal preference) which will be conducted with a sub-group of participants (n=30) purposively sampled and stratified according to geographical location, outcome (preterm labour or not) and anxiety scores. Acceptability will also be assessed in a cohort of clinicians (n=30).

Statistics and Sample Size Calculation

Guidance for external validation suggests at least ten events (preterm delivery within seven days of test) are required for each covariate included in a prognostic model.[9,10] Data from the cohorts included in our IPD meta-analysis suggests an event rate of between 6 and 12%.[1] Based on these estimates a sample size of 1,600 will provide 96 and 192 events (preterm delivery within 7 days).

A UK study has shown that 8.9% of pregnant women present with symptoms of preterm labour and are eligible for quantitative fFN[11] and we anticipate 50%

For peer review only

recruitment rate is achievable, thus overall 4.5% of maternities could be recruited. We will initially include eight units in the cohort study with a combined delivery rate of approximately 36,000 per annum. We anticipate that we will achieve target recruitment within 12 months (1 year * 36,000 * 0.089 * 0.5 = 1,602). If however, the recruitment rate or event rate is lower than predicted, we will increase the number of sites included in the study and/or the recruitment period, to ensure that a minimum of 60 events (preterm delivery within 7 days of test) are achieved, allowing for external validation of at least six covariates in our model.

It is possible that the IPD meta-analysis will find there is potential added value of combining quantitative fFN testing with cervical length measurement.[12,13] As cervical length measurement has significant resource requirement (estimated NHS cost £68.16 per test) and lack of out of hours provision further limits availability in many NHS hospitals, we think it is very unlikely that cervical length scanning will improve performance of the prognostic model to such a degree as to make it cost effective. We will assess the incremental costs and effects of cervical length measurement in the proposed health economic model performed in parallel with the IPD meta-analysis, and will feed into design considerations during the first iteration of the prognostic model.

If inclusion of cervical length ultrasound is found to be potentially cost-effective, we will assess the feasibility of including it in the prospective cohort study. We anticipate that including cervical length measurement in the prospective cohort study would be extremely difficult in the current NHS setting as the majority of units do not have 24 hour availability of transvaginal ultrasound and/or trained personnel to perform scans. Inclusion of cervical length would also likely decrease recruitment rate (due to need for additional transvaginal ultrasound examination) and require significant additional resources.

For peer review only

Analysis

Validation Of Prognostic Model

The prognostic model developed in the IPD will be externally validated using data collected in the prospective cohort data, using the measures of discrimination and calibration described in QUIDS Protocol Part One,[1] including R^2, C statistic, calibration slope, calibration-in-the-large, and calibration plots of observed versus predicted risks across deciles (with Loess smoother). The average performance of the model will be summarised across the centers in the cohort study. Between-center heterogeneity in performance will also be summarised, and reduced (if necessary) by recalibration techniques regarding the strategy for the choice of baseline risk (intercept). That is, the predictor effects will not be modified from the IPD meta-analysis model, but the intercept may need to be tailored to improve validation in UK centers (e.g. for rural settings). Based on the findings, a final model and its implementation strategy will then be recommended for use.

Economic Analysis

The economic model will be refined, integrated and updated with data from the prospective study cohort, so as the most up to date and validated evidence is used to inform a cost-effectiveness decision. Such an iterative approach to economic evaluation is now well established.[14,15] The care pathway following diagnosis will be included in the economic analysis, using data from the cohort study such as the diagnostic test accuracy data, resource use data (i.e. steroid use, other medications, time in hospital, hospital transfer) and secondary outcome data (i.e., treatment of side-effects, morbidity, mortality) so as to capture the full costs and effect impacts (quality of life, morbidity and mortality) for both the mother and baby. Resource use data will be combined with unit cost information from the British National Formulary[16] and NHS reference costs.[17,18] Outcomes will be reported as the incremental cost per correct diagnosis, and incremental cost per Quality Adjusted Life Year (QALY) gained of the qfFN prognostic model compared to current practice

For peer review only

(no qualitative fFN model). The analysis will adhere to the NICE reference case and the recommended guidelines for decision modeling and reporting of economic analyses.[18] Probabilistic sensitivity analysis will be undertaken to explore how uncertainty in the model inputs impact on the cost-effectiveness outcome.[19]

Acceptability of fFN Testing and Effects on Anxiety

Maternal anxiety will be measured before and after quantitative fFN testing using the validated STAI. The STAI Form Y is a widely used tool for measuring both temporary "state anxiety" and the more general, long-standing "trait anxiety". The STAI is designed for the self-reported assessment of the intensity of feelings of apprehension, tension, nervousness, and worry. STAI-Anxiety scores increase in response to physical danger and psychological stress, making it highly appropriate for this study. The use of STAI in pregnancy studies is discussed by Hundley, et al and we will interpret the results accordingly.[20]

The questionnaire will be administered prior to fFN testing (baseline) and 24-48 hours after the test, to assess early reactions to the test and any acute anxiety prompted by the result of the test. We will also be able to assess any differences in those presented with a high risk or low risk result. Although it might be interesting to assess anxiety again in the latter stages of pregnancy, it is likely that, in this population, many pregnancies will not reach full term. Thus we believe our strategy of repeat questionnaire administration will allow measurement of longer term anxiety induced or alleviated by the test, whilst minimising bias due to preterm or term delivery itself or loss to follow up.

Follow up interviews will be performed with a sub-group of participants (n=30) to enable deeper exploration of women's views regarding fFN testing, to gain insight into the rationale for responses given in the questionnaires. Interviews will be conducted following confirmation of pregnancy status. Acceptability of the prognostic

For peer review only - http://bmjopen.bmj.com/site/about/guidelines.xhtml

model will also be assessed with women and a group of clinicians. All interviews will be audio recorded with consent, and field notes taken to ensure an audit trail.

Decision Support

We will develop a decision support tool in accordance with the guidelines produced by the International Patient Decision Aid Standards (IPDAS) Collaboration.[21] Scoping of decisional requirements and how data should be presented was performed during focus group consultation as part of QUIDS Qualitative (Supplementary Material). A prototype decision support tool incorporating the initial prognostic model developed as part of the IPD-meta-analysis, will be tested with women and clinicians, as part of the acceptability studies described above. A final version will be updated with the validated (and, if necessary revised) prognostic model generated from the prospective cohort study. The multidisciplinary trial steering committee will oversee the development process, and decide how material is selected for inclusion.

Trial Management And Oversight Arrangements

Project Management Group

The trial will be coordinated by a Project Management Group (PMG), consisting of the grant holders (Chief Investigator and Co-applicants), the trial manager, representatives from the Study Office and CHaRT (the supporting CTU), plus service user representatives (PAG). The PMG will meet approximately every four months by teleconference or face to face.

The Trial Manager based in Edinburgh will oversee the study and will be accountable to the Chief Investigator. The Trial Manager supported by the trial administrator(s) will take responsibility for the day-to-day transaction of study activities. They will be supported by the CTU at CHaRT to provide expertise and guidance. The Trial Manager will be responsible for checking the CRFs for completeness, plausibility and

For peer review only - http://bmjopen.bmj.com/site/about/guidelines.xhtml

consistency. Any queries will be resolved by the Investigator or delegated member of the trial team.

A Delegation Log will be prepared for each site, detailing the responsibilities of each member of staff working on the trial.

Trial Steering Committee and Data Monitoring Committee

A combined Trial Steering Committee and Data Monitoring Committee (TSC/DMC) will oversee the conduct and progress of the trial. The terms of reference of the Committee will be developed separately. Members of the TSC/DMC will consist of experts and two patient representatives.

PATIENT AND PUBLIC INVOLVEMENT

Patient representatives were consulted during the protocol development and have been invited to join the Project Management Group and the Trial Steering Committee, and will thus be involved in the recruitment to, and conduct of, the study. Co-author Susan Harper-Clarke is a patient representative. Prior to commencing QUIDS, we performed a qualitative study to determine the decisional needs of pregnant women with signs and symptoms of preterm labour, their partners and their caregivers. This is described in the separate protocol “QUIDS Qualitative” (Supplementary Material). The end product of QUIDS will be a decision support aid to help clinicians, women and their partners decide on management of threatened preterm labour, based on the results of the quantitative fFN. In QUIDS Qualitative women and clinicians indicated that they would prefer this to be on web based or mobile app based format, presenting the risk of preterm birth within seven days of testing. Social media will be used to signpost publications and conference presentations and highlight important findings. Twitter and Facebook will be used to disseminate findings to professional organizations, charities, stakeholders and the

For peer review only - http://bmjopen.bmj.com/site/about/guidelines.xhtml

public. Communication to the general public will further be facilitated by our close links with charities such as Tommy's.[22]

ETHICS AND DISSEMINATION

The study will be conducted in accordance with the principles of Good Clinical Practice (GCP). A favorable ethical opinion has been obtained from the appropriate REC (reference 16/WS/0068) and local R&D approval will be obtained prior to commencement of the study at each site.

On completion of the study, the study data will be analysed and tabulated, and a clinical study report will be prepared in accordance with GCP guidelines. Results will be communicated to the academic community via the scientific literature, attendance at conferences and invited presentations. Summaries of results will also be made available to investigators for dissemination within clinics. We anticipate that the decision support will be made available as web based application that will be made freely available so clinicians can access it easily and it can be readily translatable into UK practice. If it is found to be effective in ruling out preterm delivery, it is likely that it will decrease unnecessary costly, and potentially harmful treatments in women who have symptoms suggestive of preterm labour but do not deliver early.

PEER REVIEW

The study was extensively peer reviewed as part of the process of gaining grant funding from the NIHR HTA (14/32/01).

For peer review only

FUNDING

This project was funded by the National Institute of Healthcare Research Health Technology and Assessment (Reference 14/32/01). The views expressed are those of the authors and not necessarily those of the NHS, the NIHR or the Department of Health.

CONTRIBUTIONS TO AUTHORSHIP

SJS, KB, RKM, JD, LJ, MC, AD, AK, AS, VHM, TL, KK, SHC, BM, RDR, JN and JEN developed the protocol. SJS, LW, RDR, KB, TL and JN drafted the protocol. RKM, JD, LJ, MC, AD, AK, AS, VHM, KK, SHC, BM and JEN reviewed and commented on the protocol.

COMPETING INTERESTS

SJS and JEN work at the University of Edinburgh, who received £1000 sponsorship from Hologic to support a meeting (The Society of Reproductive Investigation and MRC Centre for Reproductive Health Scientific Symposium on Targeting Inflammation to Improve Reproductive Health across the Lifecourse – August 2017).

AS has in the past (over last five years; not in the last three years) received funding for expenses related to advisory board and internal staff education from Hologic.

MC received sponsorship from Hologic to organise an educational teaching focusing on prediction of Preterm Birth at the 2017 annual meeting of the British Maternal and Fetal Medicine Society.

Hologic, the makers of fFN have provided analysers and technical support for their use to sites participating in the QUIDS prospective cohort study. They have no access to the data, or other involvement in the conduct, data analysis, interpretation of results or decision to publish the results of the study.

For peer review only

REFERENCES

1. Stock SJ, Wotherspoon L, Boyd K et al. Study Protocol: Quantitative Fibronectin to help Decision-making in women with Symptoms of Preterm Labour (QUIDS) Part One- Individual Patient Data Meta-analysis and Health Economic Analysis. *Submitted to BMJ Open alongside this manuscript(see supplementary information)*

2. Honest H, Forbes CA, Duree KH, et al. Screening to prevent spontaneous preterm birth: systematic reviews of accuracy and effectiveness literature with economic modelling. Health Technol Assess 2009;13(43):1-627

3. Deshpande SN, van Asselt AD, Tomini F, et al. Rapid fetal fibronectin testing to predict preterm birth in women with symptoms of premature labour: a systematic review and cost analysis. Health Technol Assess 2013;17(40):1-138

4. Roberts D, Brown J, Medley N, Dalziel SR. Antenatal corticosteroids for accelerating fetal lung maturation for women at risk of preterm birth. Cochrane Database Syst Rev 2017;3:CD004454

5. Office for National Statistics (2013). ONS gestation-Specific Infant Mortality in England and Wales 2011. Cardiff ONS, 2013. Also available at: https://www.ons.gov.uk/peoplepopulationandcommunity/healthandsocialcare/causesofdeath/bulletins/pregnancyandethnicfactorsinfluencingbirthsandinfantmortality/2013-10-10 (accessed 23rd November 2017).

6. Information Services Division (2013). "Births in Scottish hospitals 2012". Edinburgh National Services Scotland, 2013. Also available at: http://www.isdscotland.org/Health-Topics/Maternity-and-Births/Publications/2013-08-27/2013-08-27-Births-Report.pdf?34511965514 (accessed 23rd November 2017).

7. Macintyre-Beon C, Skeoch C, Jackson L, Booth P, Cameron A. Scottish Perinatal Collaborative Transport Study. NHS Quality Improvment Scotland 2008

8. http://www.weqas.com/services/poct-eqa/ (accessed 23rd November 2017).

9. Vergouwe Y, Steyerberg EW, Eijkemans MJ, Habbema JD. Substantial effective sample sizes were required for external validation studies of predictive logistic regression models. J Clin Epidemiol 2005;58(5):475-83

10. Vittinghoff E, McCulloch CE. Relaxing the rule of ten events per variable in logistic and Cox regression. Am J Epidemiol 2007;165(6):710-8

11. Abbott DH, Seed PT, Bennett PR et al. EQUIPP: Evaluation of Fetal Fibronectin with a novel bedside Quantitative Instrument for the Prediction of Preterm birth. Arch Dis Child Fetal Neonatal Ed 2014;99:A150-A51

12. van Baaren GJ, Vis JY, Wilms FF, et al. Predictive value of cervical length measurement and fibronectin testing in threatened preterm labor. Obstet Gynecol 2014;123(6):1185-92

13. DeFranco EA, Lewis DF, Odibo AO. Improving the screening accuracy for preterm labor: is the combination of fetal fibronectin and cervical length in symptomatic patients a useful predictor of preterm birth? A systematic review. Am J Obstet Gynecol 2013;208(3):233 e1-6

14. Ades AE, Sculpher M, Sutton A, et al. Bayesian methods for evidence synthesis in cost-effectiveness analysis. Pharmacoeconomics 2006;24(1):1-19

15. Sculpher MJ, Claxton K, Drummond M, McCabe C. Whither trial-based economic evaluation for health care decision making? Health Econom 2006;15(7):677-87

16. British National Formulary. BNF 73. 2017. .

17. Department of Health. Information Services Division. Scottish Health Service Costs Book 2012. 2012. Information Services Division.

18. National Institute for Health and Care Excellence. Guide to the Methods of Technology Appraisal: 2013. 2013 National Institute for Health and Clinical Excellence.

19. Briggs AH. Handling uncertainty in cost-effectiveness models. Pharmacoeconomics 2000;17(5):479-500

For peer review only

20. Hundley V, Gurney E, Graham W, Rennie AM. Can anxiety in pregnant women be measured using the State-Trait Anxiety Inventory. Midwifery 1998;14(2):118-21

21. http://ipdas.ohri.ca (accessed 23rd November 2017)

22. https://www.tommys.org (accessed 23rd November 2017)

Figure Legends

Figure 1

Flow chart illustrating the design of QUIDS study and conceptual division into Part 1 and Part 2

For peer review only

Figure 1

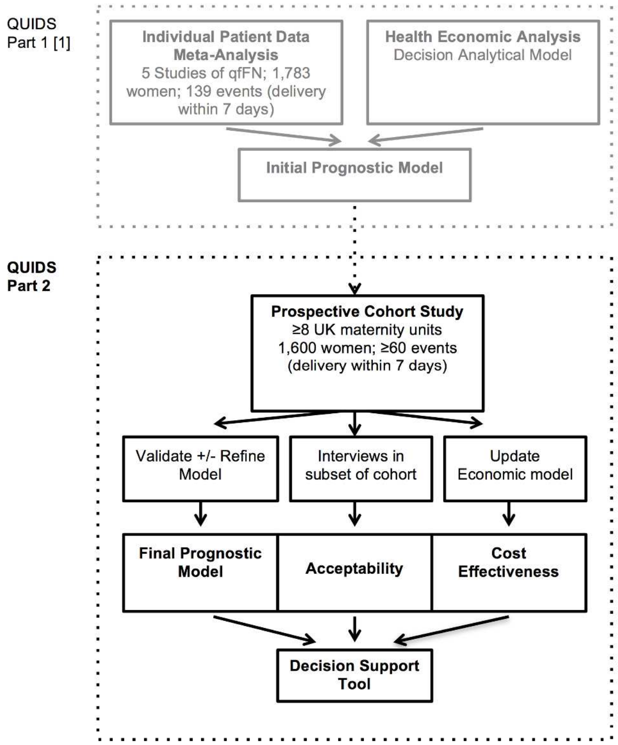

209x297mm (300 x 300 DPI)

QUIDS Qualitative

Quantitative Fibronectin to help Decision-making in women with Symptoms of Preterm Labour:

determining decisional requirements

Protocol

(October 2015)

White H, O'Brien E, Hodgetts-Moreton V, Stock S, Lavender T

For peer review only

Background

Preterm birth, defined as birth prior to 37 weeks gestation, occurs in 6-7% of pregnancies in Europe[1] and was recorded as 5.78% in England in 2013/14, equating to over 37,000 births.[2] Preterm birth is associated with a high risk of mortality, wide-ranging short- and long-term morbidities,[3,4] and significant economic costs to the NHS compared with birth at term.[5] Reducing the detrimental impact of preterm birth relies on the provision of timely and appropriate perinatal interventions. However, accurate prediction of preterm birth is challenging, even when the clinical symptoms are suggestive of preterm labour. In randomised trials approximately 80% of women diagnosed with preterm labour remained pregnant after 7 days.[6,7]

Interventions in preterm labour and preparations for preterm birth may include administration of corticosteroids to accelerate fetal lung maturation[8,9] and magnesium sulphate for fetal neuroprotection,[10] in utero transfer to a facility with appropriate maternity and neonatal services, and tocolysis to optimise time before birth to enable these.[11] Whilst such interventions can improve outcomes for mothers and babies who do experience preterm birth, they are not necessarily benign, especially for those in whom preterm birth does not occur.

The maximal beneficial impact of corticosteroids occurs with administration between 48 hours and seven days before birth, thus timing is especially important in optimising benefit for the neonate. For women who remain at risk of preterm birth after seven days of the initial dose, repeated doses reduce respiratory distress in the neonate[9] but have been found to be associated with a dose-dependent reduction in birthweight.[12,13] A five-year follow-up study of women who received repeated doses of antenatal corticosteroids due to risk of preterm birth found an increased risk of neurodevelopment impairment in infants born at term.[14] Therefore developing a strategy to establish the optimal time to give steroids is a research priority.

For peer review only - http://bmjopen.bmj.com/site/about/guidelines.xhtml

Magnesium sulphate administration immediately prior to birth has been shown to reduce cerebral palsy,[10] but there is a risk of magnesium toxicity leading to respiratory depression in the mother and, theoretically, the neonate.[15]

Whilst there is no clear beneficial effect of tocolytics on the incidence or outcome of preterm birth,[16] their use is recommended if the days gained prior to preterm birth can be used appropriately, for example transfer to a suitable maternity unit or the administration of drugs to protect the neonate.[11] Tocolysis is linked with various maternal and neonatal complications,[17] hence the need for therapy targeted only for those at risk of preterm birth and close monitoring of the mother and fetus throughout.

Often, inpatient admission is recommended if preterm labour is suspected. Previous literature has highlighted the social isolation and support needs that women with high-risk pregnancies who are hospitalised experience.[18] In some cases, in-utero transfer is indicated to ensure that birth takes place in a specialist unit with appropriate neonatal care facilities. This policy has been shown to reduce mortality[19,20] and morbidity[21] in preterm neonates, especially those born very premature. Qualitative research has indicated that women generally acknowledge the potential benefit of in utero transfer to their baby and, hence, are willing to endure the inconvenience and upheaval that it entails.[22,23] However, the experience is associated with an emotional, social and financial burden on women and their families, especially for the substantial proportion of women who do not deliver prematurely following in utero transfer. When describing their experiences of in utero transfer, women expressed shock at the prospect of the transfer, feeling socially isolated, and having no control over the situation, in addition to the practical difficulties experienced particularly by women who already had children.[22,24,25] In a large survey of women who had experienced in utero transfer, over a quarter lamented the financial cost[24] particularly with respect to their partner's outlay for travel, food, accommodation, and phone bills, exacerbated with requiring time off work.[22] Furthermore, in utero transfer is costly to maternity services. Securing a maternal and neonatal bed

For peer review only - http://bmjopen.bmj.com/site/about/guidelines.xhtml

in another unit is a time-consuming task that often falls to delivery suite midwives to arrange, whilst also continuing to provide care to the woman.[26] In a large observational study of all in utero transfers that took place in Scotland in a six-month period, nearly one third of all transfers were due to threatened preterm labour.[27] Under half of the women transferred from one consultant-led unit to another gave birth within 48 hours.[27] Such unnecessary transfers are costly to women, their families and maternity services. Qualitative research into women's experiences of preterm labour have highlighted the need for caregivers to create an environment where women are enabled to discuss their fears[28] and exert control over how they manage their preterm labour care.[25]

Accurate prediction of preterm birth could reduce the burdens and risks associated with unnecessary interventions, and enable women and their clinicians to make informed decisions regarding their care. Numerous diagnostic tests have been used in preterm labour, including biochemical tests of vaginal secretions and cervical length.[29] One such test is fetal fibronectin, a near-bedside test that provides a positive or negative result and has excellent negative predictive value.[30] Thus fetal fibronectin can identify which women will not benefit and may be put at risk by the interventions described previously, and reduce costs to maternity services.[31] Developments in fetal fibronectin testing have led to a quantitative test that provides a concentration of fetal fibronectin in vaginal secretions, giving women and clinicians more information on which to base their management decisions.[32]

Qualitative evidence has indicated that women feel a sense of increased responsibility to their babies and themselves during a high risk pregnancy, such as threatened preterm labour.[33] Women want to be involved in decision making about their care to different degrees and feel most satisfied when their caregiver supports them to make decisions in the way they felt most comfortable.[33]

For peer review only

For peer review only - http://bmjopen.bmj.com/site/about/guidelines.xhtml

Previous literature on decision making and preterm birth has focussed on diagnostic tests[6,28–32,34] and the care of the preterm infant.[35,36] To date, there has been no investigation of what women, their partners and caregivers would like to know in order to make informed decisions about the care that is provided following the signs and symptoms of preterm labour.

Funding has been received from the National Institute for Health Research Health Technology Assessment Programme for a large, multicentre trial to develop a mobile application decision support tool for the management of women with symptoms and signs of preterm labour, based on a validated model using quantitative fetal fibronectin testing. This study is the precursor to that trial, with the aim of determining the decisional needs of pregnant women with the symptoms and signs of preterm labour, their families and caregivers, using a qualitative framework approach. The outcomes of this qualitative study will inform the development of the mobile application decision support tool, using the findings from an individual patient data meta-analysis. The tool will then be externally validated and refined in the multi-centre trial, QUIDS.

For peer review only

For peer review only - http://bmjopen.bmj.com/site/about/guidelines.xhtml

Methods

A qualitative framework approach will be used, based on data collected from focus groups and semi-structured telephone interviews.

Setting

Focus groups will take place in three maternity units: Liverpool Women's NHS Foundation Trust, Birmingham Women's NHS Foundation Trust and Royal Edinburgh Hospital, NHS Lothian. There will be focus groups for women and a separate focus group for partners. Clinicians who care for women with threatened preterm birth will be interviewed by telephone.

Sample

A purposive sample of women and partners will be recruited to cover a variety of experiences of preterm labour and birth. Women will be stratified by their prior experience and relevant characteristics, including ethnicity, previous obstetric history, living in an urban or rural setting and proximity to a tertiary neonatal referral centre. Two focus groups of 4–8 women will be conducted at each site; one for pregnant women who are at high risk of preterm birth, and one for postnatal women who have recently experienced preterm birth. One partners' focus group will be conducted at one of the sites. If women or partners are unable to attend a focus group but still wish to participate, a semi-structured telephone interview will be offered.

Up to 10 obstetricians, including trainees, midwives, and neonatologists will be purposefully recruited to cover a range of professional backgrounds and experience. Semi-structured telephone interviews will be used to collect the data.

For peer review only - http://bmjopen.bmj.com/site/about/guidelines.xhtml

For peer review only

Eligibility

Principal inclusion criteria for women's antenatal focus groups

Women who are currently pregnant who:

- Have previously experienced preterm birth following preterm labour,
- Have experienced threatened preterm labour in this pregnancy,
- Are at high risk of preterm birth for another clinical reason, such as prior cervical surgery.

Principal inclusion criteria for women's postnatal focus groups

Women who have experienced preterm birth following preterm labour at <34 weeks whose babies **are stable and well** and are receiving care on the special care baby unit or neonatal intensive care unit.

Principal inclusion criteria for partners' focus groups

Partners of women who fit the eligibility criteria for either focus group.

Principal exclusion criteria for the focus groups

Non-English speaking individuals.

Principal inclusion criteria for clinician interviews

Clinicians who care for pregnant women i.e. obstetricians (including trainees), neonatologists and midwives.

Principal exclusion criteria for clinician interviews

For peer review only - http://bmjopen.bmj.com/site/about/guidelines.xhtml

Researchers in QUIDS or QUIDS qualitative.

Recruitment

Women and partners

Eligible women will be identified by clinicians in the preterm birth clinic and other antenatal clinics, and antenatal, triage or labour wards (for the antenatal focus groups) and the special care baby unit or postnatal clinics (for the postnatal focus groups) at each site. Eligible partners will be identified by the same method. Clinicians who are aware of and understand the research aims will approach women and partners to request consent for a researcher to contact them. Importantly, only postnatal parents whose babies are being cared for on the SCBU who are considered stable and well by the clinicians will be approached. With consent the researcher will make contact to talk to the women and/or their partners about the research, either face-to-face or over the telephone. Potential participants will be given the participant information sheet (PIS) (appendix _) that is relevant to them and given verbal information about the study. Each participant will be given time to read the information and the opportunity to have any questions answered. Willing participants will be asked to provide their written consent prior to the focus groups.

Clinicians

Eligible clinicians will be approached by the researchers, via email or face-to-face. Clinicians will be given the clinician PIS (appendix _) and the opportunity to read the information and have any questions answered. Willing clinicians will be asked to provide their written consent prior to the interviews.

For peer review only - http://bmjopen.bmj.com/site/about/guidelines.xhtml

All participants (women, partners and clinicians) will be reassured that they are not compelled to participate, that they can withdraw from the study at any time, and that non-participation will not affect their care or employment in any way.

Data collection

The primary aim of this research is to determine the decisional requirements of women, their partners and clinicians for the management of preterm labour. Qualitative semi-structured interviews, in a focus-group setting or individual telephone interviews, provide a means of collecting rich, in-depth data with a specific focus.[37] Hence, structured topic guides will be used to initiate and concentrate the discussion (appendices 7–10).

Focus groups are the preferred format for eliciting the view of women and women's partners. Encouraging discussion among a homogenous group with a shared interest is likely to provide rich insight and understanding into the group's experiences, beliefs and norms as a result of their social interaction.[38] Conversely, interviewing clinicians individually avoids the potential pitfall of professional embarrassment stifling ideas in a group setting. Interviewing individual clinicians with a range of professional experience should ensure that the decisional requirements of clinicians at all levels of experience are understood.

Demographic details and baseline characteristics will be collected prior to the interviews, either as a self-completion questionnaire, or questions asked by the researcher over the telephone. All interviews will be audio recorded, with the participants' consent, and field notes taken. The focus groups will be facilitated by at least two researchers. This is to ensure that all pre-specified areas of interest are covered and that non-verbal communication and group interactions are documented within the field-notes, which will provide context for the data analysis. Recapping will be used to

For peer review only - http://bmjopen.bmj.com/site/about/guidelines.xhtml

clarify aspects and avoid misinterpretation. To enable all participants to talk freely, the researchers will be unknown to the participants and not working clinically in the unit where the interview is conducted. Clinicians will be interviewed by a researcher who is unknown to them.

	Site	Interviewers
Women and partners' focus groups	Liverpool	HW and EO
	Birmingham	HW and VH-M
	Edinburgh	HW and LM
Clinician interviews	Telephone	HW (and EO?)

Analysis plan

A framework approach to data analysis will be used. This approach was developed to manage and interpret large volumes of data collected to inform health policy, meaning they had focussed aims and objectives.[37] Likewise, this research has clear aims, as described previously, in addition to the methodological aim of collecting rich data about the experiences and beliefs of women, their partners and clinicians in relation to managing preterm labour.

Framework analysis follows specific, clearly documented stages of analysis that are transparent so that others can review the interpretation processes and understand how the findings were reached.[39] Transparency is particularly important in this study as the findings will inform the development of an application to aid management decisions in clinical practice. Following verbatim transcription of the interview recordings, the researchers will become familiar with the data by reading the transcripts and field-notes several times. The next stage is to develop a theoretical framework by re-reading the transcripts and making notes as recurring characteristics are

For peer review only - http://bmjopen.bmj.com/site/about/guidelines.xhtml

recognised. The characteristics will then be collated into themes, which are based on the text itself, supported by the field-notes. The resulting thematic framework will be applied back to the transcripts and field-notes to check that it reflects the context of the original data. The transcripts will be coded, so that portions of text are linked to a discrete theme. A sample of transcripts will be independently coded by two people. The data will be charted and indexed to identify the preterm labour or professional experience of the participant, thus enabling the attribution of themes to a particular group. Finally, the content of the charts will be interpreted and mapped against each other to devise themes and sub-themes categories. Once again, this will involve review of the original data. Explanatory accounts will be developed to clarify the data and quotable sections of data will be identified. The final categories will be discussed between the researchers until consensus is met. The researchers will maintain reflexive journals throughout the data collection and analysis stages, recognising and ameliorating, as far as possible, the fact that their presence and assumptions impact on the data and the findings.[40]

This method of data analysis creates a clear audit trail thus ensuring rigour. Each stage of analysis refers back to the original data so that context and meaning is not lost in the final framework of themes and subthemes. The data analysis process will be managed using NVivo software, a qualitative data analysis tool.

Participant withdrawal

Participants may withdraw from the study at any point. However, they will not be able to withdraw use of their data once the prognostic tool is developed.

For peer review only - http://bmjopen.bmj.com/site/about/guidelines.xhtml

Safety

The physical safety of participants will be ensured through adhering to the health and safety policies of the host units where the focus groups take place.

The emotional wellbeing of the participants will be safeguarded by following the Distress Policy (see appendix 11). The Supervisors of Midwives (SOM) team in each unit will be informed of the study and women and their partners will be given the SOM team contact details, should they become distressed or upset as a result of talking about their experiences. Participants will also be given the contact details for accessing local counselling services.

Good clinical practice

Informed consent

All participants will be fully informed about the study and the subsequent QUIDS trial via verbal and written communication. All eligible individuals will be given the participant information sheet (appendix __) and provided with an opportunity to have any questions answered. Written consent will then be gained prior to the commencement of the focus groups/interviews.

Confidentiality

Demographic information will be collected from participants to attribute themes from the data to particular groups within the analysis and dissemination of findings. Demographic information, which will contain potentially identifiable information, will be kept in a secure lockable cabinet. Audio recordings will be stored on an encryptable audio device only until they are transcribed. Once transcribed the audio recordings will be deleted. Transcription services are provided by '1st Class Secretarial', who subscribe to the Data Protection Act and have also signed the Code of Practice on Data Handling. Hard copies of audio transcripts and field-notes will be kept in a separate secure

For peer review only - http://bmjopen.bmj.com/site/about/guidelines.xhtml

lockable cabinet to the demographic information. The transcripts and field-notes will be coded to identify which participant provided that data; the codes will only be known by the researchers. Participant's data will not be used for any purpose other than this study and the subsequent QUIDS trial.

Data Protection

Participants will be informed that publications from this study will contain direct quotes from the focus groups/interviews and categorisation of their experience of preterm labour (e.g. experienced preterm birth), which could enable personal identification.

All researchers involved in this study must comply with the requirements of the Data Protection Act 1998 with regard to the collection, storage, processing and disclosure of personal information and uphold the Act's core principles. All computers used for processing data are password protected and subject to the strict data protection policies of the researcher's institution.

Good clinical practice training

All researchers involved in this study must hold evidence of recent Good Clinical Practice training.

Additional ethical considerations

Expenses and reimbursement

Participants will be reimbursed for all out of pocket expenses, for example travelling to the interview site. Participants will be informed of this and how to apply for expenses reimbursement, including keeping receipts for travel.

For peer review only - http://bmjopen.bmj.com/site/about/guidelines.xhtml

Safety of researchers

An individualised risk assessment will be conducted to identify any risks to researchers or participants involved in this study. The lone working policy of the institution will be adhered to at all times. The only anticipated lone working will be during travel to and from the interview sites.

The lone working policy of the researcher's institutions mandates that researchers wear a GPS tracking and audio transmitting device during all lone-working, off-site research activity with participants. Participants will be informed if this device is being used.

Insurance / Indemnity

The researcher's institution holds public liability insurance and professional indemnity insurance (appendices 12, 13 and 14).

Timeline

The anticipated start date for the focus groups and interviews is 1st January 2016, to be completed within 3 months.

For peer review only - http://bmjopen.bmj.com/site/about/guidelines.xhtml

Appendices

Appendix 1: PIS women

Appendix 2: PIS partners

Appendix 3: PIS clinicians

Appendix 4: Consent form women

Appendix 5: consent form partners

Appendix 6: consent form clinicians

Appendix 7: Interview schedule AN women

Appendix 8: Interview schedule PN women

Appendix 9: Interview schedule partners

Appendix 10: Interview schedule clinicians

For peer review only

V 1.3
21/10/15

Appendix 11: Distress policy

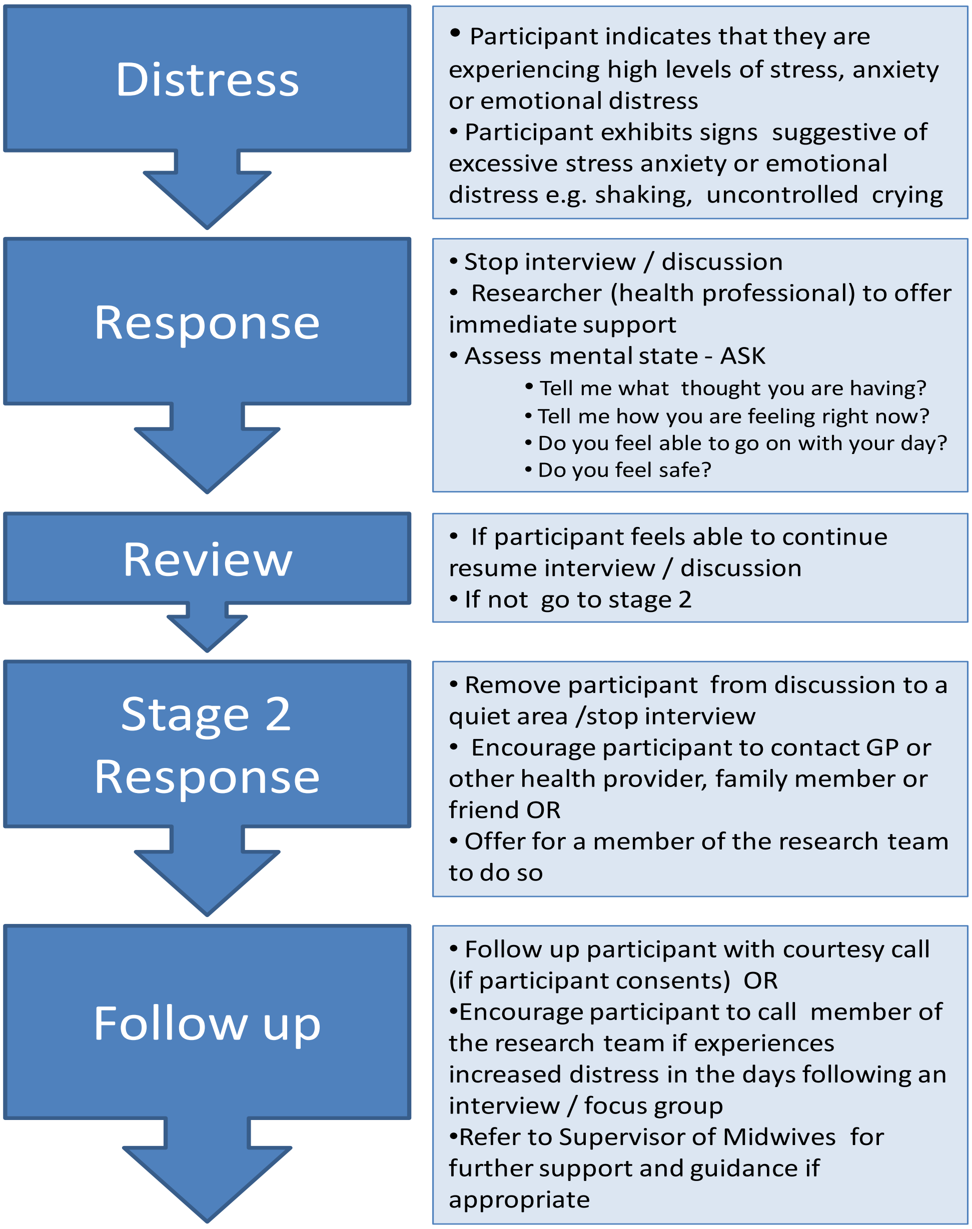

Adapted from Haigh and Witham (2010)[41]

V 1.3
21/10/15

Appendix 12: Public Liability insurance

To Whom It May Concern

Our ref: SP/IND 3 June, 2015

Zurich Municipal Customer: The University of Manchester

This is to confirm that The University of Manchester have in force with this Company Public Liability Insurance until the policy expiry on 31 May 2016:

Policy Number: NHE-07CA03-0013

Limit of Indemnity: £ 50,000,000 any one claim

Excess: Nil any one claim

Yours faithfully

Underwriting Services
Zurich Municipal
Farnborough

Zurich Municipal
Zurich House
2 Gladiator Way
Farnborough
Hampshire
GU14 6GB

Telephone 0870 2418050
Direct Phone 01252 387859
Direct Fax 01252 375893
E-mail alison.cliff@uk.zurich.com

Communications will be monitored regularly to improve our service and for security and regulatory purposes

Zurich Municipal is a trading name of Zurich Insurance Group Ltd

A public limited company incorporated in Ireland. Registration No. 13460 Registered Office: Zurich House, Ballsbridge Park, Dublin 4, Ireland.

UK branch registered in England and Wales Registration No. BR7985. UK Branch Head Office: The Zurich Centre, 3000 Parkway, Whiteley, Fareham, Hampshire PO15 7JZ

Authorised by the Central Bank of Ireland and subject to limited regulation by the Financial Conduct Authority. Details about the extent of our regulation by the Financial Conduct Authority are available from us on request.

V 1.3
21/10/15

Appendix 13: Employers' Liability insurance

ZURICH MUNICIPAL

Certificate of Employers' Liability Insurance(a)

(Where required by regulation 5 of the Employers' Liability (Compulsory Insurance) Regulations 2008 (the Regulations), a copy of this certificate must be displayed at all places where you employ persons covered by the policy or an electronic copy of the certificate must be retained and be reasonably accessible to each employee to whom it relates).

Policy No.	NHE-07CA03-0013
1. Name of policyholder	The University of Manchester
2. Date of commencement of insurance policy	01 June 2015
3. Date of expiry of insurance policy	31 May 2016

We hereby certify that subject to paragraph 2:

1. The policy to which this certificate relates satisfies the requirements of the relevant law applicable in Great Britain, Northern Ireland, the Isle of Man, the Island of Jersey, the Island of Guernsey and the Island of Alderney **(b)**

2. **(a)** the minimum amount of cover provided by this policy is no less than £5 million **(c)**

Signed on behalf of Zurich Insurance plc (Authorised Insurer).

Signature

S. Lewis

Stephen Lewis

Chief Executive Officer, Zurich Insurance plc (UK Branch)

Zurich Municipal is a trading name of Zurich Insurance plc A public limited company incorporated in Ireland Registration No.13460 Registered Office Zurich House, Ballsbridge Park ,Dublin 4 Ireland. UK branch registered in England and Wales Registration No. . BR 7985 UK Branch Head Office The Zurich Centre, 3000 Parkway, Whiteley, Fareham, Hampshire PO15 7JZ

Authorised by the Central Bank of Ireland and subject to limited regulation by the Financial Conduct Authority. Details about the extent of our regulation by the Financial Conduct Authority are available from us on request

Notes

(a) Where the employer is a company to which regulation 3(2) of the Regulations applies, the certificate shall state in a prominent place, either that the policy covers the holding company and all its subsidiaries, or that the policy covers the holding company and all its subsidiaries except any specifically excluded by name, or that the policy covers the holding company and only the named subsidiaries.

(b) Specify applicable law as provided for in regulation 4(6) of the Regulations.

(c) See regulation 3(1) of the Regulations and delete whichever of paragraphs 2(a) or 2(b) does not apply. Where 2(b) is applicable, specify the amount of cover provided by the relevant policy

V 1.3
21/10/15

Appendix 14: Professional indemnity insurance

MARSH

Cliffe Crowther
Marsh Ltd
Belvedere
12 Booth Street
Manchester
M2 4AW
+44 (0) 161 954 7317
Fax +44 (0) 161 954 7210
Cliffe.crowther@marsh.com
www.marsh.com

To whom it may concern

29th May 2015

Dear Sirs,

CONFIRMATION OF INSURANCE – The University of Manchester and Subsidiary Companies

As requested by the above client, we are writing to confirm that we act as Insurance Brokers to the client and that we have arranged insurance(s) on its behalf as detailed below:

PROFESSIONAL INDEMNITY INSURANCE

INSURERS	Novae Underwriting Ltd.
POLICY NUMBER	003210MMA15C
PERIOD OF INSURANCE	01 June 2015 to 31st May 2016, both dates inclusive.
LIMIT OF INDEMNITY	GBP10,000,000 any one claim and in the aggregate any one insurance period plus costs and expenses.
DEDUCTIBLE	GBP20,000 each & every claim including costs and expenses

INSURANCE BROKERS CII CHARTERED

Registered in England Number: 1507274, Registered Office:
1 Tower Place West, Tower Place, London EC3R 5BU.
Marsh Ltd is authorised and regulated by the Financial Conduct Authority

MARSH & McLENNAN COMPANIES

V 1.3
21/10/15

Page 2
29th of May 2015

We have placed the insurance which is the subject of this letter after consultation with the client and based upon the client's instructions only. Terms of coverage, including limits and deductibles, are based upon information furnished to us by the client, which information we have not independently verified.

This letter is issued as a matter of information only and confers no right upon you other than those provided by the policy. This letter does not amend, extend or alter the coverage afforded by the policies described herein. Notwithstanding any requirement, term or condition of any contract or other document with respect to which this letter may be issued or pertain, the insurance afforded by the policy (policies) described herein is subject to all terms, conditions, limitations, exclusions and cancellation provisions and may also be subject to warranties. Limits shown may have been reduced by paid claims.

We express no view and assume no liability with respect to the solvency or future ability to pay of any of the insurance companies which have issued the insurance(s).

We assume no obligation to advise yourselves of any developments regarding the insurance(s) subsequent to the date hereof. This letter is given on the condition that you forever waive any liability against us based upon the placement of the insurance(s) and/or the statements made herein with the exception only of wilful default, recklessness or fraud.

This letter may not be reproduced by you or used for any other purpose without our prior written consent.

This letter shall be governed by and shall be construed in accordance with English law.

Yours faithfully

Cliffe Crowther

MARSH & McLENNAN COMPANIES

V 1.3
21/10/15

References

1. Beck S, Wojdyla D, Say L, *et al.* The worldwide incidence of preterm birth: a systematic review of maternal mortality and morbidity. *Bull World Health Organ* 2010;88:31–38. doi:10.2471/BLT.08.062554.
2. Health and Social Care Information Centre. *Hospital Episode Statistics: NHS Maternity statistics — England, 2013–14.* Leeds, HSIC.
3. Saigal S, Doyle LW. An overview of mortality and sequelae of preterm birth from infancy to adulthood. *Lancet* 2008;371:261–9.
4. D'Onofrio BM, Class QA, Rickert ME, *et al*. Preterm birth and mortality and morbidity: a population-based quasi-experimental study. *JAMA Psychiatry* 2013; 70(11):1231-1240. doi:10.1001/jamapsychiatry.2013.2107 .
5. Mangham LJ, Petrou S, Doyle LW, *et al.* The cost of preterm birth throughout childhood in England and Wales. *Pediatrics* 2009;123(2):e312–27. doi: 10.1542/peds.2008-1827.
6. Alfirevic Z, Allen-Coward H, Molina F, *et al.* Targeted therapy for threatened preterm labor based on sonographic measurement of the cervical length: a randomized controlled trial. *Ultrasound Obstet Gynecol* 2007;29:47–50. DOI: 10.1002/uog.3908.
7. Salim R, Garmi G, Nachum Z, *et al.* Nifedipine compared with atosiban for treating preterm labor: a randomized controlled trial. *Obstet Gynecol* 2012;120:1323–31. DOI:10.1097/AOG.0b013e3182755dff
8. Roberts D, Dalziel SR. Antenatal corticosteroids for accelerating fetal lung maturation for women at risk of preterm birth. *Cochrane Database of Systematic Reviews* 2006, Issue 3. Art. No.: CD004454. DOI: 10.1002/14651858.CD004454.pub2.
9. Crowther CA, McKinlay CJD, Middleton P, Harding JE. Repeat doses of prenatal corticosteroids for women at risk of preterm birth for improving neonatal health outcomes.

For peer review only - http://bmjopen.bmj.com/site/about/guidelines.xhtml

Cochrane Database of Systematic Reviews 2015, Issue 7. Art. No.: CD003935. DOI: 10.1002/14651858.CD003935.pub4.

10. Doyle LW, Crowther CA, Middleton P, Marret S, Rouse D. Magnesium sulphate for women at risk of preterm birth for neuroprotection of the fetus. *Cochrane Database of Systematic Reviews* 2009, Issue 1. Art. No.: CD004661. DOI:10.1002/14651858.CD004661.pub3.
11. Royal College of Obstetricians and Gynaecologists. *Tocolysis for women in preterm labour: Green-top Guideline No. 1b.* 2011. London: RCOG.
12. Murphy KE, Hannah ME, Willan AR, Hewson SA, Ohlsson A, Kelly EN, Matthews SG, Saigal S, Asztalos E, Ross S, Delisle M-F, Amankwah K, Guselle P, Gafni A, Lee SK, Armson BA, for the MACS Collaborative Group. Multiple courses of antenatal corticosteroids for preterm birth (MACS): a randomised controlled trial. *Lancet* 2008; 372: 2143–51.
13. Norberg H, Stålnacke J, Diaz Heijtz R, Smedler A-C, Nyman M, Forssberg H, Norman M. Antenatal corticosteroids for preterm birth: dose-dependent reduction in birthweight, length and head circumference. *Acta Paediatrica* 2011;100:364–9.
14. Asztalos E, Willan A, Murphy K, Matthews S, Ohlsson A, Saigal S, Armson A, Kelly E, Delisle M-F, Gafni A, Lee S, Sananes R, Rovet J, Guselle P, Amankwah K, for the MACS-5 Collaborative Group. Association between gestational age at birth, antenatal corticosteroids, and outcomes at 5 years: multiple courses of antenatal corticosteroids for preterm birth study at 5 years of age (MACS-5). *BMC Pregnancy and Childbirth* 2014; 14:272.
15. Jacquemyn Y, Zecic A, Van Laere D, Roelens K. The use of intravenous magnesium in non-preeclamptic pregnant women: fetal/neonatal neuroprotection. *Arch Gynecol Obstet* 2015;291:969–975.
16. Gyetvai K, Hannah ME, Ellen D, Hodnett ED, Ohlsson A. Tocolytics for preterm labour: a systematic review. *Obstetrics and Gynecology* 1999;94(5):869–77.
17. Hill WC. Risks and complications of tocolysis. *Clinical Obstetrics and Gynecology* 1995;38(4):725–45.

For peer review only - http://bmjopen.bmj.com/site/about/guidelines.xhtml

18. Kent RA, Yazbek M, Heyns T, et al. The support needs of high-risk antenatal patients in prolonged hospitalisation. *Midwifery* 2015;31:164–9.
19. Lasswell SM, Barfield WD, Rochat RW, Blackmon L. Perinatal Regionalization for Very Low-Birth-Weight and Very Preterm Infants: A Meta-analysis. *JAMA.* 2010;304(9):992-1000. doi:10.1001/jama.2010.1226.
20. Marlow N, Bennett C, Drasper ES, Hennessy EM, Morgan AS, Costeloe KL. Perinatal outcomes for extremely preterm babies in relation to place of birth in England: The EPICure 2 study. *Arch Dis Child Fetal Neonatal Ed* 2014;99:F181–88.
21. Towers TV, Bonebrake R, Padilla G, Rumney P. The effect of transport on the rate of severe intraventricular hemorrhage in very low birth weight infants. *Obstetrics and Gynecology* 2000;95(2):291–295.
22. Porcellato L, Masson G, O'Mahony F, Jenkinson S, Vanner T, Cheshire K, Perkins E. 'It's something you have to put up with'— service users' experiences of in utero transfer: a qualitative study. *BJOG* 2015; DOI: 10.1111/1471-0528.13235.
23. Bond PA, Crisp AS, Morgan MEI, Lobb MO, Cooke RWI. Maternal attitudes to transfer before delivery. *Journal of Reproductive and Infant Psychology* 1984;2(1):33–41. DOI: 10.1080/02646838408403447.
24. Wilson AM, MacLean D, Skeoch CH, Jackson L. An evaluation of the financial and emotional impact of *in utero* transfers upon families: a Scotland-wide audit. *Infant* 2010;6(2):38–40.
25. Coster-Schulz MA, Mackey MC. The preterm labour experience: a balancing act. *Clinical Nursing Research* 1998;335–59.
26. Gale C, Hay A, Phillip C, et al. In-utero transfer is too difficult: results from a prospective study. *Early Human Development* 2012;88:147–50.
27. Macintyre-Beon C, Skeoch C, Jackson J, Booth P, Cameron A. *Perinatal Collaborative Transport Study: Final report.* 2008. Glasgow: Scottish Neonatal Transport Service.

V 1.3
21/10/15

For peer review only

28. O'Brien ET, Quenby S, Lavender T. Women's view of high risk pregnancy under threat of preterm birth. *Sexual and Reproductive Health* 2010;1:79–84.

29. Hezelgrave NL, Shennan AH, David AL. Tests to predict imminent delivery in threatened preterm labour. *BMJ* 2015;350:h2183 doi: 10.1136/bmj.h2183.

30. Chandiramani M, Di Renzo GC, Gottschalk E, Helmer H, Henrich W, Hoesli I, Mol B, Norman JE, Robson S, Thornton S, Shennan A. Fetal fibronectin as a predictor of spontaneous preterm birth: a European perspective. *The Journal of Maternal-Fetal & Neonatal Medicine* 2011;24(2):330–336.

31. Deshpande SN, van Asselt ADI, Tomini F, Armstrong N, Allen A, Noake C, et al. Rapid fetal fibronectin testing to predict preterm birth in women with symptoms of premature labour: a systematic review and cost analysis. *Health Technol Assess* 2013;17(40).

32. Radford S, Abbott D, Seed P, Kemp J, Shennan. Quantitative fetal fibronectin for the prediction of preterm birth in symptomatic women. *Arch Dis Child Fetal Neonatal Ed* 2012;97(Supp 1):A1–A125.

33. Harrison MJ, Kushner KE, Benzies K, et al. Women's satisfaction with their involvement in health care decisions during a high-risk pregnancy. *Birth* 2003;30(2):109–15.

34. Hill JL, Campbell MK, Zou GY, et al. Prediction of preterm birth in symptomatic women using decision tree modelling for biomarkers. *Am J Obstet Gynecol* 2008;198:468.e1-468.e9.

35. Gallagher K, Martin J, Keller M, et al. European variation in decision-making and parental involvement during preterm birth. *Arch Dis Child Fetal Neonatal Ed* 2014;99:F245–9.

36. Peerzada JM, Schollin J, Håkansson S. Delivery room decision-making for extremely preterm infants in Sweden. *Pediatrics* 2006;117(6):1988–95.

37. Smith J, Firth J. Qualitative data analysis: the framework approach. *Nurse Researcher* 2011;18(2):52–62.

38. Rabiee F. Focus group interview and data analysis. *Proceedings of the Nutrition Society* 2004;63: 655–660.

For peer review only - http://bmjopen.bmj.com/site/about/guidelines.xhtml

39. Furber C. Framework analysis: a method for analysing qualitative data. *African Journal of Midwifery and Women's Health* 2010;4(2):97–100.

40. Sword W. Accounting for presence of self: reflections on doing qualitative research. *Qualitative Health Research* 1999;9(2):270–8.

41. Haigh C, Witham G. *Distress protocol for qualitative data collection.* 2015. [Online] available at: http://www2.mmu.ac.uk/media/mmuacuk/content/documents/rke/Advisory%20Distress%20Protocol.pdf accessed October 2015.

For peer review only

V 1.3
21/10/15
